# Diagonal Interactions between Glutamate and Arginine Analogs with Varying Side-Chain Lengths in a β-Hairpin

**DOI:** 10.3390/molecules28072888

**Published:** 2023-03-23

**Authors:** Nian-Zhi Li, Chen-Hsu Yu, Jhuan-Yu Wu, Shing-Jong Huang, Shou-Ling Huang, Richard P. Cheng

**Affiliations:** 1 Department of Chemistry, National Taiwan University, Taipei 10617, Taiwan; 2 Instrumentation Center, National Taiwan University, Taipei 10617, Taiwan

**Keywords:** peptide, β-hairpin, diagonal interaction, ion-pairing interaction, charged amino acid, side-chain length

## Abstract

Cross-strand interactions are important for the stability of β-sheet structures. Accordingly, cross-strand diagonal interactions between glutamate and arginine analogs with varying side-chain lengths were studied in a series of β-hairpin peptides. The peptides were analyzed by homonuclear two-dimensional nuclear magnetic resonance methods. The fraction folded population and folding free energy of the peptides were derived from the chemical shift data. The fraction folded population trends could be rationalized using the strand propensity of the constituting residues, which was not the case for the peptides with lysine analogs, highlighting the difference between the arginine analogs and lysine analogs. Double-mutant cycle analysis was used to derive the diagonal ion-pairing interaction energetics. The most stabilizing diagonal cross-strand interaction was between the shortest residues (i.e., Asp2–Agp9), most likely due to the least side-chain conformational penalty for ion-pair formation. The diagonal interaction energetics in this study involving the arginine analogs appears to be consistent with and extend beyond our understanding of diagonal ion-pairing interactions involving lysine analogs. The results should be useful for designing β-strand-containing molecules to affect biological processes such as amyloid formation and protein-protein interactions.

## 1. Introduction

The β-sheet is one of the major secondary structures in proteins, with around 25% of the residues adopting a β-sheet conformation [1,2,3]. However, abnormal aggregation of β-sheets occurs during amyloid fibril formation, which is implicated in several diseases, including Alzheimer’s disease [4,5], Huntington’s disease [6], Parkinson’s disease [7], and type 2 diabetes mellitus [8,9,10]. As such, fundamental studies on β-sheet-containing motifs might provide insight into understanding sheet formation, stability, and relevant interactions. Furthermore, β-strand-containing molecules have been used to reduce amyloid accumulation through β-sheet-forming interactions [11]. Similarly, β-strand-containing molecules have also been used to inhibit protein-protein interactions involving β-sheet formation [12,13].

A β-sheet consists of multiple extended polypeptide chains (i.e., β-strands) linked by β-turns or loops. β-Sheets involve hydrogen bonds between the carbonyl oxygens and amide hydrogens in the backbone of neighboring β-strands [14]. A β-hairpin is the simplest β-sheet structure, composed of two antiparallel β-strands linked by a loop or turn. The β-hairpin represents a reasonable model system to study the stabilizing factors of β-sheet structures [15]. There are several factors that contribute to β-hairpin stability, including the turn type, sheet propensity of the constituting residues, strand length, and cross-strand interactions.

Statistical analysis of protein structures suggested that cross-strand side-chain interactions are important for β-sheet structure stability [16,17]. Computational studies have revealed the importance of cross-strand interactions for the association between infinitely long strands [18]. Cross-strand electrostatic interactions involving the C-terminal backbone carboxylate have been studied experimentally in a β-hairpin [19], providing up to 1.2 kJ/mol stabilization. In the same study, cross-strand lateral hydrophobics and intrastrand hydrophobics were also determined collectively [19], stabilizing the β-hairpin by up to 0.8 kJ/mol. Cross-strand ***lateral*** interactions involve residues directly across one another on neighboring strands. The cross-strand lateral Phe-Phe interaction (Phe: phenylalanine) was determined in a β-hairpin, providing at least 0.55 kcal/mol stabilization [20]. Furthermore, cross-strand lateral Phe-Phe interactions were shown to be more stabilizing than lateral Glu-Lys (glutamate-lysine) interactions in a β-hairpin [21]. Nonetheless, a cross-strand lateral Glu-Lys ion pair provided 0.3 kcal/mol stability in a β-hairpin [22]. Along these lines, we have investigated the effect of charged amino acid side-chain length on lateral cross-strand ion-pairing interactions in β-hairpins [23,24,25]. Side-chain length matching was important for the lateral interaction between carboxylate- and ammonium-containing residues (i.e., Glu and Lys analogs, respectively) [23]. Interaction between the short side-chains mostly involved electrostatics [23], whereas the interaction between the long side-chains could involve hydrophobics. As such, the lateral interaction between carboxylate- and guanidinium-containing residues (i.e., Glu and Arg (arginine) analogs, respectively) with only long side-chains were stabilizing [24]. However, the lateral interaction between carboxylate- and guanidinium-containing residues with short side chains was no longer stabilizing [24], because the positive charge on the guanidinium group is more diffuse than the ammonium group, thereby weakening the potential electrostatic interaction.

Statistical studies on protein structures showed that cross-strand ***diagonal*** sequence patterns were more significant than ***lateral*** patterns for antiparallel β-sheets [17]. Furthermore, a diagonal cation-π interaction was observed in a β-hairpin [26], stemming from the inherent right-handed twist of β-sheets [27]. A ***lateral*** interaction would involve residue *i* on one strand and residue *j* on the neighboring strand [17]. Along these lines, a ***diagonal*** interaction would involve residue *i* on one strand and residue *j* + 2 or *j* − 2 on the neighboring strand [17]. Accordingly, diagonal cation-π interactions were studied in β-hairpins [28,29,30,31]. Furthermore, a diagonal cyclohexyl-butyl interaction stabilized a β-hairpin by 0.3 kcal/mol [32]. Additionally, a diagonal carbohydrate-π interaction in a β-hairpin stabilized the motif up to 0.8 kcal/mol [33,34]. However, there were no investigations on cross-strand diagonal ion-pairing interactions until we investigated the effect of charged amino acid side-chain length on diagonal cross-strand interactions between Glu and Lys analogs with varying side-chain lengths in a β-hairpin [35]. The results showed that a balance between the side-chain entropic penalty for ion-pair formation and the number of conformations that can support a diagonal ion-pair was important for optimal interaction [35]. However, the guanidinium-containing Arg analogs behave structurally differently compared to the ammonium-containing Lys analogs [3,24,36,37,38]. To provide a more complete picture of diagonal ion-pairing interactions, a systematic study on the effect of charged amino acid side-chain length on diagonal interactions between Glu and Arg analogs using NMR methods is presented herein.

## 2. Results

### 2.1. Peptide Design

**Figure 1:**
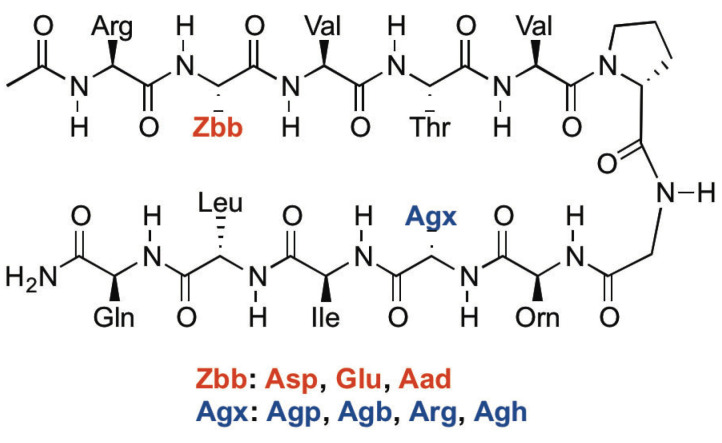
The chemical structure of the experimental HPDZbbAgx peptides. The three-letter code of the amino acid residues is used to represent the corresponding side chains for convenience in this figure.

**Figure 2:**
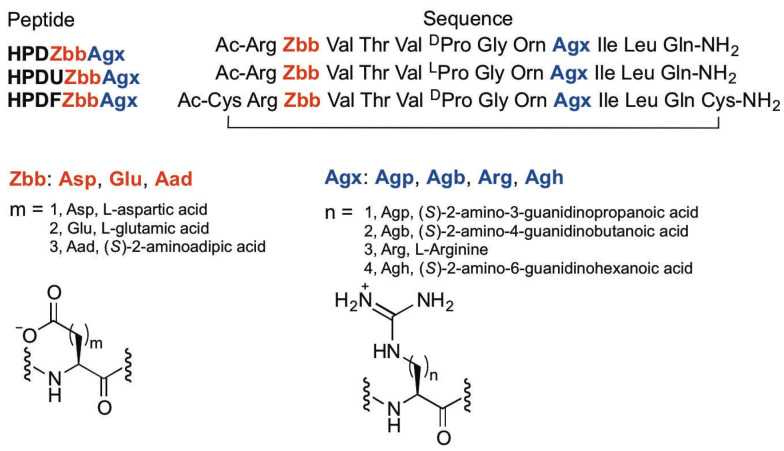
The sequences of the experimental HPDZbbAgx peptides, the fully unfolded reference HPDUZbbAgx peptides, and the fully folded reference HPDFZbbAgx peptides.

The experimental HPDZbbAgx peptides were designed based on Gellman’s YKL peptide [26], which was modified by our group [35] (Figure 1 and Figure 2). The DPro-Gly (D-proline-glycine) turn was used to promote a stable β-hairpin conformation [39]. Both the diagonal Tyr2-Lys9 (Tyr: tyrosine) interaction and the lateral Glu4-Lys9 interaction occurred in the YKL peptide [26]. As such, Glu4 was replaced with Thr (threonine) to remove the lateral Glu4-Lys9 ion-pairing interaction. The side chains of the amino acids at positions 2 and 9 point inward; residues 2 and 9 are both non-hydrogen-bonded positions (Figure 1). β-Sheets display an inherent right-handed twist [27], giving rise to the diagonal interactions between side-chains at positions 2 and 9 [26]. Negatively charged residues (Zbb = Asp, Glu, Aad; Figure 2) and positively charged residues (Agx = Agp, Agb, Arg, Agh; Figure 2) were incorporated at positions 2 and 9, respectively, to investigate the effect of charged amino acid side-chain length on the potential cross-strand diagonal Zbb2-Agx9 ion-pairing interaction (Figure 1 and Figure 2). The N-terminus was acetylated and the C-terminus was amidated to minimize unintended interactions with the termini [19]. The experimental HPDZbbAgx peptides were named with the “HPD” prefix, representing **H**air**P**in peptides to study the **D**iagonal interactions between Zbb and Agx at positions 2 and 9, respectively. Fully folded reference peptides and fully unfolded reference peptides were needed to determine the fraction folded population and the folding free energy for the experimental peptides [3,23,24,25,26,35,38]. The fully folded reference peptides were designed by adding a Cys (cysteine) residue at both the N- and C-terminus of the experimental HPDZbbAgx peptides to form an intramolecular disulfide bond to give the HPDFZbbAgx peptides [3,23,24,25,26,35,38] (Figure 2). For the fully unfolded reference peptides, the DPro in the experimental HPDZbbAgx peptides was replaced with LPro (L-proline) to disrupt β-hairpin formation and give the HPDUZbbAgx peptides [3,23,24,25,26,35,38] (Figure 2).

### 2.2. Peptide Synthesis and Purification

All peptides were synthesized by solid-phase peptide synthesis using Fmoc-based chemistry [40,41]. The Agh-containing peptides were synthesized by standard coupling protocols using Fmoc-Agh(Boc)_2_-OH [36,42]. Peptide HPDUAspAgb was synthesized by solid-phase guanidinylation of the precursor Dab(ivDde)-containing peptide (Dab: (*S*)-2,3-diaminobutyric acid; ivDde: 1-(4,4-dimethyl-2,6-dioxocyclohexylidene)-3-methyl-butyl) following published procedures [36]. However, the other Agb-containing peptides were synthesized by standard coupling protocols using Fmoc-Agb(Pbf, Boc)-OH (Pbf: 2,2,4,6,7-pentamethyldihydrobenzofuran-5-sulfonyl; Boc: *tert*-butyloxycarbonyl), due to difficulty in solid-phase guanidinylation and in the separation of the desired guanidinylated peptide from the non-guanidinylated peptide. Similarly, Fmoc-Agp(Pbf, Boc)-OH was used for synthesizing the Agp-containing peptides. The intramolecular disulfide bond in the Cys-containing HPDFZbbAgx peptides was formed by charcoal-mediated air oxidation [43]. The crude peptides were purified by reverse-phase high-performance liquid chromatography (RP-HPLC) to higher than 95% purity. All the peptides were confirmed by matrix-assisted laser desorption ionization time-of-flight (MALDI-TOF) mass spectrometry. 

### 2.3. NMR Characterization

All the peptides were analyzed by two-dimensional nuclear magnetic resonance (NMR) spectroscopy at 298 K, including total correlation spectroscopy (TOCSY) [44], double quantum filtered correlation spectroscopy (DQF-COSY) [45], and rotating frame nuclear Overhauser effect correlation spectroscopy (ROESY) [46]. Sequence-specific assignments for all peptides were completed based on the TOCSY and ROESY spectra (Tables S3–S38). Since the chemical shift and line width of the NMR spectra for analogous hairpin peptides did not change with concentration (20 μM to 10 mM) [3,26,47,48], the peptides in this study (1.4−8.9 mM) should not aggregate in solution. As such, the NMR data should primarily arise from intramolecular interactions with minimal contribution from intermolecular interactions. The β-hairpin structure of the peptides was confirmed by *^3^J_NHα_* spin-spin coupling constants, NOEs, and Hα chemical shift deviation (ΔδHα). 

The *^3^J_NHα_* coupling constant between the amide proton and α proton in the same residue was determined for the residues in the peptides using the corresponding DQF-COSY spectra [49]. The strand residues in the fully folded reference HPDFZbbAgx peptides and experimental HPDZbbAgx peptides exhibited *^3^J_NHα_* values higher than 7 Hz, consistent with the peptides forming a β-hairpin conformation (Tables S39–S47). If one disregards the standard deviations, the average *^3^J_NHα_* values for the strand residues (for a given Zbb2-Agx9 pair) would follow the trend HPDFZbbAgx > HPDZbbAgx > HPDUZbbAgx (Table S2), which was consistent with the intended β-conformation content for the peptides.

The NOE cross-peaks in the ROESY spectra were assigned. Intra-residue, sequential, and medium/long-range NOEs were observed (Figures S3–S50). Sequential Hα*_i_*–HN*_i_*_+1_ NOEs were observed for the strand region for all peptides (Figures S39–S50), consistent with an extended conformation for the residues in the strand regions. A network of cross-strand NOEs between residues on the adjacent strands was observed for both the fully folded reference HPDFZbbAgx peptides and the experimental HPDZbbAgx peptides (Figures S3–S38), suggesting the formation of a β-hairpin conformation. The diagonal cross-strand Thr4-Gly7 and Zbb2-Agx9 NOEs were observed in a number of the HPDFZbbAgx and HPDZbbAgx peptides, suggesting a right-handed twist of the β-hairpin structure [25,26,35]. Besides, the number of NOEs for a given Zbb2-Agx9 pair followed the trend HPDFZbbAgx > HPDZbbAgx > HPDUZbbAgx.

The Hα chemical shift deviation (ΔδHα) for the experimental HPDZbbXaa peptides and the fully folded reference HPDFZbbXaa peptides were derived by treating the unfolded reference HPDUZbbXaa peptides as random coil [26] (Figures S1 and S2). Generally, residues Zbb2 through Val5 and Orn8 through Leu11 in the HPDZbbAgx and HPDFZbbAgx peptides showed positive ΔδHα values, consistent with a β-hairpin conformation. For the terminal residues Arg1 and Gln12, extremely low ΔδHα values were observed due to the end-fraying effect [50]. The ΔδHα values of residue Gly7 were near zero or even negative, consistent with a turn structure. Furthermore, the ΔδHα for the strand residues in the folded reference HPDFZbbXaa peptides were more positive compared to the ΔδHα for the corresponding residues in the experimental HPDZbbXaa peptides, consistent with the intended folding extent for the HPDFZbbXaa and HPDZbbXaa peptides.

### 2.4. Fraction Folded Population and Folding Free Energy

The fraction folded population and folding free energy (ΔG_fold_) for each residue in the experimental peptides were derived from the Hα chemical shift data (Figures S51 and S52). Values for residues 2, 3, 9, and 10 for a given peptide were averaged to represent the fraction folded population and ΔG_fold_ for the peptide of interest [23,24,25,35,38] (Table 1 and Table 2). Since the fraction folded population and ΔG_fold_ are inter-related (i.e., the higher fraction folded population, the more negative the ΔG_fold_), only the fraction folded data will be discussed further.

The fraction folded population for the HPDZbbAgx peptides was between 16 and 51% (Table 1). HPDAadAgb exhibited the highest fraction folded population, whereas HPDAspAgh exhibited the lowest fraction folded population. The fraction folded population of the HPDZbbAgp peptides followed the trend HPDAspAgp < HPDGluAgp ~ HPDAadAgp. Similarly, the fraction folded population of the HPDZbbArg peptides followed the trend HPDAspArg < HPDGluArg ~ HPDAadArg. Additionally, the fraction folded population of the HPDZbbAgh peptides followed the trend HPDAspAgh < HPDGluAgh ~ HPDAadAgh. However, the fraction folded population of the HPDZbbAgb peptides followed the trend HPDAspAgb < HPDGluAgb < HPDAadAgb. If one disregards HPDAadAgb, the fraction folded population of the HPDZbbAgx peptides for a given positively charged residue Agx at position 9 would follow the trend HPDAspAgx < HPDGluAgx ~ HPDAadAgx.

The fraction folded populations of the four HPDAspAgx peptides were similar. However, the fraction folded population of the HPDGluAgx peptides followed the trend HPDGluAgp < HPDGluAgb > HPDGluArg~HPDGluAgh. Similarly, the fraction folded population of the HPDAadXaa peptides followed the trend HPDAadAgp < HPDAadAgb > HPDAadArg~HPDAadAgh. If one disregards HPDGluAgb and HPDAadAgb, the fraction folded population for HPDZbbAgx for a given negatively charged residue Zbb would be similar.

### 2.5. Diagonal Zbb2-Agx9 Ion-Pairing Interaction Energy

The interaction free energy (ΔG_int_) for the cross-strand diagonal Zbb2-Agx9 interactions was derived by double mutant cycle analysis [51] (Table 3). The cross-strand diagonal Zbb2-Agx9 ΔG_int_ was either stabilizing or non-existent. For the Asp2-Agx9 interactions, only the Asp2-Agp9 interaction was stabilizing. Furthermore, this Asp2-Agp9 interaction was the most stabilizing of the diagonal Xaa2-Agx9 interactions, providing 0.45 kcal/mol stabilization. In contrast, all Glu2-Agx9 interactions were stabilizing with similar interaction energetics. As for the Aad2-Agx9 interactions, only the Aad2-Agb9 interaction was stabilizing.

## 3. Discussion

The effect of side-chain length on cross-strand diagonal interactions between carboxylate- and guanidinium-containing residues has been studied in β-hairpin peptides. The chemical shift dispersion for each peptide was quantitatively gauged using the range and the standard deviation of the average for the HN and Hα chemical shifts (separately) (Table S1). Chemical shift dispersion describes the spread of the chemical shifts for particular nuclei in an NMR spectrum [52]. In general, the more folded the peptide/protein, the higher the chemical shift dispersion. For a given Zbb2-Agx9 pair, the range of the HN (and Hα) chemical shift followed the trend: HPDFZbbAgx > HPDZbbAgx > HPDUZbbAgx (Table S1). Similarly, the standard deviation for the average HN (and Hα) chemical shift followed the trend: HPDFZbbAgx > HPDZbbAgx > HPDUZbbAgx, for a given Zbb2-Agx9 pair (Table S1). As such, the chemical shift dispersion trends were consistent with the intended folded extents for the peptides.

The fraction folded population for the experimental HPDZbbAgx peptides with potential cross-strand diagonal Zbb2-Agx9 (carboxylate-guanidinium) ion-pairing interactions was determined based on NMR chemical shift data (Table 1). The fraction folded population for the HPDZbbAgx peptides in this study was 16–51% (Table 1). In contrast, the fraction folded population for the analogous HPTZbbAgx peptides, with potential lateral ion-pairing Zbb4-Agx9 (carboxylate-guanidinium) interactions, was 28–56% [24]. Interestingly, the fraction folded population for the HPDZbbXaa peptides, with potential diagonal ion-pairing Zbb2-Xaa9 (carboxylate-ammonium) interactions, was 15–47% [35]. The range of the fraction folded population for the HPDZbbAgx peptides in this study was slightly broader than either the HPTZbbAgx [24] or HPDZbbXaa peptides [35]. The peptide with the highest fraction folded population in this study was HPDAadAgb (with a potential diagonal Aad2-Agb9 interaction). However, the analogous HPTAadAgb peptide (with a potential lateral Aad4-Agb9 interaction) exhibited the lowest fraction folded population in the study on HPTZbbAgx peptides [24]. If one considers the error bars, the thermodynamic strand propensity of the residues at positions 2 and 9 [38] could rationalize the HPDZbbAgx peptides with the highest (HPDAadAgb) and lowest (HPDAspAgp~HPDAspAgh) fraction folded population in this study. Similarly, the HPTZbbAgx peptides (also involving guanidinium-containing Arg analogs) with the highest (HPTAspAgh) and the lowest (HPTAadAgb) fraction folded population [24] could also be rationalized by the thermodynamic strand propensity of the potentially interacting residues [3]. In contrast, simple thermodynamic strand propensity arguments could not explain the highest and lowest fraction folded populations for the HPDZbbXaa peptides involving ammonium-containing Lys analogs [35]. This suggests that the thermodynamic strand propensity plays an important role in the fraction folded population variation of the peptides with potential cross-strand interactions between carboxylate- and guanidinium-containing Arg analogs (both lateral and diagonal). This may be due to the more diffuse positive charge and higher hydrogen bonding capacity (for interaction with water) of the guanidinium group compared to the ammonium group. Importantly, if one wanted to diagonally pair with Arg, Glu and Aad would provide the most stability based on the fraction folded population data (Table 1). Similarly, if one wanted to diagonally pair with Asp or Glu, Agb would provide the most stability. Interestingly, only the Arg-Glu pairing involved two encoded amino acids, suggesting the importance of incorporating non-encoded amino acids to form stable β-sheets.

The diagonal Zbb2-Agx9 ion-pairing interaction energies were determined for the various side-chain length combinations using double-mutant cycle analysis (Table 3). In general, the side-chain functionality of the charged amino acids (i.e., carboxylate, guanidinium, or ammonium) could engage in electrostatic interactions and hydrogen bonds. In contrast, the hydrophobic linking methylenes could provide the sufficient length to enable the aforementioned interactions, or directly interact through hydrophobics. In this study, the diagonal Asp2-Agp9 interaction was the most stabilizing (−0.45 kcal/mol), suggesting that the short length of both Asp and Agp was long enough for ion-pair formation, but the interaction most likely did not involve hydrophobics. Furthermore, these two shortest residues would pay the least side-chain conformational entropic penalty to enable diagonal ion-pair formation compared to the longer residues. Strikingly, the lateral Asp4-Agp9 interaction was effectively non-existent (−0.03 ± 0.20 kcal/mol) [24], highlighting the difference in the orientation of side chains for lateral and diagonal interactions [35]. Lengthening the guanidinium-containing residue in the highly stabilizing Asp2-Agp9 diagonal interaction resulted in the lack of Asp2-Agb9 interaction (−0.07 ± 0.21 kcal/mol), whereas lengthening the carboxylate-containing residue resulted in a diminished but stabilizing Glu2-Agp9 interaction (−0.20 ± 0.06 kcal/mol). Lengthening the carboxylate-containing residue further resulted in the lack of Aad2-Agp9 interaction (0.06 ± 0.06 kcal/mol). Interestingly, all Glu2-Agx9 interactions were stabilizing, regardless of the side-chain length of the guanidinium-containing residue, suggesting a subtle balance between the side-chain conformational entropic penalty for ion-pair formation and the number of side-chain conformations accommodating the ion-pair. This is in sharp contrast to the lateral interactions between carboxylate- and guanidinium-containing residues [24], showing stabilizing interactions for only the longer side chains: Aad4-Arg9 (−0.25 kcal/mol) and Aad4-Agh9 (−0.22 kcal/mol). In this study, the only stabilizing diagonal interaction involving Aad is the Aad2-Agb9 interaction (−0.16 ± 0.05 kcal/mol). This is most likely due to the different side-chain orientation for cross-strand diagonal interactions compared to lateral interactions [35]. At first glance, the results in this study on diagonal interaction involving guanidinium-containing residues would appear to be different from our earlier study involving ammonium-containing residues [35], if one only considers the number of side-chain methylenes. However, the overall side-chain length of Agp (Cβ-Nγ-Cδ-Nε) is similar to Orn (Cβ-Cγ-Cδ-Nε) and the two should behave similarly. Indeed, the Asp2-Agp9 interaction (−0.45 ± 0.23 kcal/mol) was comparable to the Asp2-Orn9 interaction (−0.375 ± 0.081 kcal/mol). Furthermore, lengthening the Asp2 side chain resulted in diminished Glu2-Orn9 interaction (−0.291 ± 0.48 kcal/mol) [35], and further lengthening resulted in a lack of Aad2-Orn9 interaction (−0.023 ± 0.040 kcal/mol) [35]. As such, the diagonal ion-pairing interaction energetics in this study involving guanidinium-containing Arg analogs are consistent with those involving ammonium-containing Lys analogs [35]. Furthermore, the results provide insight into positively charged residues with even longer side-chains than the previously studied ammonium-containing Lys analogs [35]. Nonetheless, future studies involving changes in temperature, pH, or salt concentration may provide further insight into the nature of the interactions.

## 4. Materials and Methods

### 4.1. Peptide Synthesis and Purification

Peptides (in Figure 2) were synthesized by solid-phase peptide synthesis using Fmoc-based chemistry [40,41]. Peptide HPDUAspAgb was synthesized by solid-phase guanidinylation of the precursor Dab(ivDde)-containing peptide following published procedures [36]. Charcoal-mediated air oxidation was used to form the intramolecular disulfide bond in the HPDFZbbAgx peptides [43]. The peptides were purified by RP-HPLC to higher than 95% purity. All the peptides were confirmed by MALDI-TOF mass spectrometry.

### 4.2. Nuclear Magnetic Resonance Spectroscopy

The purified peptides were dissolved in pH 5.5 (uncorrected) 50 mM sodium deuterioacetate buffer in H_2_O/D_2_O (9:1 ratio by volume). 2-Dimethyl-2-silapentane-5-sulfonate was added to each sample as an internal standard. Concentrations of the peptide samples were 1.4–8.9 mM. The peptides were analyzed by two-dimensional NMR spectroscopy at 298 K, including TOCSY [44], DQF-COSY [45], and ROESY [46]. We chose to use NMR spectroscopy to investigate the peptide conformation in solution, because it provides atom and residue-specific information. The TOCSY spectra were used to determine the spin systems in the peptides. The DQF-COSY spectra were used to derive the *^3^J_NHα_* coupling constants, which are related to the ϕ dihedral angle, and thus backbone conformation/structure. The ROESY spectra were used to obtain inter-proton information. Sequence-specific assignments for all peptides were completed based on the TOCSY and ROESY spectra. *^3^J_NHα_* coupling constants were derived for the residues based on the DQF-COSY spectra [49]. The Hα chemical shift deviation (ΔδHα) for the residues was derived by treating the unfolded reference HPDUZbbXaa peptides as random coil [26]. The fraction folded population and the ΔG_fold_ for each residue were derived from the chemical shift data [26]. Values for residues 2, 3, 9, and 10 for a given peptide were averaged to represent the fraction folded population and ΔG_fold_ for the peptide of interest [23,24,25,35,38] (Table 1 and Table 2). Residues from both strands were included, with equal representation of the hydrogen-bonded sites (residues 3 and 10) and the non-hydrogen-bonded sites (residues 2 and 9). Double-mutant cycle analysis [51] was used to derive the cross-strand diagonal Zbb2-Agx9 ΔG_int_. The double-mutant cycle was used to remove the effect of individually incorporating each Zbb2 and Agx9 residue. In this analysis, the folding free energy of the experimental HPDZbbAgx peptides (Table 2) and the corresponding HPDZbbAla, HPDAlaAgx, and HPDAlaAla peptides [38] were used to determine the diagonal Zbb2-Agx9 ΔG_int_ (Table 3).

## 5. Conclusions

The effect of side-chain length on cross-strand diagonal interactions between Glu and Arg analogs with varying side-chain lengths has been studied. HPDAadAgb exhibited the highest fraction folded population, suggesting that sheet structures with a diagonal Aad-Agb would be most stable. Interestingly, the thermodynamic strand propensity of the Glu and Arg analogs [38] rationalized the fraction folded population trends in this study. This was not the case for the peptides with potential diagonal interactions involving Lys analogs [35], showcasing the difference between the guanidinium group (in this study) and the ammonium group. The diagonal Asp2-Agp9 interaction was the most stabilizing, most likely because these two shortest residues would pay the least side-chain conformational entropic penalty to enable diagonal ion-pair formation. In contrast, the lateral Asp4-Agp9 interaction was non-existent [24], confirming the difference in the orientation of side chains for lateral and diagonal interactions [35]. Importantly, the diagonal interaction energetics in this study involving guanidinium-containing Arg analogs is consistent with and extends beyond our understanding of the diagonal ion-pairing interactions involving ammonium-containing Lys analogs. These results should be useful for designing strand-containing molecular entities for biological applications involving sheet formation such as amyloid reduction [11] and protein-protein interaction inhibition [12,13].

## Figures and Tables

**Table 1:** The fraction folded population (%) for the HPDZbbAgx peptides.

Zbb2	Agx9			
	Agp	Agb	Arg	Agh
Asp	21 ± 4	19 ± 4	17 ± 5	16 ± 6
Glu	31 ± 2	41 ± 4	36 ± 4	35 ± 4
Aad	30 ± 3	51 ± 4	37 ± 5	39 ± 5

**Table 2:** The folding free energy (ΔG_fold_, kcal/mol) for the HPDZbbAgx peptides.

Zbb2	Agx9			
	Agp	Agb	Arg	Agh
Asp	0.789 ± 0.143	0.869 ± 0.163	0.942 ± 0.212	0.993 ± 0.261
Glu	0.473 ± 0.058	0.229 ± 0.098	0.334 ± 0.110	0.383 ± 0.103
Aad	0.496 ± 0.094	−0.020 ± 0.099	0.332 ± 0.123	0.272 ± 0.113

**Table 3:** The diagonal Zbb2–Agx9 interaction energy (ΔG_int_, kcal/mol) in the HPDZbbAgx peptides.

Zbb2	Agx9			
	Agp	Agb	Arg	Agh
Asp	−0.453 ± 0.227	−0.074 ± 0.208	−0.185 ± 0.258	−0.061 ± 0.124
Glu	−0.196 ± 0.055	−0.141 ± 0.070	−0.221 ± 0.145	−0.098 ± 0.042
Aad	0.061 ± 0.056	−0.156 ± 0.045	0.011 ± 0.172	0.026 ± 0.054

## Data Availability

The data presented in this study are available in the Supplementary Materials. The raw data are available on request from the corresponding author.

## Supplementary Materials

The following supporting information can be downloaded at: https://www.mdpi.com/article/10.3390/molecules28072888/s1, Table S1: The chemical shift range and average chemical shift of the δHN and δHα for the peptides; Table S2: The average *^3^J_NHα_* coupling constant values of the strand residues in the peptides; Tables S3–S38: The ^1^H chemical shift assignments for the peptides; Tables S39–S47: The *^3^J_NHα_* values of the peptides; Figure S1: The Hα chemical shift deviations for the residues in the experimental HPDZbbAgx peptides; Figure S2: The Hα chemical shift deviations for the residues in the fully folded reference HPDFZbbAgx peptides; Figures S3–S38: The inter-residue NOEs observed involving the side chains of the peptides; Figures S39–S50: Wüthrich diagrams of the backbone NOE connectivities involving the α-protons and amide protons for the peptides; Figure S51: The fraction folded population of the residues in the peptides; Figure S52: The ΔG_fold_ of the residues in the peptides. References [53,54,55,56] are cited in the Supplementary Materials.

## Sample Availability

Samples of the compounds are not available from the authors.
