# Supplementary material for: Diagonal Interactions between Glutamate and Arginine Analogs with Varying Side-Chain Lengths in a β-Hairpin
Source: Molecules. 2023 Mar 23;28(7):2888. doi: 10.3390/molecules28072888 (PMC10096425; doi:10.3390/molecules28072888)
Supplement: Supplementary file 1 [file molecules-28-02888-s001.zip › molecules-2228881-supplementary.pdf]

Supplementary Materials

Effect of Charged Amino Acid Side Chain Length on Diagonal Cross Strand Interactions between Carboxylate- and Guanidinium-Containing Residues in a β-Hairpin

Nian-Zhi Li, Chen-Hsu Yu, Jhaun-Yu Wu, Shing-Jong Huang, Shou-Ling Huang, Richard P. Cheng*

Tables

Table S1. The Chemical Shift Range and Average Chemical Shift of the δHN and δHα for the Peptides. S2

Table S2. The Average $^{3}J_{NH^{\alpha}}$ Coupling Constant Values of the Strand Residues in the Peptides. S3

Tables S3~S38. The ^{1}H Chemical Shift Assignments for the Peptides. S4~S21

Tables S39~S47. The $^{3}J_{NH^{\alpha}}$ Values of the Peptides. S23~S26

Figures

Figure S1. The Hα chemical shift deviations for the residues in the experimental HPDZbbAgx peptides. S27

Figure S2. The Hα chemical shift deviations for the residues in the fully folded reference HPDFZbbAgx peptides. S28

Figures S3~S38. The inter-residue NOEs observed involving the side chains of the peptides. S29~S40

Figures S39~S50. Wüthrich diagrams of the backbone NOE connectivities involving the α-protons and amide protons for the peptides. S41~S52

Figure S51. The fraction folded population of the residues in the peptides. S53

Figure S52. The ΔG_{fold} of the residues in the peptides. S54

Material and Methods S55~S68

References S69

Table S1. The Chemical Shift Range and Average Chemical Shift of the δHN and δHα for the HPDFZbbAgx, HPDZbbAgx, and HPDUZbbAgx Peptides.

Peptide	δHN Range[a] (ppm)	Average δHN (ppm)	δHα Range[a] (ppm)	Average δHα (ppm)
HPDFAspAgp	1.144	8.692 ± 0.336	1.319	4.637 ± 0.400
HPDAspAgp	0.645	8.416 ± 0.175	0.946	4.370 ± 0.272
HPDUAspAgp	0.573	8.379 ± 0.155	0.701	4.338 ± 0.196
HPDFAspAgb	1.167	8.650 ± 0.342	1.424	4.638 ± 0.400
HPDAspAgb	0.562	8.364 ± 0.157	0.899	4.347 ± 0.251
HPDUAspAgb	0.499	8.332 ± 0.143	0.688	4.316 ± 0.173
HPDFAspArg	1.164	8.630 ± 0.335	1.395	4.622 ± 0.393
HPDAspArg	0.484	8.337 ± 0.144	0.900	4.340 ± 0.251
HPDUAspArg	0.431	8.306 ± 0.127	0.733	4.279 ± 0.193
HPDFAspAgh	1.105	8.616 ± 0.328	1.088	4.651 ± 0.301
HPDAspAgh	0.445	8.322 ± 0.139	0.891	4.330 ± 0.247
HPDUAspAgh	0.297	8.297 ± 0.124	0.700	4.301 ± 0.170
HPDFGluAgp	1.109	8.716 ± 0.312	1.411	4.654 ± 0.404
HPDGluAgp	0.653	8.439 ± 0.172	0.992	4.379 ± 0.269
HPDUGluAgp	0.359	8.384 ± 0.127	0.700	4.317 ± 0.174
HPDFGluAgb	1.226	8.710 ± 0.350	1.477	4.646 ± 0.399
HPDGluAgb	0.633	8.456 ± 0.190	0.975	4.384 ± 0.266
HPDUGluAgb	0.318	8.363 ± 0.115	0.491	4.294 ± 0.144
HPDFGluArg	1.201	8.686 ± 0.348	1.461	4.637 ± 0.395
HPDGluArg	0.607	8.417 ± 0.177	1.000	4.368 ± 0.260
HPDUGluArg	0.343	8.334 ± 0.100	0.525	4.257 ± 0.167
HPDFGluAgh	1.217	8.679 ± 0.361	1.452	4.629 ± 0.389
HPDGluAgh	0.606	8.402 ± 0.174	0.969	4.359 ± 0.254
HPDUGluAgh	0.362	8.326 ± 0.102	0.498	4.282 ± 0.138
HPDFAadAgp	1.168	8.721 ± 0.316	1.437	4.666 ± 0.411
HPDAadAgp	0.660	8.450 ± 0.172	1.010	4.379 ± 0.270
HPDUAadAgp	0.376	8.395 ± 0.117	0.702	4.313 ± 0.175
HPDFAadAgb	1.242	8.716 ± 0.357	1.503	4.651 ± 0.404
HPDAadAgb	0.734	8.474 ± 0.217	1.019	4.410 ± 0.281
HPDUAadAgb	0.320	8.349 ± 0.103	0.493	4.296 ± 0.144
HPDFAadArg	1.197	8.690 ± 0.349	1.474	4.641 ± 0.395
HPDAadArg	0.601	8.409 ± 0.175	0.955	4.370 ± 0.253
HPDUAadArg	0.260	8.322 ± 0.083	0.500	4.286 ± 0.139
HPDFAadAgh	1.218	8.683 ± 0.361	1.455	4.631 ± 0.387
HPDAadAgh	0.620	8.399 ± 0.177	0.948	4.367 ± 0.253
HPDUAadAgh	0.275	8.312 ± 0.080	0.499	4.282 ± 0.139

[a]The chemical shift range was the chemical shift difference between the most downfield signal and the most upfield signal for the proton of interest.

Table S2. The Average $^{3}J_{NH\alpha}$ Coupling Constant Values for the Strand Residues in the HPDFZbbAgx, HPDZbbAgx, and HPDUZbbAgx Peptides.

Zbb	Agx	HPDFZbbAgx	HPDZbbAgx	HPDUZbbAgx
Asp	Agp	10.90 ± 0.32	9.77 ± 0.61	9.54 ± 0.33
Asp	Agb	11.00 ± 0.00	10.56 ± 0.72	9.50 ± 0.77
Asp	Arg	10.88 ± 0.77	10.26 ± 0.61	10.09 ± 0.51
Asp	Agh	12.00 ± 0.94	10.08 ± 0.50	9.23 ± 1.19
Glu	Agp	11.13 ± 0.35	10.46 ± 0.72	9.76 ± 0.56
Glu	Agb	11.30 ± 0.48	10.60 ± 0.52	9.53 ± 0.32
Glu	Arg	11.98 ± 1.52	10.14 ± 1.08	10.08 ± 0.72
Glu	Agh	11.30 ± 0.48	9.94 ± 0.78	9.05 ± 0.79
Aad	Agp	10.63 ± 0.52	10.31 ± 0.58	9.47 ± 0.62
Aad	Agb	11.00 ± 0.00	10.80 ± 0.42	8.92 ± 1.35
Aad	Arg	11.00 ± 0.82	10.04 ± 1.48	9.20 ± 1.43
Aad	Agh	11.10 ± 0.57	10.18 ± 0.81	9.15 ± 0.97

[a]The average $^{3}J_{NH\alpha}$ coupling constant values for the strand residues were calculated without including values for Gly, which was in the β-turn.

Table S3. The ^{1}H Chemical Shift Assignments for Peptide HPDAspAgp.

Residue	HN	Hα	Hβ	Others
Ac		2.034		
Arg1	8.324	4.271	1.804, 1.728	Hγ: 1.619; Hδ: 3.193; NHt: 7.219
Asp2[a]	8.497	4.689	2.716, 2.568	
Val3[b]	8.198	4.242	2.121	Hγ: 0.915
Thr4	8.324	4.636	4.000	Hγ: 1.078
Val5[c]	8.431	4.521	2.011	Hγ: 0.936
^{D}Pro6		4.408	2.335, 1.978	Hγ: 2.054; Hδ: 3.893, 3.801
Gly7[d]	8.412	3.988, 3.812		
Orn8[e]	8.126	4.511	1.883, 1.817	Hγ: 1.744, 1.687; Hδ: 3.014; NHt: 7.634
Agp9[f]	8.771	4.758	3.677, 3.444	HNt: 7.385
Ile10[g]	8.534	4.211	1.852	Hγ: 1.405, 1.168, 0.883(Me); Hδ: 0.820
Leu11	8.452	4.445	1.630	Hγ: 1.578; Hδ: 0.922, 0.863
Gln12[h]	8.505	4.312	2.104, 1.973	Hγ: 2.358; HNt: 7.525, 6.899
NH_2	7.662, 7.146			

[a]The assignments for the minor Asp2 spin system are 8.508(HN), 4.649(Hα), 2.723, 2.580(Hβ). [b]The assignments for the minor Val3 spin system are 8.118(HN), 4.186(Hα), 2.171(Hβ), 0.921(Hγ). [c]The assignments for the minor Val5 spin system are 8.279(HN), 4.095 (Hα), 2.049(Hβ), 0.897, 0.798(Hγ); [d]The assignments for the minor Gly7 spin system are 8.676(HN), 4.016, 3.953(Hα). [e]The assignments for the minor Orn8 spin system are 8.477(HN), 4.375(Hα), 1.872, 1.752(Hβ), 1.631, 1.590(Hγ), 3.014(Hδ). [f]The assignments for the minor Agp9 spin system are 8.688(HN), 4.650(Hα), 3.637, 3.465(Hβ). [g]The assignments for the minor Ile10 spin system are 8.431(HN), 4.143(Hα), 1.835(Hβ), 1.167(Hγ), 0.870(Hδ). [h]The assignments for the minor Gln12 spin system are 8.435(HN), 4.295 (Hα), 2.099(Hβ), 2.364(Hr), 7.551, 6.896(HNt).

Table S4. The ^{1}H Chemical Shift Assignments for Peptide HPDGluAgp.

Residue	HN	Hα	Hβ	Others
Ac		2.032		
Arg1[a]	8.271	4.307	1.816, 1.733	Hγ: 1.623; Hδ: 3.200; HNt: 7.210
Glu2	8.523	4.477	1.919	Hγ: 2.247, 2.179
Val3[b]	8.452	4.288	2.080	Hγ: 0.917
Thr4[c]	8.310	4.715	4.012	Hγ: 1.074
Val5[d]	8.456	4.555	2.003	Hγ: 0.927
^{D}Pro6		4.402	2.337, 1.974	Hγ: 2.061; Hδ: 3.878, 3.812
Gly7[e]	8.410	3.980, 3.816		
Orn8[f]	8.109	4.521	1.875, 1.822	Hγ: 1.742, 1.686; Hδ: 3.015; HNt: 7.623
Agp9	8.762	4.808	3.626, 3.457	HNt: 7.361
Ile10[g]	8.593	4.266	1.843	Hγ: 1.392, 1.142, 0.873(Me); Hδ: 0.811
Leu11	8.431	4.466	1.620	Hγ: 1.582; Hδ: 0.907, 0.859
Gln12[h]	8.515	4.321	2.091, 1.971	Hγ: 2.341; HNt: 7.484, 6.872
NH_2	7.655, 7.127			

[a]The assignments for the minor Arg1 spin system are 8.456(HN), 4.373(Hα), 1.876, 1.756(Hβ), 1.611(Hγ), 3.011(Hδ). [b]The assignments for the minor Val3 spin system are 8.267(HN), 4.161(Hα), 2.090(Hβ), 0.927(Hγ). [c]The assignments for the minor Thr4 spin system are 8.212(HN), 4.290(Hα), 4.100(Hβ), 1.158(Hγ). [d]The assignments for the minor Val5 spin system are 8.608(HN), 4.567Hα), 1.993(Hβ), 0.932(Hγ). [e]The assignments for the minor Gly7 spin system are 8.701(HN), 4.007, 3.965(Hα). [f]The assignments for the minor Orn8 spin system are 8.025(HN), 4.533(Hα), 1.864(Hβ), 1.704(Hγ), 3.019(Hδ). [g]The assignments for the minor Ile10 spin system are 8.393(HN), 4.149(Hα), 1.836(Hβ), 1.463, 1.167(Hγ), 0.897(Hδ). [h]The assignments for the minor Gln12 spin system are 8.399(HN), 4.300(Hα), 2.100, 1.977(Hβ), 2.363(Hγ), 7.531, 6.872(HNt).

Table S5. The ^{1}H Chemical Shift Assignments for Peptide HPDAadAgp.

Residue	HN	Hα	Hβ	Others
Ac		2.021		
Arg1	8.279	4.310	1.807, 1.736	Hγ: 1.620; Hδ: 3.198; NHt: 7.216
Aad2	8.448	4.462	1.708	Hγ: 1.492; Hδ: 2.182
Val3[a]	8.470	4.283	2.060	Hγ: 0.915
Thr4[b]	8.328	4.695	4.020	Hγ: 1.077
Val5[c]	8.476	4.553	1.992	Hγ: 0.933
^{D}Pro6		4.406	2.337, 1.981	Hγ: 2.061; Hδ: 3.875, 3.813
Gly7[d]	8.435	3.975, 3.827		
Orn8[e]	8.127	4.521	1.881, 1.820	Hγ: 1.745, 1.689; Hδ: 3.016; NHt: 7.639
Agp9[f]	8.787	4.837	3.602, 3.467	NHt: 7.368
Ile10[g]	8.595	4.270	1.845	Hγ: 1.389, 1.138, 0.876(Me); Hδ: 0.817
Leu11	8.455	4.468	1.615	Hγ: 1.586; Hδ: 0.857
Gln12[h]	8.552	4.322	2.098, 1.967	Hγ: 2.348; HNt: 7.515, 6.893
NH_2	7.679, 7.150			

[a]The assignments for the minor Val3 spin system are 8.380(HN), 4.110(Hα), 2.040(Hβ), 0.898, 802(Hγ). [b]The assignments for the minor Thr4 spin system are 8.243(HN), 4.278(Hα), 4.098(Hβ), 1.152(Hγ). [c]The assignments for the minor Val5 spin system are 8.294(HN), 4.156 (Hα), 2.068(Hβ), 0.924(Hγ). [d]The assignments for the minor Gly7 spin system are 8.689(HN), 4.010, 3.962(Hα). [e]The assignments for the minor Orn8 spin system are 8.478(HN), 4.376 (Hα), 1.880(Hβ), 1.755, 1.603(Hγ), 3.005(Hδ). [f]The assignments for the minor Agp9 spin system are 8.683(HN), 4.651(Hα), 3.639, 3.469(Hβ), 7.297(NHt). [g]The assignments for the minor Ile10 spin system are 8.420(HN), 4.145(Hα), 1.834(Hβ), 1.171(Hγ), 0.900(Hδ). [h]The assignments for the minor Gln12 spin system are 8.428(HN), 4.297(Hα), 2.103, 1.979(Hβ), 2.366(Hγ), 7.554, 6.892(HNt).

Table S6. The ^{1}H Chemical Shift Assignments for Peptide HPDAspAgb.

Residue	HN	Hα	Hβ	Others
Ac		2.040		
Arg1	8.298	4.284	1.817	Hγ: 1.729, 1.624; Hδ: 3.195; NHt: 7.209
Asp2[a]	8.472	4.700	2.695, 2.576	
Val3[b]	8.145	4.255	2.131	Hγ: 0.917
Thr4	8.312	4.609	4.026	Hγ: 1.087
Val5[c]	8.448	4.514	2.027	Hγ: 0.940
^{D}Pro6		4.406	2.333, 1.963	Hγ: 2.052, 1.985; Hδ: 3.892, 3.795
Gly7[d]	8.336	3.980, 3.801		
Orn8	8.074	4.486	1.882	Hγ: 1.806, 1.716; Hδ: 3.021; NHt: 7.620
Agb9[e]	8.636	4.533	2.030, 1.952	Hγ: 3.280, 3.226; NHt: 7.308
Ile10[f]	8.445	4.195	1.850	Hγ: 1.427, 1.178, 0.883(Me); Hδ: 0.825
Leu11	8.408	4.434	1.636	Hγ: 1.581; Hδ: 0.915, 0.857
Gln12[g]	8.426	4.309	2.100, 1.973	Hγ: 2.352; HNt: 7.516, 6.870
NH_2	7.629, 7.115			

[a]The assignments for the minor Asp2 spin system are 8.484(HN), 4.656 (Hα), 2.734, 2.595(Hβ. [b]The assignments for the minor Val3 spin system are 8.083(HN), 4.186(Hα), 2.166(Hβ), 0.920(Hγ). [c]The assignments for the minor Val5 spin system are 8.240(HN), 4.095(Hα), 2.046(Hβ), 0.894, 0.798(Hγ). [d]The assignments for the minor Gly7 spin system are 8.632(HN), 4.007, 3.944(Hα). [e]The assignments for the minor Agb9 spin system are 8.591(HN), 4.431 (Hα), 2.045, 1.952(Hβ), 3.273, 3.231(Hγ), 7.228(NHt). [f]The assignments for the minor Ile10 spin system are 8.346(HN), 4.135(Hα), 1.834(Hβ), 1.179(Hγ), 0.889(Hδ). [g]The assignments for the minor Gln12 spin system are 8.364(HN), 4.299(Hα), 2.100, 1.973(Hβ), 2.352(Hγ).

Table S7. The ^{1}H Chemical Shift Assignments for Peptide HPDGluAgb.

Residue	HN	Hα	Hβ	Others
Ac		2.032		
Arg1	8.265	4.321	1.818, 1.729	Hγ: 1.622; Hδ: 3.199; NHt: 7.205
Glu2	8518	4.550	1.925	Hγ: 2.253, 2.161
Val3[a]	8.516	4.324	2.071	Hγ: 0.899
Thr4[b]	8.398	4.757	4.008	Hγ: 1.055
Val5[c]	8.637	4.561	1.989	Hγ: 0.929
^{D}Pro6		4.389	2.346, 1.974	Hγ: 2.096, 2.042; Hδ: 3.868, 3.830
Gly7[d]	8.369	3.989, 3.782		
Orn8[e]	8.035	4.554	1.870, 1.800	Hγ: 1.707; Hδ: 3.017; NHt: 7.619
Agb9[f]	8.667	4.658	1.940	Hδ: 3.275, 3.196; NHt: 7.231
Ile10[g]	8.668	4.306	1.848	Hγ: 1.389, 1.144, 0.870(Me); Hδ: 0.809
Leu11	8.413	4.477	1.617	Hγ: 1.580; Hδ: 0.897, 0.845
Gln12[h]	8.532	4.327	2.091, 1.954	Hγ: 2.330; HNt: 7.469, 6.871
NH_2	7.671, 7.132			

[a]The assignments for the minor Val3 spin system are 8.364(HN), 4.162(Hα), 2.093(Hβ), 0.928(Hγ). [b]The assignments for the minor Thr4 spin system are 8.219(HN), 4.290 (Hα), 4.102(Hβ), 1.158(Hγ). [c]The assignments for the minor Val5 spin system are 8.351(HN), 4.103(Hα), 2.037(Hβ), 0.894, 0.799(Hγ). [d]The assignments for the minor Gly7 spin system are 8.682(HN), 4.003, 3.949(Hα). [e]The assignments for the minor Orn8 spin system are 8.423(HN), 4.336(Hα), 1.866(Hβ), 1.755(Hγ), 3.016(Hδ); [f]The assignments for the minor Agb9 spin system are 8.588(HN), 4.431(Hα), 2.048, 1.950(Hβ), 3.272, 3.230(Hγ). [g]The assignments for the minor Ile10 spin system are 8.344(HN), 4.134(Hα), 1.836(Hβ), 1.181(Hγ), 0.889(Hδ). [h]The assignments for the minor Gln12 spin system are 8.364(HN), 4.301(Hα), 2.100, 1.976(Hβ), 2.360(Hr), 7.532, 6.868(NHt).

Table S8. The ^{1}H Chemical Shift Assignments for Peptide HPDAadAgb.

Residue	HN	Hα	Hβ	Others
Ac		2.023		
Arg1	8.252	4.348	1.811, 1.733	Hγ: 1.616; Hδ: 3.197; NHt: 7.198
Aad2	8.420	4.629	1.685	Hγ: 1.617; Hδ: 2.173, 2.116
Val3[a]	8.568	4.360	2.043	Hγ: 0.897, 0.881
Thr4[b]	8.447	4.794	4.016	Hγ: 1.053
Val5[c]	8.712	4.570	1.994	Hγ: 0.928
^{D}Pro6		4.383	2.351, 1.971	Hγ: 2.107, 2.046; Hδ: 3.851
Gly7[d]	8.370	3.988, 3.775		
Orn8	8.019	4.584	1.867, 1.798	Hγ: 1.706; Hδ: 3.018; NHt: 7.624
Agb9[e]	8.674	4.720	1.955, 1.871	Hγ: 3.289, 3.193; NHt: 7.280
Ile10	8.753	4.360	1.848	Hγ: 1.378, 1.133, 0.869(Me); Hδ: 0.804
Leu11	8.418	4.488	1.586	Hγ: 1.453; Hδ: 0.884, 0.840
Gln12[f]	8.585	4.332	2.087, 1.946	Hγ: 2.324; HNt: 7.454, 6.869
NH_2	7.688, 7.137			

[a]The assignments for the minor Val3 spin system are 8.264(HN), 4.161(Hα), 2.072(Hβ), 0.923(Hγ); [b]The assignments for the minor Thr4 spin system are 8.223(HN), 4.282 (Hα), 4.105(Hβ), 1.154(Hγ); [c]The assignments for the minor Val5 spin system are 8.340(HN), 4.126(Hα), 2.037, 1.879(Hβ), 0.893, 0.803(Hγ); [d]The assignments for the minor Gly7 spin system are 8.647(HN), 3.999, 3.950(Hα). [e]The assignments for the minor Agb9 spin system are 8.421(HN), 4.387(Hα), 1.879, 1.770(Hβ); [f]The assignments for the minor Gln12 spin system are 8.364(HN), 4.301(Hα), 2.104, 1.977(Hβ), 2.359 (Hr).

Table S9. The ^{1}H Chemical Shift Assignments for Peptide HPDAspArg.

Residue	HN	Hα	Hβ	Others
Ac		2.038		
Arg1	8.292	4.285	1.817, 1.730	Hγ: 1.625; Hδ: 3.199; HNt: 7.209
Asp2[a]	8.450	4.696	2.695, 2.582	
Val3[b]	8.152	4.244	2.130	Hγ: 0.921
Thr4	8.270	4.641	3.991	Hγ: 1.077
Val5[c]	8.457	4.519	2.032	Hγ: 0.941
^{D}Pro6		4.402	2.334, 1.967	Hγ: 2.054, 1.987; Hδ: 3.895, 3.796
Gly7[d]	8.346	3.796, 3.983		
Orn8	8.025	4.490	1.872, 1.807	Hγ: 1.731, 1.684; Hδ: 3.019; NHt: 7.615
Arg9	8.509	4.441	1.792, 1.711	Hγ: 1.632, 1.541; Hδ: 3.171; NHt: 7.261
Ile10[e]	8.422	4.187	1.850	Hγ: 1.435, 1.182, 0.881(Me); Hδ: 0.816
Leu11	8.388	4.426	1.637	Hγ: 1.584; Hδ: 0.919, 0.854
Gln12[f]	8.393	4.306	2.105, 1.976	Hγ: 2.354; HNt: 6.867, 7.518
NH_2	7.110, 7.629			

[a]The assignments for the minor Asp2 spin system are 8.480(HN), 4.651(Hα), 2.746, 2.607(Hβ); [b]Val3 spin system are 8.076(HN), 4.189(Hα), 2.167(Hβ), 0.921(Hγ); [c]Val5 spin system are 8.326(HN), 4.139(Hα), 1.832(Hβ), 0.883(Hγ); [d]Gly7 spin system are 8.620(HN), 4.005, 3.933(Hα); [e]Ile10 spin system are 8.235(HN), 4.100 (Hα), 2.045(Hβ), 0.894(Hγ), 0.797(Hδ); [f]Gln12 spin system are 8.338(HN), 4.303 (Hα), 1.976(Hβ), 2.359(Hγ).

Table S10. The ^{1}H Chemical Shift Assignments for Peptide HPDGluArg.

Residue	HN	Hα	Hβ	Others
Ac		2.029		
Arg1[a]	8.259	4.316	1.818, 1.733	Hγ: 1.625; Hδ: 3.199; HNt: 7.203
Glu2[b]	8.504	4.530	1.927	Hγ: 2.173, 2.067
Val3[c]	8.503	4.319	2.249	Hγ: 0.908
Thr4[d]	8.289	4.777	3.962	Hγ: 1.047
Val5	8.601	4.566	2.004	Hγ: 0.931
^{D}Pro6		4.390	1.972, 2.346	Hγ: 2.046, 2.094; Hδ: 3.823, 3.884
Gly7[e]	8.401	3.777, 3.992		
Orn8	8.006	4.537	1.860, 1.810	Hγ: 1.722, 1.680; Hδ: 3.015; NHt: 7.618
Arg9	8.521	4.537	1.722	Hγ: 1.525, 1.614; Hδ: 3.151; NHt: 7.258
Ile10[f]	8.613	4.275	1.849	Hγ: 1.409, 1.156, 0.866(Me); Hδ: 0.801
Leu11	8.400	4.472	1.625	Hγ: 1.579; Hδ: 0.902, 0.844
Gln12[g]	8.489	4.293	1.965, 2.094	Hγ: 2.336; HNt: 6.866, 7.469
NH_2	7.121, 7.658			

[a]The assignments for the minor Arg1 spin system are 8.421(HN), 4.316 (Hα), 1.801, 1.738(Hβ), 1.631, 1.582(Hγ); [b]Glu2 spin system are 8.339(HN), 4.300(Hα), 1.979(Hβ), 2.359, 2.103(Hγ); [c]Val3 spin system are 8.260(HN), 4.162(Hα), 2.090(Hβ), 0.927(Hγ); [d]Thr4 spin system are 8.221(HN), 4.294(Hα), 4.102(Hβ), 1.157(Hγ); [e]Gly7 spin system are 8.666(HN), 4.000, 3.935(Hα); [f]Ile10 spin system are 8.333(HN),4.120(Hα), 1.832(Hβ), 0.892(Hγ), 0.797(Hδ); [g]Gln12 spin system are 8.542(HN),4.337(Hα), 2.037, 1.940(Hβ), 2.250(Hγ).

Table S11. The ^{1}H Chemical Shift Assignments for Peptide HPDAadArg.

Residue	HN	Hα	Hβ	Others
Ac		2.023		
Arg1[b]	8.248	4.323	1.807, 1.733	Hγ: 1.619 ; Hδ: 3.197; NHt: 7.206
Aad2	8.409	4.528	2.146	Hγ: 1.484; Hδ: 2.195
Val3[c]	8.492	4.303	2.054	Hγ: 0.903
Thr4[a]	8.287	4.750	3.973	Hγ: 1.048
Val5	8.611	4.562	1.994	Hγ: 0.930
^{D}Pro6		4.391	2.343, 1.973	Hγ: 2.093, 2.045; Hδ: 3.874, 3.822
Gly7[d]	8.400	3.992, 3.795		
Orn8	8.010	4.537	1.862, 1.809	Hγ: 1.727, 1.680; Hδ: 3.015; NHt: 7.621
Arg9	8.528	4.558	1.730, 1.619	Hγ: 1.543; Hδ:3.151; NHt: 7.245
Ile10[e]	8.600	4.291	1.841	Hγ: 1.408, 1.153, 0.867(Me); Hδ: 0.803
Leu11	8.408	4.465	1.686	Hγ: 1.597; Hδ: 0.898, 0.844
Gln12[f]	8.505	4.320	2.093, 1.961	Hγ: 2.337; HNt: 7.473, 6.869
NH_2	7.658, 7.125			

[a]Signal for Thr4 Ha not observed. The assignments for the minor Thr4 spin system are 8.220(HN), 4.283(Hα), 4.099(Hβ), 1.150(Hγ). [b]The assignments for the minor Arg1 spin system are 8.370(HN), 4.345(Hα), 1.859, 1.742(Hβ), 1.586(Hγ); [c]Val3 spin system are 8.259(HN), 4.157(Hα), 2.069(Hβ), 0.922(Hγ); [d]Gly7 spin system are 8.635(HN), 3.999, 3.935(Hα); [e]Ile10 spin system are 8.328(HN), 4.124(Hα), 1.830(Hβ), 0.889(Hγ), 0.799(Hδ); [f]Gln12 spin system are 8.339(HN), 4.301(Hα), 2.103, 1.976(Hβ), 2.359(Hγ).

Table S12. The ^{1}H Chemical Shift Assignments for Peptide HPDAspAgh.

Residue	HN	Hα	Hβ	Others
Ac		2.040		
Arg1	8.298	4.284	1.819, 1.729	Hγ: 1.628; Hδ: 3.196; NHt: 7.213
Asp2[a]	8.460	4.684	2.691, 2.585	
Val3[b]	8.148	4.239	2.134	Hγ: 0.925
Thr4	8.249	4.631	3.990	Hγ: 1.082
Val5	8.452	4.508	2.028	Hγ: 0.944
^{D}Pro6		4.406	2.333, 1.962	Hγ: 2.051, 1.983; Hδ: 3.898, 3.791
Gly7[c]	8.347	3.985, 3.793		
Orn8	8.015	4.481	1.874, 1.804	Hγ: 1.728, 1.687; Hδ: 3.019; NHt: 7.615
Agh9	8.457	4.392	1.746, 1.696	Hγ: 1.575; Hδ: 1.394,1.324; Hε: 3.143; NHt: 7.174
Ile10[d]	8.371	4.174	1.848	Hγ: 1.438, 1.184, 0.880(Me); Hδ: 0.817
Leu11[e]	8.366	4.410	1.638	Hγ: 1.582; Hδ: 0.920, 0.857
Gln12[f]	8.380	4.305	1.970	Hγ: 2.355, 2.106; HNt: 7.528, 6.870
NH_2	7.633, 7.105			

[a]The assignments for the minor Asp2 spin system are 8.483(HN), 4.646(Hα), 2.740, 2.600(Hβ); [b]The assignments for the minor Val3 spin system are 8.078(HN), 4.189(Hα), 2.165(Hβ), 0.921(Hγ); [c]The assignments for the minor Gly7 spin system are 8.625(HN), 3.998, 3.937(Hα); [d]The assignments for the minor Ile10 spin system are 8.291 (HN), 4.132(Hα), 1.177(Hγ), 0.885(Hδ); [e]The assignments for the minor Leu11 spin system are 8.239 (HN), 4.094(Hα), 2.045(Hβ), 0.893(Hγ), 0.798(Hδ). [f]The assignments for the minor Gln12 spin system are 8.330(HN), 4.299(Hα), 1.974(Hβ), 2.361, 2.104(Hγ).

Table S13. The ^{1}H Chemical Shift Assignments for Peptide HPDGluAgh.

Residue	HN	Hα	Hβ	Others
Ac		2.029		
Arg1	8.263	4.313	1.817, 1.732	Hγ: 1.621; Hδ: 3.198; NHt: 7.209
Glu2[a]	8.519	4.509	1.990, 1.929	Hγ: 2.257, 2.184
Val3[b]	8.493	4.294	2.078	Hγ: 0.910
Thr4[c]	8.259	4.749	3.968	Hγ: 1.056
Val5[d]	8.605	4.555	1.997	Hγ: 0.930
^{D}Pro6		4.391	2.343, 1.971	Hγ: 2.085, 2.032; Hδ: 3.877, 3.819
Gly7[e]	8.390	3.985, 3.780		
Orn8	7.999	4.532	1.867, 1.804	Hγ: 1.701; Hδ: 3.017; NHt: 7.616
Agh9[f]	8.459	4.506	1.692	Hγ: 1.542; Hδ: 1.382, 1.305; Hε: 3.122; NHt: 7.151
Ile10	8.566	4.254	1.839	Hγ: 1.408, 1.159, 0.866(Me); Hδ: 0.802
Leu11	8.379	4.473	1.624	Hγ: 1.574; Hδ: 0.904, 0.846
Gln12	8.486	4.328	2.108, 1.965	Hγ: 2.337; HNt: 7.478, 6.869
NH_2	7.658, 7.125			

[a]The assignments for the minor Glu2 spin system are 8.546(HN), 4.337(Hα), 2.032, 1.939(Hβ), 2.263, 2.183(Hγ). [b]The assignments for the minor Val3 spin system are 8.263(HN), 4.161(Hα), 2.089(Hβ), 0.934(Hγ); [c]Thr4 spin system are 8.225(HN), 4.293(Hα), 4.102(Hβ), 1.155(Hγ); [d]The assignments for the minor Val5 spin system are 8.342(HN), 4.105(Hα), 2.033(Hβ), 0.898, 0.797(Hγ); [e]Gly7 spin system are 8.668(HN), 3.994, 3.938(Hα). [f]The assignments for the minor Agh9 spin system are 8.367(HN), 4.351(Hα), 1.864(Hβ), 1.740(Hγ), 1.387, 1.345(Hδ), 3.011(Hε), 7.124(NHt).

Table S14. The ^{1}H Chemical Shift Assignments for Peptide HPDAadAgh.

Residue	HN	Hα	Hβ	Others
Ac		2.021		
Arg1[a]	8.252	4.327	1.808, 1.735	Hγ: 1.619; Hδ: 3.197; NHt: 7.203
Aad2	8.415	4.533	1.692	Hγ: 1.619, 1.492; Hδ:2.205, 2.150
Val3[b]	8.498	4.304	2.064	Hγ: 0.905
Thr4[c]	8.259	4.738	3.971	Hγ: 1.056
Val5[d]	8.621	4.557	1.995	Hγ: 0.930
^{D}Pro6		4.390	2.343, 1.971	Hγ: 2.091, 2.045; Hδ: 3.871, 3.820
Gly7[e]	8.395	3.984, 3.790		
Orn8	8.001	4.539	1.868, 1.804	Hγ: 1.702; Hδ: 3.017; NHt: 7.619
Agh9	8.465	4.535	1.697	Hγ: 1.658, 1.541; Hδ: 1.391, 1.316; Hε: 3.125; NHt: 7.156
Ile10[f]	8.591	4.273	1.839	Hγ: 1.405, 1.156, 0.868(Me); Hδ: 0.802
Leu11	8.387	4.474	1.629	Hγ: 1.590; Hδ: 0.896, 0.846
Gln12[g]	8.507	4.326	2.098, 1.957	Hγ: 2.337; HNt: 7.473, 6.870
NH_2	7.662, 7.128			

[a]The assignments for the minor Arg1 spin system are 8.366(HN), 4.352(Hα), 1.865, 1.737(Hβ), 1.590(Hγ), 3.013(Hδ); [b]The assignments for the minor Val3 spin system are 8.334(HN), 4.111(Hα), 2.034(Hβ), 0.895, 0.800(Hγ); [c]The assignments for the minor Thr4 spin system are 8.225(HN), 4.286(Hα), 4.101(Hβ), 1.152(Hγ); [d]The assignments for the minor Val5 spin system are 8.262(HN), 4.159(Hα), 2.073(Hβ), 0.931(Hγ); [e]The assignments for the minor Gly7 spin system are 8.639(HN), 3.995, 3.940(Hα); [f]The assignments for the minor Ile10 spin system are 8.288 (HN), 4.134(Hα), 1.830(Hβ), 1.181(Hγ), 0.885(Hδ); [g]The assignments for the minor Gln12 spin system are 8.330(HN), 4.300(Hα), 2.103, 1.978(Hβ), 2.360(Hγ), 7.535, 6.867(HNt).

Table S15. The ^{1}H Chemical Shift Assignments for Peptide HPDUAspAgp.

Residue	HN	Hα	Hβ	Others
Ac		2.039		
Arg1	8.327	4.263	1.810, 1.733	Hγ: 1.621; Hδ: 3.194; NHt: 7.222
Asp2[a]	8.499	4.640	2.728, 2.601	
Val3[b]	8.072	4.190	2.149	Hγ: 0.922
Thr4[c]	8.334	4.312	4.128	Hγ: 1.178
Val5[d]	8.218	4.426	2.071	Hγ: 0.972
^{L}Pro6		4.404	2.311, 1.930	Hγ: 2.059, 1.989; Hδ: 3.892, 3.701
Gly7[e]	8.497	3.959		
Orn8[f]	8.292	4.386	1.882	Hγ: 1.765, 1.682; Hδ: 3.005; NHt: 7.638
Agp9[g]	8.645	4.660	3.636, 3.480	HNt: 7.280
Ile10[h]	8.406	4.146	1.838	Hγ: 1.448, 1.168, 0.895(Me); Hδ: 0.853
Leu11	8.448	4.379	1.638	Hγ: 1.589; Hδ: 0.933, 0.875
Gln12[i]	8.429	4.293	2.103, 1.980	Hγ: 2.367; HNt: 7.550, 6.889
NH_2	7.628, 7.133			

[a]The assignments for the minor Asp2 spin system are 8.512(HN), 4.665(Hα), 2.750, 2.616(Hβ). [b]The assignments for the minor Val3 spin system are 8.124(HN), 4.240(Hα), 2.192(Hβ), 0.926(Hγ). [c]The assignments for the minor Thr4 spin system are 8.268(HN), 4.314 (Hα), 4.164(Hβ), 1.197(Hγ). [d]The assignments for the minor Val5 spin system are 8.004(HN), 4.273(Hα), 1.990(Hβ), 0.902(Hγ). [e]The assignments for the minor Gly7 spin system are 8.576(HN), 3.970, 3.917(Hα). [f]The assignments for the minor Orn8 spin system are 8.127(HN), 4.367(Hα),1.864(Hβ), 1.778, 1.678(Hγ), 3.010(Hδ) and 8.510(HN), 4.364(Hα),1.875(Hβ), 1.761, 1.696(Hγ), 3.013(Hδ). [g]The assignments for the minor Agp9 spin system are 8.670(HN), 4.652(Hα), 3.640, 3.486(Hβ). [h]The assignments for the minor Ile10 spin system are 8.204(HN), 4.136(Hα), 1.847(Hβ), 0.877(Hγ). [i]The assignments for the minor Gln12 spin system are 8.376(HN), 4.295(Hα), 2.106, 1.977(Hβ), 2.365(Hγ).

Table S16. The ^{1}H Chemical Shift Assignments for Peptide HPDUGluAgp.

Residue	HN	Hα	Hβ	Others
Ac		2.039		
Arg1	8.281	4.271	1.819, 1.735	Hγ: 1.631; Hδ: 3.203; HNt: 7.215
Glu2	8.548	4.331	2.032, 1.939	Hγ: 2.277, 2.237
Val3[a]	8.258	4.164	2.081	Hγ: 0.931
Thr4[b]	8.271	4.354	4.108	Hγ: 1.162
Val5[c]	8.281	4.445	2.056	Hγ: 0.972, 0.937
^{L}Pro6		4.399	2.310, 1.931	Hγ: 2.062, 1.990; Hδ: 3.884, 3.705
Gly7[d]	8.476	3.959		
Orn8[e]	8.271	4.388	1.882	Hγ: 1.778, 1.681; Hδ: 3.006; HNt: 7.625
Agp9[f]	8.630	4.659	3.639, 3.480	HNt: 7.269
Ile10[g]	8.383	4.152	1.840	Hγ: 1.451, 1.167, 0.894(Me); Hδ: 0.852
Leu11	8.425	4.383	1.641	Hγ: 1.592; Hδ: 0.931, 0.876
Gln12[h]	8.403	4.298	2.101, 1.977	Hγ: 2.365; HNt: 7.528, 6.873
NH_2	7.611, 7.114			

[a]The assignments for the minor Val3 spin system are 8.179(HN), 4.141(Hα), 1.848(Hβ), 0.890(Hγ). [b]The assignments for the minor Thr4 spin system are 8.194(HN), 4.355 (Hα), 4.154(Hβ), 1.181(Hγ). [c]The assignments for the minor Val5 spin system are 8.011(HN), 4.270(Hα), 1.987(Hβ), 0.900(Hγ). [d]The assignments for the minor Gly7 spin system are 8.555(HN), 3.974, 3.907(Hα). [e]The assignments for the minor Orn8 spin system are 8.480(HN), 4.366(Hα), 1.871(Hβ), 1.759(Hγ), 3.009(Hδ). [f]The assignments for the minor Agp9 spin system are 8.663(HN), 4.651(Hα), 3.639, 3.485(Hβ), 7.295(HNt). [g]The assignments for the minor Ile10 spin system are 8.144(HN), 4.096(Hα), 1.822(Hβ), 1.481, 1.192(Hγ), 0.899(Hδ). [h]The assignments for the minor Gln12 spin system are 8.349(HN), 4.295(Hα), 2.105, 1.977(Hβ), 2.365(Hγ).

Table S17. The ^{1}H Chemical Shift Assignments for Peptide HPDUAadAgp.

Residue	HN	Hα	Hβ	Others
Ac		2.025		
Arg1	8.278	4.273	3.198	Hγ: 1.800; Hδ: 1.732, 1.624; NHt: 7.218
Aad2	8.438	4.330	1.759, 1.706	Hγ: 1.525; Hδ: 2.209
Val3	8.294	4.157	2.052	Hγ: 0.924
Thr4[a]	8.281	4.344	4.094	Hγ: 1.153
Val5[b]	8.318	4.440	2.069	Hγ: 0.971, 0.935
^{L}Pro6		4.400	2.309, 1.931	Hγ: 2.060, 1.986; Hδ: 3.885, 3.706
Gly7[c]	8.499	3.956		
Orn8[d]	8.292	4.384	1.877	Hγ: 1.769, 1.677; Hδ: 3.003; NHt: 7.639
Agp9	8.654	4.658	3.633, 3.475	NHt: 7.276
Ile10[e]	8.410	4.145	1.837	Hγ: 1.444, 1.168, 0.891(Me); Hδ: 0.850
Leu11	8.449	4.380	1.635	Hγ: 1.590; Hδ: 0.930, 0.874
Gln12[f]	8.429	4.293	2.102, 1.978	Hγ: 2.365; HNt: 7.549, 6.889
NH_2	7.627, 7.129			

[a]The assignments for the minor Thr4 spin system are 8.219(HN), 4.344(Hα), 4.151(Hβ), 1.167(Hγ). [b]The assignments for the minor Val5 spin system are 8.018(HN), 4.268(Hα), 1.985(Hβ), 0.896(Hγ). [c]The assignments for the minor Gly7 spin system are 8.585(HN), 3.980, 3.903(Hα). [d]The assignments for the minor Orn8 spin system are 8.125(HN), 4.363(Hα), 1.860(Hβ), 1.774, 1.680(Hγ), 3.004(Hδ) and 8.483(HN), 4.364(Hα), 1.867(Hβ), 1.754(Hγ), 3.003(Hδ). [e]The assignments for the minor Ile10 spin system are 8.203(HN), 4.133(Hα), 1.842(Hβ), 0.887(Hδ). [f]The assignments for the minor Gln12 spin system are 8.375(HN), 4.291(Hα), 2.104, 1.975(Hβ), 2.362(Hγ).

Table S18. The ^{1}H Chemical Shift Assignments for Peptide HPDUAspAgb.

Residue	HN	Hα	Hβ	Others
Ac		2.042		
Arg1	8.302	4.266	1.817, 1.733	Hγ: 1.623; Hδ: 3.195; NHt: 7.211
Asp2	8.475	4.638	2.723, 2.600	
Val3[a]	8.047	4.190	2.151	Hγ: 0.922
Thr4[b]	8.307	4.312	4.131	Hγ: 1.179
Val5[c]	8.182	4.427	2.093	Hγ: 0.969, 0.939
^{L}Pro6		4.401	2.309, 1.926	Hγ: 2.059, 1.989; Hδ: 3.887, 3.701
Gly7[d]	8.454	3.950		
Orn8	8.234	4.352	1.874	Hγ: 1.759, 1.684; Hδ: 3.010; NHt: 7.624
Agb9[e]	8.546	4.433	2.051, 1.963	Hγ: 3.281, 3.239; NHt: 7.227
Ile10	8.326	4.136	1.839	Hγ: 1.467, 1.182, 0.892(Me); Hδ: 0.854
Leu11	8.416	4.384	1.647	Hγ: 1.588; Hδ: 0.925, 0.864
Gln12	8.360	4.298	2.105, 1.977	Hγ: 2.361; HNt: 7.532, 6.870
NH_2	7.603, 7.113			

[a]The assignments for the minor Val3 spin system are 8.085(HN), 4.235(Hα), 2.178(Hβ), 0.926(Hγ). [b]The assignments for the minor Thr4 spin system are 8.257(HN), 4.315 (Hα), 4.173(Hβ), 1.193(Hγ). [c]The assignments for the minor Val5 spin system are 7.974(HN), 4.270(Hα), 1.990(Hβ), 0.902(Hγ). [d]The assignments for the minor Gly7 spin system are 8.540(HN), 3.956, 3.912(Hα). [e]The assignments for the minor Agb9 spin system are 8.580(HN), 4.429 (Hα), 3.275, 3.240(Hβ), 2.048, 1.963(Hγ).

Table S19. The ^{1}H Chemical Shift Assignments for Peptide HPDUGluAgb.

Residue	HN	Hα	Hβ	Others
Ac		2.039		
Arg1	8.278	4.271	1.817	Hγ: 1.733, 1.630; Hδ: 3.203; NHt: 7.215
Glu2	8.544	4.336	2.032, 1.942	Hγ: 2.286, 2.247
Val3[a]	8.258	4.164	2.079	Hγ: 0.930
Thr4[b]	8.271	4.354	4.108	Hγ: 1.162
Val5[c]	8.280	4.443	2.073	Hγ: 0.970, 0.937
^{L}Pro6		4.398	2.307, 1.926	Hγ: 2.061, 1.987; Hδ: 3.883, 3.704
Gly7[d]	8.457	3.952		
Orn8[e]	8.236	4.354	1.872	Hγ: 1.759, 1.687; Hδ: 3.010; NHt: 7.627
Agb9[f]	8.554	4.435	2.052, 1.962	Hδ: 3.282, 3.239; NHt: 7.228
Ile10	8.333	4.138	1.841	Hγ: 1.470, 1.181, 0.893(Me); Hδ: 0.853
Leu11	8.421	4.384	1.651	Hγ: 1.587; Hδ: 0.927, 0.864
Gln12	8.363	4.299	2.105, 1.978	Hγ: 2.361; HNt: 7.533, 6.871
NH_2	7.606, 7.113			

[a]The assignments for the minor Val3 spin system are 8.277(HN), 4.201(Hα), 2.501(Hβ), 0.949(Hγ). [b]The assignments for the minor Thr4 spin system are 8.212(HN), 4.354 (Hα), 4.164(Hβ), 1.177(Hγ). [c]The assignments for the minor Val5 spin system are 8.018(HN), 4.267(Hα), 1.986(Hβ), 0.900(Hγ). [d]The assignments for the minor Gly7 spin system are 8.544(HN), 3.971, 3.895(Hα). [e]The assignments for the minor Orn8 spin system are 8.418(HN), 4.333(Hα), 1.860(Hβ), 1.758(Hγ), 3.015(Hδ). [f]The assignments for the minor Agb9 spin system are 8.588(HN), 4.429(Hα), 2.047, 1.961(Hβ), 3.274, 3.240(Hγ).

Table S20. The ^{1}H Chemical Shift Assignments for Peptide HPDUAadAgb.

Residue	HN	Hα	Hβ	Others
Ac		2.028		
Arg1	8.257	4.281	1.808, 1.735	Hγ: 1.628; Hδ: 3.201; NHt: 7.210
Aad2	8.413	4.339	1.769, 1.710	Hγ: 1.533; Hδ: 2.225
Val3	8.266	4.164	2.062	Hγ: 0.926
Thr4[a]	8.253	4.350	4.101	Hγ: 1.155
Val5[b]	8.280	4.446	2.084	Hγ: 0.971, 0.937
^{L}Pro6		4.399	2.307, 1.930	Hγ: 2.062, 1.989; Hδ: 3.881, 3.706
Gly7[c]	8.459	3.953		
Orn8	8.236	4.352	1.871, 1.768	Hγ: 1.691; Hδ: 3.011; NHt: 7.631
Agb9	8.556	4.436	2.052, 1.962	Hγ: 3.284, 3.239; NHt: 7.230
Ile10	8.333	4.140	1.841	Hγ: 1.470, 1.181, 0.893(Me); Hδ: 0.854
Leu11	8.421	4.386	1.641	Hγ: 1.592; Hδ: 0.927, 0.864
Gln12[d]	8.364	4.300	2.105, 1.977	Hγ: 2.361; HNt: 7.533, 6.873
NH_2	7.606, 7.117			

[a]The assignments for the minor Thr4 spin system are 8.208(HN), 4.347 (Hα), 4.161(Hβ), 1.169(Hγ). [b]The assignments for the minor Val5 spin system are 8.004(HN), 4.270(Hα), 1.986(Hβ), 0899(Hγ). [c]The assignments for the minor Gly7 spin system are 8.555(HN), 3.974, 3.895(Hα). [e]The assignments for the minor Gln12 spin system are 7.917(HN), 4.170 (Hα),2.080, 1.922(Hβ), 2.277(Hγ).

Table S21. The [1]H Chemical Shift Assignments for Peptide HPDUAspArg.

Residue	HN	Hα	Hβ	Others
Ac		2.041		
Arg1	8.298	4.265	1.827, 1.753	Hγ: 1.623; Hδ: 3.197; HNt: 7.208
Asp2	8.476	4.646	2.729, 2.607	
Val3[a]	8.045	4.192	2.150	Hγ: 0.921
Thr4	8.304	4.316	4.128	Hγ: 1.172
Val5[b]	8.182	4.431	2.078	Hγ: 0.969, 0.939
[L]Pro6		4.391	2.365, 1.928	Hγ: 2.062, 1.990; Hδ: 3.884,3.701
Gly7[c]	8.450	3.972, 3.913		
Orn8	8.192	4.361	1.872	Hγ: 1.764, 1.683; Hδ: 3.008; NHt: 7.620
Arg9[d]	8.381	4.329	1.797, 1.744	Hγ: 1.652, 1.580; Hδ: 3.189; NHt: 7.188
Ile10	8.308	4.136	1.837	Hγ: 1.485, 1.178, 0.888 (Me); Hδ: 0.851
Leu11	8.392	4.380	1.637	Hγ: 1.575; Hδ: 0.923, 0.859
Gln12	8.336	4.300	2.103, 1.985	Hγ: 2.362; HNt: 7.530, 6.865
NH_2	7.599, 7.110			

[a]The assignments for the minor Val3 spin system are 8.074(HN), 4.231 (Hα), .172(Hβ), 0.925(Hγ); [b] Val5 spin system are 7.976(HN), 4.271(Hα), 1.987(Hβ), 0901(Hγ); [c]Gly7 spin system are 8.535(HN), 3.964, 3.898(Hα); [d]Arg9 spin system are 8.412(HN), 4.323(Hα),1.794, 1.749(Hβ), 1.651, 1.585(Hγ), 4.323(Hδ).

Table S22. The [1]H Chemical Shift Assignments for Peptide HPDUGluArg.

Residue	HN	Hα	Hβ	Others
Ac		2.038		
Arg1	8.272	4.271	1.731, 1.820	Hγ: 1.627; Hδ: 3.201; HNt: 7.210
Glu2	8.535	4.341	2.039, 1.940	Hγ: 2.254, 2.295
Val3[a]	8.252	4.166	2.080	Hγ: 0.934
Thr4[b]	8.266	4.358	4.106	Hγ: 1.162
Val5	8.274	4.443	2.056	Hγ: 0.970
[L]Pro6		4.393	2.302, 1.930	Hγ: 1.988, 2.062; Hδ: 3.703, 3.879
Gly7[c]	8.451	3.918, 3.949		
Orn8	8.192	4.361	1.750, 1.871	Hγ: 1.683; Hδ: 3.009; NHt: 7.624
Arg9[d]	8.389	4.328	1.743, 1.798	Hγ: 1.644, 1.578; Hδ: 3.190; NHt: 7.190
Ile10	8.312	4.138	1.831	Hγ: 1.497, 1.474, 0.888(Me) ; Hδ: 0.850
Leu11[e]	8.395	4.380	1.645	Hγ: 1.579; Hδ: 0.922, 0.858
Gln12	8.338	4.299	2.095, 2.119	Hγ: 2.363; HNt: 6.866, 7.530
NH_2	7.600, 7.111			

[a]The assignments for the minor [a]Val3 spin system are 8.020(HN), 4.265(Hα), 2.036(Hβ), 0.900(Hγ); [b]Thr4 spin system are 8.231(HN), 4.358(Hα), 4.162(Hβ), 1.176(Hγ); [c]Gly7 spin system are 8.539(HN), 3.979, 3.884(Hα); [d]Arg9 spin system are 8.362(HN),4.347(Hα), 1.855(Hβ), 1.751(Hγ); [e]Leu11 spin system are 8.424(HN),4.322(Hα), 1.769(Hβ), 1.593(Hγ).

Table S23. The ^{1}H Chemical Shift Assignments for Peptide HPDUAadArg.

Residue	HN	Hα	Hβ	Others
Ac		2.026		
Arg1	8.253	4.279	1.806, 1.733	Hγ: 1.626; Hδ: 3.200; HNt: 7.209
Aad2	8.408	4.349	1.764, 1.710	Hγ: 1.531; Hδ: 2.221
Val3[a]	8.263	4.165	2.056	Hγ: 0.930
Thr4[b]	8.250	4.352	4.099	Hγ: 1.156
Val5	8.275	4.446	2.084	Hγ: 0.970
^{L}Pro6		4.392	2.307, 1.929	Hγ: 2.065, 1.988; Hδ: 3.881, 3.706
Gly7[c]	8.454	3.946		
Orn8	8.194	4.356	1.871	Hγ: 1.754, 1.685; Hδ: 3.010; NHt: 7.625
Arg9[d]	8.393	4.329	1.792	Hγ: 1.739; Hδ:3.190; NHt: 7.190
Ile10	8.315	4.139	1.838	Hγ: 1.484, 1.186,0.888(Me); Hδ: 0.850
Leu11	8.400	4.382	1.642	Hγ: 1.581; Hδ: 0.922, 0.858
Gln12[e]	8.341	4.299	2.105, 1.982	Hγ: 2.362; HNt: 7.532, 6.867
NH_2	7.603, 7.111			

[a]The assignments for the minor Val3 spin system are 7.921(HN), 4.181(Hα), 1.899(Hβ), 0.814(Hγ) [b]Thr4 spin system are 8.124(HN), 4.261(Hα), 4.071(Hβ), 1.085(Hγ); [c]Gly7 spin system are 8.462(HN), 3.892, 3.797(Hα); [d]Arg9 spin system are 8.342(HN), 4.222(Hα), 1.711(Hβ), 1.658(Hγ); [e]Gln12 spin system are 8.2685(HN), 4.255(Hα), 1.662 (Hβ), 1.773 (Hγ).

Table S24. The ^{1}H Chemical Shift Assignments for Peptide HPDUAspAgh.

Residue	HN	Hα	Hβ	Others
Ac		2.039		
Arg1	8.301	4.264	1.810, 1.732	Hγ: 1.622; Hδ: 3.195; NHt: 7.211
Asp2	8.478	4.643	2.724, 2.599	
Val3[a]	8.048	4.191	2.151	Hγ: 0.921
Thr4[b]	8.308	4.312	4.129	Hγ: 1.178
Val5[c]	8.185	4.428	2.074	Hγ: 0.968, 0.939
^{L}Pro6		4.391	2.307, 1.927	Hγ: 2.060, 1.991; Hδ: 3.886, 3.699
Gly7[d]	8.454	3.943		
Orn8	8.181	4.355	1.868	Hγ: 1.733; Hδ: 3.008; NHt: 7.622
Agh9[e]	8.335	4.291	1.754, 1.721	Hγ: 1.589; Hδ: 1.393, 1.345; Hε: 3.162; NHt: 7.145
Ile10	8.274	4.132	1.834	Hγ: 1.484, 1.187 0.886(Me); Hδ: 0.849
Leu11	8.372	4.372	1.648.	Hγ: 1.580; Hδ: 0.925, 0.861
Gln12	8.328	4.293	2.108, 1.976	Hγ: 2.363; HNt: 7.535, 6.866
NH_2	7.600, 7.113			

[a]The assignments for the minor Val3 spin system are 8.076(HN), 4.227(Hα), 2.167(Hβ), 0.921(Hγ); [b]The assignments for the minor Thr4 spin system are 8.259(HN), 4.313(Hα), 4.132(Hβ), 1.187(Hγ); [c]The assignments for the minor Val5 spin system are 7.978(HN), 4.266(Hα), 1.985(Hβ), 0.898(Hγ); [d]The assignments for the minor Gly7 spin system are 8.541(HN), 3.961, 3.898(Hα); [e]The assignments for the minor Agh9 spin system are 8.364 (HN), 4.291(Hα), 1.848(Hβ), 1.737(Hγ), 1.387, 1.351(Hδ), 3.008(Hε).

Table S25. The ^{1}H Chemical Shift Assignments for Peptide HPDUGluAgh.

Residue	HN	Hα	Hβ	Others
Ac		2.040		
Arg1	8.276	4.271	1.811	Hγ: 1.734, 1.631; Hδ: 3.203; NHt: 7.217
Glu2	8.543	4.338	2.038, 1.939	Hγ: 2.289, 2.247
Val3	8.256	4.168	2.075	Hγ: 0.927
Thr4[a]	8.270	4.357	4.108	Hγ: 1.165
Val5[b]	8.278	4.445	2.060	Hγ: 0.970, 0.889
^{L}Pro6		4.393	2.308, 1.929	Hγ: 2.065, 1.988; Hδ: 3.883, 3.702
Gly7[c]	8.457	3.947		
Orn8	8.181	4.357	1.870	Hγ: 1.757, 1.696; Hδ: 3.009; NHt: 7.626
Agh9[d]	8.342	4.293	1.756, 1.722	Hγ: 1.590; Hδ: 1.394, 1.346; Hε: 3.165, 3.014; NHt: 7.150
Ile10	8.278	4.136	1.835	Hγ: 1.488, 1.190, 0.935(Me); Hδ: 0.852
Leu11	8.376	4.374	1.646	Hγ: 1.583; Hδ: 0.925, 0.861
Gln12	8.328	4.299	2.109, 1.977	Hγ: 2363; HNt: 7.536, 6.867
NH_2	7.600, 7.115			

[a]The assignments for the minor Thr4 spin system are 8.218(HN), 4.357(Hα), 4.161(Hβ), 1.177(Hγ); [b]The assignments for the minor Val5 spin system are 8.023(HN), 4.266(Hα), 1.987(Hβ), 0.903(Hγ); [c]The assignments for the minor Gly7 spin system are 8.548(HN), 3.975, 3.886(Hα). [e]The assignments for the minor Agh9 spin system are 8.377(HN), 4.288(Hα), 1.727(Hβ), 1.388, 1.351(Hδ), 3.171(Hε).

Table S26. The ^{1}H Chemical Shift Assignments for Peptide HPDUAadAgh.

Residue	HN	Hα	Hβ	Others
Ac		2.028		
Arg1	8.256	4.279	1.805, 1.733	Hγ: 1.626; Hδ: 3.200; NHt: 7.208
Aad2	8.413	4.338	1.768, 1.708	Hγ: 1.622, 1.532; Hδ: 2.222
Val3[a]	8.268	4.163	2.060	Hγ: 0.918
Thr4[b]	8.253	4.350	4.099	Hγ: 1.156
Val5[c]	8.279	4.446	2.095	Hγ: 0.889
^{L}Pro6		4.396	2.307, 1.930	Hγ: 2.063, 1.988; Hδ: 3.882, 3.704
Gly7[d]	8.456	3.947		
Orn8	8.181	4.357	1.869	Hγ: 1.757, 1.702; Hδ: 3.009; NHt: 7.622
Agh9	8.343	4.293	1.753, 1.723	Hγ: 1.593; Hδ: 1.393, 1.345; Hε: 3.165; NHt: 7.149
Ile10	8.279	4.133	1.837	Hγ: 1.487, 1.184, 0.935(Me); Hδ: 0.848
Leu11	8.377	4.377	1.649	Hγ: 1.582; Hδ: 0.926, 0.861
Gln12	8.328	4.299	2.108, 1.977	Hγ: 2.363; HNt: 7.536, 6.867
NH_2	7.602, 7.115			

[a]The assignments for the minor Val3 spin system are 8.113(HN), 4.200(Hα), 2.072(Hβ), 0.925(Hγ); [b]The assignments for the minor Thr4 spin system are 8.210(HN), 4.349(Hα), 4.159(Hβ), 1.169(Hγ); [c]The assignments for the minor Val5 spin system are 8.010(HN), 4.268(Hα), 1.985(Hβ), 0.899(Hγ); [d]The assignments for the minor Gly7 spin system are 8.556(HN), 3.976, 3.885(Hα).

Table S27. The ^{1}H Chemical Shift Assignments for Peptide HPDFAspAgp.

Residue	HN	Hα	Hβ	Others
Ac		2.073		
Cys1	8.481	5.145	3.157, 2.595	
Arg2	8.720	4.555	1.778	Hγ: 1.616; Hδ: 3.188; NHt: 7.159
Aasp3	8.557	4.943	2.676, 2.406	
Val4	8.972	4.402	2.048	Hγ: 0.862
Thr5	8.463	4.948	3.978	Hγ: 1.067
Val6	8.767	4.589	1.929	Hγ: 0.905, 0.878
^{D}Pro7		4.364	2.350, 1.968	Hγ: 2.146, 2.039; Hδ: 3.867, 3.780
Gly8	8.543	3.960, 3.834		
Orn9	8.043	4.676	1.863	Hγ: 1.735, 1.685; Hδ: 3.013; NHt: 7.642
Agp10	8.689	5.153	3.830, 3.359	HNt: 7.535
Ile11	9.187	4.418	1.820	Hγ: 1.349, 1.087, 0.846(Me); Hδ: 0.797
Leu12	8.356	4.859	1.545	Hγ: 1.483; Hδ: 0.797
Gln13	9.167	4.640	2.102, 1.922	Hγ: 2.302, 2.245; HNt: 7.324, 6.860
Cys14	9.050	5.065	3.134, 3.011	
NH_2	7.632, 7.267			

Table S28. The ^{1}H Chemical Shift Assignments for Peptide HPDFGluAgp.

Residue	HN	Hα	Hβ	Others
Ac		2.076		
Cys1	8.483	5.187	3.173, 3.002	
Arg2	8.737	4.607	1.813	Hγ: 1.651, 1.565; Hδ: 3.185; NHt: 7.154
Glu3	8.717	4.852	1.882	Hγ: 2.167, 2.058
Val4	8.994	4.545	2.055	Hγ: 0.870, 0.816
Thr5	8.536	5.102	3.950	Hγ: 1.045
Val6	8.733	4.619	1.931	Hγ: 0.918, 0.885
^{D}Pro7		4.358	2.368, 1.968	Hγ: 2.147, 2.048; Hδ: 3.877, 3.818
Gly8	8.535	3.985, 3.776		
Orn9	8.023	4.666	1.853	Hγ: 1.732, 1.673; Hδ: 3.009; NHt: 7.642
Agp10	8.844	5.129	3.574, 3.438	HNt: 7.470
Ile11	9.074	4.508	1.831	Hγ: 1.301, 1.057, 0.839(Me); Hδ: 0.792
Leu12	8.458	4.760	1.582, 1.538	Hγ: 1.479; Hδ: 0.806
Gln13	9.132	4.638	2.103, 1.901	Hγ: 2.268, 2.225; HNt: 7.397, 6.850
Cys14	9.045	5.079	3.134, 3.002	
NH_2	7.623, 7.265			

Table S29. The ^{1}H Chemical Shift Assignments for Peptide HPDFAadAgp.

Residue	HN	Hα	Hβ	Others
Ac		2.078		
Cys1	8.482	5.207	3.174, 2.597	
Arg2	8.757	4.625	1.805	Hγ: 1.647, 1.559; Hδ: 3.180; NHt: 7.148
Aad3	8.678	4.844	1.649	Hγ: 1.541, 1.431; Hδ: 2.089
Val4	8.963	4.535	2.054, 2.021	Hγ: 0.870, 0.822
Thr5	8.581	5.070	3.969	Hγ: 1.051
Val6	8.751	4.611	1.938	Hγ: 0.917, 0.886
^{D}Pro7		4.358	2.365, 1.966	Hγ: 2.148, 2.043; Hδ: 3.874, 3.807
Gly8	8.538	3.980, 3.790		
Orn9	8.005	4.678	1.854	Hγ: 1.727, 1.677; Hδ: 3.010; NHt: 7.639
Agp10	8.875	5.227		NHt: 7.418
Ile11	9.048	4.551	1.837	Hγ: 1.303, 1.049, 0.846(Me); Hδ: 0.797
Leu12	8.464	4.777	1.609	Hγ: 1.517, 1.466; Hδ: 0.813, 0.786
Gln13	9.173	4.645	2.111, 1.893	Hγ: 2.268, 2.225; HNt: 7.413, 6.850
Cys14	9.056	5.091	3.129, 3.003	
NH_2	7.618, 7.266			

Table S30. The ^{1}H Chemical Shift Assignments for Peptide HPDFAspAgb.

Residue	HN	Hα	Hβ	Others
Ac		2.071		
Cys1	8.448	5.156	3.156, 2.643	
Arg2	8.726	4.602	1.821, 1.786	Hγ: 1.651, 1.577; Hδ: 3.185; NHt: 7.140
Asp3	8.519	5.091	2.500, 2.458	
Val4	8.862	4.474	2.041	Hγ: 0.872, 0.838
Thr5	8.514	5.013	3.976	Hγ: 1.030
Val6	8.895	4.607	1.927	Hγ: 0.926, 0.897
^{D}Pro7		4.350	2.374, 1.963	Hγ: 2.148, 2.047; Hδ: 3.879, 3.831
Gly8	8.407	4.011, 3.732		
Orn9	7.947	4.693	1.870, 1.806	Hγ: 1.696; Hδ: 3.014; NHt: 7.624
Agb10	8.634	4.906	1.919	Hγ: 3.269, 3.169; NHt: 7.585
Ile11	9.114	4.457	1.839	Hγ: 1.330, 1.099, 0.850(Me); Hδ: 0.799
Leu12	8.290	4.788	1.607	Hγ: 1.513, 1.459; Hδ: 0.819, 0.791
Gln13	9.091	4.641	2.107, 1.898	Hγ: 2.285, 2.228; HNt: 7.378, 6.836
Cys14	9.001	5.049	3.127, 3.009	
NH_2	7.610, 7.243			

Table S31. The ^{1}H Chemical Shift Assignments for Peptide HPDFGluAgb.

Residue	HN	Hα	Hβ	Others
Ac		2.079		
Cys1	8.456	5.211	3.176, 2.618	
Arg2	8.733	4.622	1.829, 1.785	Hγ: 1.660, 1.551; Hδ: 3.182; NHt: 7.136
Glu3	8.701	4.951	1.888, 1.802	Hγ: 2.198, 2.027
Val4	8.965	4.535	2.033	Hγ: 0.867, 0.809
Thr5	8.661	5.024	4.001	Hγ: 1.025
Val6	8.972	4.613	1.929	Hγ: 0.927, 0.892
^{D}Pro7		4.349	2.375, 1.962	Hγ: 2.159, 2.046; Hδ: 3.889, 3.820
Gly8	8.393	4.006, 3.734		
Orn9	7.945	4.712	1.856, 1.797	Hγ: 1.694; Hδ: 3.010; NHt: 7.623
Agb10	8.691	4.977	1.914, 1.802	Hδ: 3.265, 3.147; NHt: 7.164
Ile11	9.171	4.529	1.845	Hγ: 1.318, 1.085, 0.852(Me); Hδ: 0.794
Leu12	8.400	4.699	1.614	Hγ: 1.529, 1.472; Hδ: 0.815, 0.792
Gln13	9.133	4.648	2.097, 1.893	Hγ: 2.268, 2.217; HNt: 7.361, 6.831
Cys14	9.013	5.087	3.127, 3.000	
NH_2	7.599, 7.244			

Table S32. The ^{1}H Chemical Shift Assignments for Peptide HPDFAadAgb.

Residue	HN	Hα	Hβ	Others
Ac		2.079		
Cys1	8.455	5.238	3.165, 2.612	
Arg2	8.753	4.638	1.805	Hγ: 1.657, 1.535; Hδ: 3.176; NHt: 7.125
Aad3	8.660	4.979	1.686, 1.572	Hγ: 1.486, 1.405; Hδ: 2.106, 2.028
Val4	8.959	4.522	2.024	Hγ: 0.862, 0.808
Thr5	8.690	5.009	4.012	Hγ: 1.029
Val6	9.006	4.604	1.928	Hγ: 0.926, 0.892
^{D}Pro7		4.348	2.375, 1.962	Hγ: 2.162, 2.045; Hδ: 3.889, 3.815
Gly8	8.382	4.004, 3.735		
Orn9	7.937	4.722	1.857, 1.792	Hγ: 1.694; Hδ: 3.011; NHt: 7.620
Agb10	8.697	5.002	1.943, 1.717	Hγ: 3.289, 3.155; NHt: 7.282
Ile11	9.179	4.554	1.844	Hγ: 1.322, 1.083, 0.857(Me); Hδ: 0.798
Leu12	8.419	4.667	1.656	Hγ: 1.480, 1.442; Hδ: 0.815
Gln13	9.164	4.651	2.102, 1.888	Hγ: 2.273, 2.213; HNt: 7.365, 6.832
Cys14	9.012	5.094	3.121, 2.999	
NH_2	7.589, 7.243			

Table S33. The ^{1}H Chemical Shift Assignments for Peptide HPDFAspArg.

Residue	HN	Hα	Hβ	Others
Ac		2.035		
Cys1	8.440	5.121	3.149,2.680	
Arg2	8.714	4.587	1.804, 1.653	Hγ: 1.584; Hδ: 3.188; HNt:7.140
Asp3	8.498	5.086	2.514, 2.479	
Val4	8.858	4.475	2.035	Hγ: 0.881, 0.853
Thr5	8.445	5.033	3.943	Hγ: 1.002
Val6	8.849	4.617	1.957	Hγ: 0.928,0.901
^{D}Pro7		4.352	2.372, 1.965	Hγ: 2.143, 2.047; Hδ: 3.872, 3.842
Gly8	8.450	4.007, 3.726		
Orn9	7.957	4.643	1.833	Hγ: 1.681; Hδ: 3.008; NHt: 7.618
Arg10	8.548	4.810	1.751, 1.669	Hγ: 1.565, 1.452; Hδ: 3.094; NHt: 7.404
Ile11	9.121	4.445	1.838	Hγ: 1.351, 1.105, 0.851(Me); Hδ: 0.789
Leu12	8.279	4.762	1.634	Hγ: 1.505; Hδ: 0.815
Gln13	9.056	4.646	2.109, 1.902	Hγ: 2.289, 2.238; HNt: 7.384, 6.836
Cys14	8.972	5.025	3.135, 3.003	
NH_2	7.624, 7.237			

Table S34. The ^{1}H Chemical Shift Assignments for Peptide HPDFGluArg.

Residue	HN	Hα	Hβ	Others
Ac		2.076		
Cys1	8.449	5.198	2.616, 3.171	
Arg2	8.725	4.608	1.817	Hγ: 1.557, 1.655; Hδ:3.184; HNt: 7.141
Glu3	8.693	4.967	1.859, 1.881	Hγ: 2.016, 2.174
Val4	9.019	4.530	2.037	Hγ: 0.814, 0.869
Thr5	8.490	5.064	3.954	Hγ: 1.010
Val6	8.902	4.625	1.943	Hγ: 0.891, 0.921
^{D}Pro7		4.350	1.963, 2.370	Hγ: 2.046, 2.154; Hδ: 3.827, 3.880
Gly8	8.448	4.002, 3.737		
Orn9	7.963	4.647	1.830	Hγ: 1.705, 1.602; Hδ: 3.005; NHt: 7.622
Arg10	8.541	4.852	1.644	Hγ: 1.460, 1.549; Hδ: 3.090; NHt: 7.223
Ile11	9.164	4.503	1.832	Hγ: 1.330, 1.086, 0.843(Me) ;Hδ: 0.789
Leu12	8.403	4.735	1.600	Hγ: 1.543,1.491; Hδ: 0.810
Gln13	9.124	4.648	1.906, 2.105	Hγ: 2.223, 2.271; HNt: 6.8288, 7.347
Cys14	9.003	5.085	3.005, 3.132	
NH_2	7.242, 7.605			

Table S35. The ^{1}H Chemical Shift Assignments for Peptide HPDFAadArg.

Residue	HN	Hα	Hβ	Others
Ac		2.078		
Cys1	8.448	5.230	3.169,2.617	
Arg2	8.753	4.624	1.808	Hγ: 1.652, 1.544; Hδ: 3.179; HNt: 7.130
Aad3	8.630	4.953	1.692, 1.540	Hγ: 1.438; Hδ: 2.126, 2.048
Val4	9.006	4.513	2.024	Hγ: 0.867, 0.821
Thr5	8.522	5.014	3.970	Hγ: 1.023
Val6	8.938	4.611	1.936	Hγ: 0.919, 0.890
^{D}Pro7		4.351	2.366, 1.965	Hγ: 2.151, 2.044; Hδ: 3.879, 3.814
Gly8	8.449	3.990, 3.756		
Orn9	7.960	4.667	1.828	Hγ: 1.684; Hδ: 3.001; NHt: 7.614
Arg10	8.556	4.912	1.677	Hγ: 1.563, 1.511; Hδ: 3.090; NHt: 7.227
Ile11	9.141	4.546	1.834	Hγ: 1.329, 1.085, 0.848(Me); Hδ: 0.794
Leu12	8.414	4.693	1.659	Hγ: 1.481; Hδ: 0.820, 0.772
Gln13	9.157	4.661	2.106, 1.888	Hγ: 2.277, 2.220; HNt: 7.359, 6.828
Cys14	9.002	5.094	3.123, 3.002	
NH_2	7.596, 7.541			

Table S36. The ^{1}H Chemical Shift Assignments for Peptide HPDFAspAgh.

Residue	HN	Hα	Hβ	Others
Ac		2.066		
Cys1	8.437	5.092	3.136, 2.715	
Arg2	8.695	4.602	1.834, 1.781	Hγ: 1.656, 1.570; Hδ: 3.187; NHt: 7.141
Asp3	8.490	5.070	2.469	
Val4	8.876	4.434	2.026	Hγ: 0.875
Thr5	8.450	4.989	3.932	Hγ: 1.014
Val6	8.856	4.611	1.957	Hγ: 0.925, 0.906
^{D}Pro7		4.355	2.370, 2.140	Hγ: 2.048, 1.972; Hδ: 3.868, 3.839
Gly8	8.450	4.434, 4.004		
Orn9	7.946	4.641	1.832	Hγ: 1.681; Hδ: 3.008; NHt: 7.615
Agh10	8.497	4.749	1.663	Hγ: 1.479; Hδ: 1.343, 1.231; Hε: 3.076; NHt: 7.164
Ile11	9.051	4.426	1.840	Hγ: 1.351, 1.124, 0.859(Me); Hδ: 0.797
Leu12	8.274	4.714	1.651	Hγ: 1.491; Hδ: 0.837, 0.808
Gln13	9.028	4.647	2.111, 1.896	Hγ: 2.289, 2.241; HNt: 7.399, 6.841
Cys14	8.959	4.990	3.129, 3.002	
NH_2	7.633, 7.240			

Table S37. The [1]H Chemical Shift Assignments for Peptide HPDFGluAgh.

Residue	HN	Hα	Hβ	Others
Ac		2.077		
Cys1	8.452	5.193	3.172, 2.611	
Arg2	8.727	4.613	1.812	Hγ: 1.658, 1.560; Hδ: 3.184; NHt: 7.141
Glu3	8.689	4.922	1.900, 1.853	Hγ: 2.187, 2.010
Val4	9.032	4.513	2.032	Hγ: 0.870, 0.819
Thr5	8.464	5.013	3.951	Hγ: 1.021
Val6	8.927	4.609	1.941	Hγ: 0.917, 0.890
^{D}Pro7		4.350	2.370, 1.963	Hγ: 2.149, 2.048; Hδ: 3.899, 3.823
Gly8	8.437	4.000, 3.741		
Orn9	7.941	4.665	1.832	Hγ: 1.687; Hδ: 3.011; NHt: 7.618
Agh10	8.473	4.852	1.680, 1.579	Hγ: 1.501, 1.414; Hδ: 1.336, 1.247; Hε: 3.060; NHt: 7.085
Ile11	9.158	4.484	1.828	Hγ: 1.330, 1.102, 0.846(Me); Hδ: 0.792
Leu12	8.386	4.748	1.593	Hγ: 1.541, 1.492; Hδ: 0.807
Gln13	9.129	4.648	2.103, 1.904	Hγ: 2.272, 2.224; HNt: 7.346, 6.831
Cys14	9.016	5.081	3.133, 3.006	
NH_2	7.607, 7.243			

Table S38. The [1]H Chemical Shift Assignments for Peptide HPDFAadAgh.

Residue	HN	Hα	Hβ	Others
Ac		2.079		
Cys1	8.450	5.218	3.167, 2.616	
Arg2	8.748	4.632	1.810	Hγ: 1.655, 1.549; Hδ: 3.180; NHt: 7.135
Aad3	8.641	4.930	1.691	Hγ: 1.547, 1.434; Hδ: 2.136, 2.057
Val4	9.014	4.499	2.025	Hγ: 0.870, 0.825
Thr5	8.485	4.962	3.959	Hγ: 1.033
Val6	8.952	4.602	1.941	Hγ: 0.916, 0.891
^{D}Pro7		4.353	2.365, 1.962	Hγ: 2.151, 2.045; Hδ: 3.877, 3.810
Gly8	8.439	3.992, 3.763		
Orn9	7.941	4.679	1.856, 1.815	Hγ: 1.689; Hδ: 3.013; NHt: 7.620
Agh10	8.484	4.862	1.717	Hγ: 1.548, 1.421; Hδ: 1.365, 1.282; Hε: 3.077; NHt: 7.145
Ile11	9.152	4.504	1.830	Hγ: 1.336, 1.103, 0.852(Me); Hδ: 0.798
Leu12	8.406	4.720	1.641	Hγ: 1.484; Hδ: 0.816, 0.778
Gln13	9.159	4.654	2.105, 1.898	Hγ: 2.278, 2.221; HNt: 7.350, 6.832
Cys14	9.009	5.090	3.127, 3.003	
NH_2	7.602, 7.244			

Table S39. The $^{3}J_{NH\alpha}$ Values (Hz) of the Residues in the HPDAspAgx Peptides.

	Agx			
Residue	Agp	Agb	Arg	Agh
Arg1	9.8	9.8	9.9	9.9
Asp2	9.4	9.6	9.9	9.6
Val3	10	11	11	9.8
Thr4	10	11	11	11
Val5	11	11	10	10
Gly7	9.8	9.8	8.8	10
Orn8	10	11	9.6	10
Agx9	9.9	11	9.8	9.9
Ile10	8.6	9.2	11	11
Leu11	9.4	11	10.8	9.8
Gln12	9.6	11	9.6	9.8

Table S40. The $^{3}J_{NH\alpha}$ Values (Hz) of the Residues in the HPDGluAgx Peptides.

	Agx			
Residue	Agp	Agb	Arg	Agh
Arg1	11	11	8.6	11
Glu2	9.6	11	12	9
Val3	9.2	11	10	11
Thr4	11	10	11	11
Val5	11	10	11	10
Gly7	11	11	9.8	9.9
Orn8	11	11	11	9.2
Agx9	10	10	9.0	9.6
Ile10	11	10	9.8	9.4
Leu11	9.8	11	9.8	9.4
Gln12	11	11	9.2	9.8

Table S41. The $^{3}J_{NH\alpha}$ Values (Hz) of the Residues in the HPDAadAgx Peptides.

	Agx			
Residue	Agp	Agb	Arg	Agh
Arg1	9.6	11	11	9.8
Aad2	ND[a]	11	9.6	9.6
Val3	11	11	7.6	9.8
Thr4	10	11	12	11
Val5	10	11	11	11
Gly7	9.6	11	11	10
Orn8	10	11	11	11
Agx9	9.9	10	11	11
Ile10	11	10	10	10
Leu11	ND[a]	11	9.6	10
Gln12	11	11	7.6	8.6

[a]Not determined due to signal overlap.

Table S42. The $^{3}J_{NH\alpha}$ Values (Hz) of the Residues in the HPDFAspAgx Peptides.

	Agx			
Residue	Agp	Agb	Arg	Agh
Cys1	12	11	10	11
Arg2	11	11	10	12
Asp3	10	11	9.8	11
Val4	11	11	11	13
Thr5	11	11	11	13
Val6	11	11	12	12
Gly8	9.8	11	9.2	11
Orn9	11	11	11	13
Agx10	11	11	12	11
Ile11	11	11	10	13
Leu12	11	11	11	11
Gln13	11	11	11	11
Cys14	12	12	10	12

Table S43. The $^{3}J_{NH\alpha}$ Values (Hz) of the Residues in the HPDFGluAgx Peptides.

	Agx			
Residue	Agp	Agb	Arg	Agh
Cys1	11	11	11	11
Arg2	ND[a]	11	13	11
Glu3	11	12	9.9	11
Val4	11	11	12	12
Thr5	11	11	11	11
Val6	ND[a]	12	14	11
Gly8	9.8	11	8.1	10
Orn9	11	11	13	12
Agx10	11	12	9.9	12
Ile11	11	11	12	11
Leu12	12	11	11	11
Gln13	11	11	14	11
Cys14	11	11	13	12

[a]Not determined due to signal overlap.

Table S44. The $^{3}J_{NH\alpha}$ Values (Hz) of the Residues in the HPDFAadAgx Peptides.

	Agx			
Residue	Agp	Agb	Arg	Agh
Cys1	11	12	10	11
Arg2	ND[a]	11	11	12
Aad3	10	11	12	12
Val4	11	11	10	11
Thr5	10	ND[a]	12	10
Val6	ND[a]	11	11	11
Gly8	8.6	9.8	8.6	8.8
Orn9	10	11	12	11
Agx10	11	ND[a]	11	11
Ile11	11	11	11	11
Leu12	11	11	10	11
Gln13	11	11	10	11
Cys14	11	11	11	12

[a]ND: Not determined due to signal overlap.

Table S45. The $^{3}J_{NH\alpha}$ Values (Hz) of the Residues in HPDUAspAgx Peptides.

	Agx			
Residue	Agp	Agb	Arg	Agh
Arg1	9.8	9.9	9.9	8.8
Asp2	9.4	9.6	10	9.6
Val3	9.6	8.1	10	9.4
Thr4	9.6	11	10	9.2
Val5	10	10	10	10
Gly7	13	13	14	11
Orn8	9.4	9.4	11	9.8
Agx9	9.9	8.9	9.8	7.2
Ile10	9.6	8.9	11	11
Leu11	8.9	9.6	9.4	10
Gln12	9.2	9.6	9.8	7.3

Table S46. The $^{3}J_{NH\alpha}$ Values (Hz) of the Residues in HPDUGluAgx Peptides.

	Agx			
Residue	Agp	Agb	Arg	Agh
Arg1	9.8	9.8	9.9	8.4
Glu2	9.6	9.6	10	9.6
Val3	9.8	8.9	11	8.9
Thr4	9.8	9.4	9.6	9.2
Val5	11	10	9.8	11
Gly7	13	13	12	12
Orn8	ND[a]	9.6	9.9	8.9
Agx9	10	9.4	9.8	8.9
Ile10	9.0	9.6	11	8.2
Leu11	9.6	9.2	11	8.5
Gln12	9.2	9.8	8.8	8.9

[a]Not determined due to signal overlap.

Table S47. The ${}^{3}J_{NH\alpha}$ Values (Hz) of the Residues in HPDUAadAgx Peptides.

	Agx			
Residue	Agp	Agb	Arg	Agh
Arg1	10	9.4	9.9	9.2
Aad2	ND[a]	6.5	9.3	9.6
Val3	9.6	8.1	8.8	8.1
Thr4	10	8.1	8.6	8.9
Val5	9.6	10	10	11
Gly7	14	14	12	12
Orn8	9.4	8.6	10	9.4
Agx9	9.6	10	5.7	7.3
Ile10	10	9.8	11	9.4
Leu11	8.9	7.7	8.8	9.2
Gln12	8.1	11	9.9	9.4

[a]Not determined due to signal overlap.

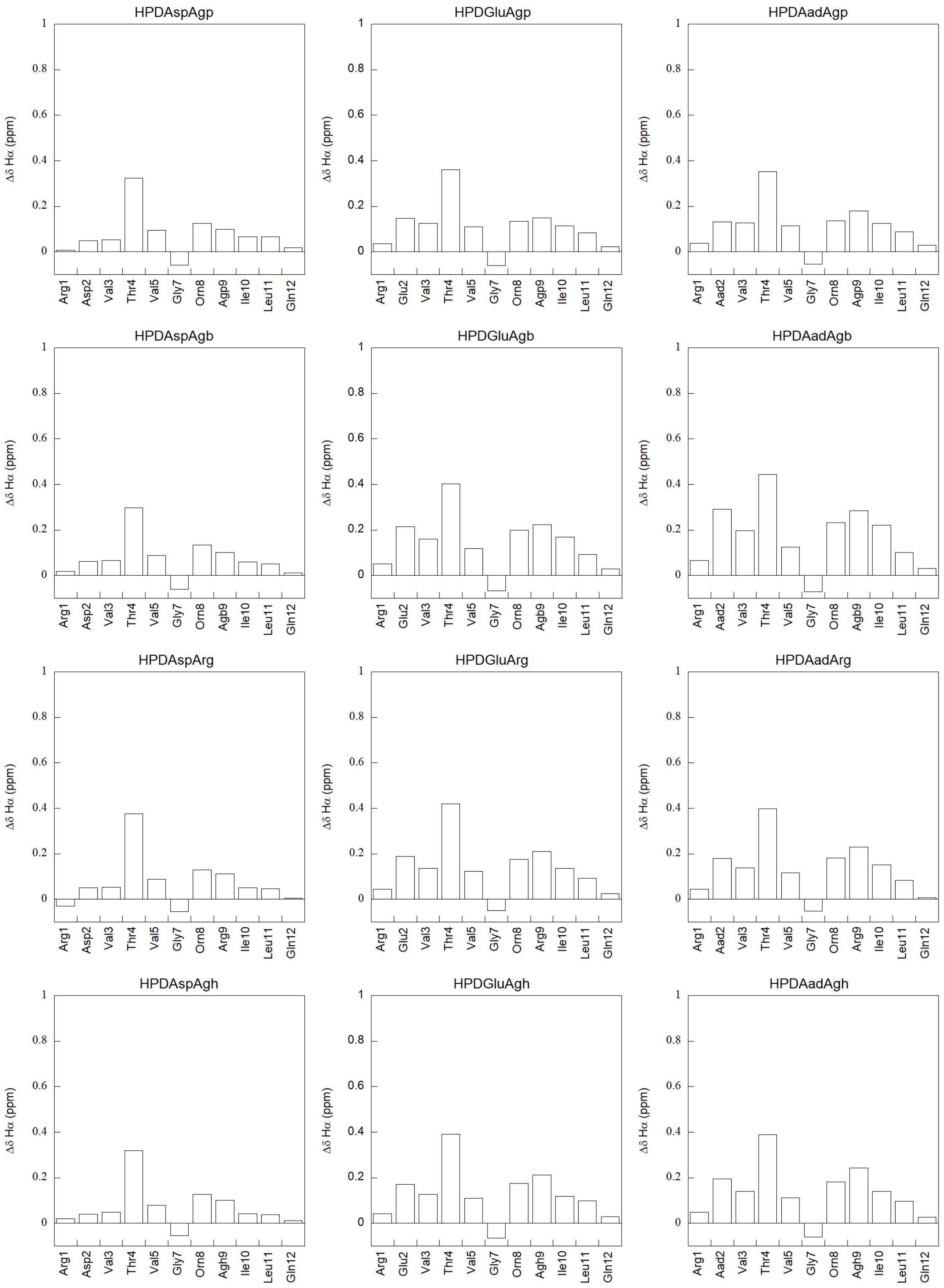

Figure S1. The Hα chemical shift deviation for the residues in the experimental HPDZbbAgx peptides.

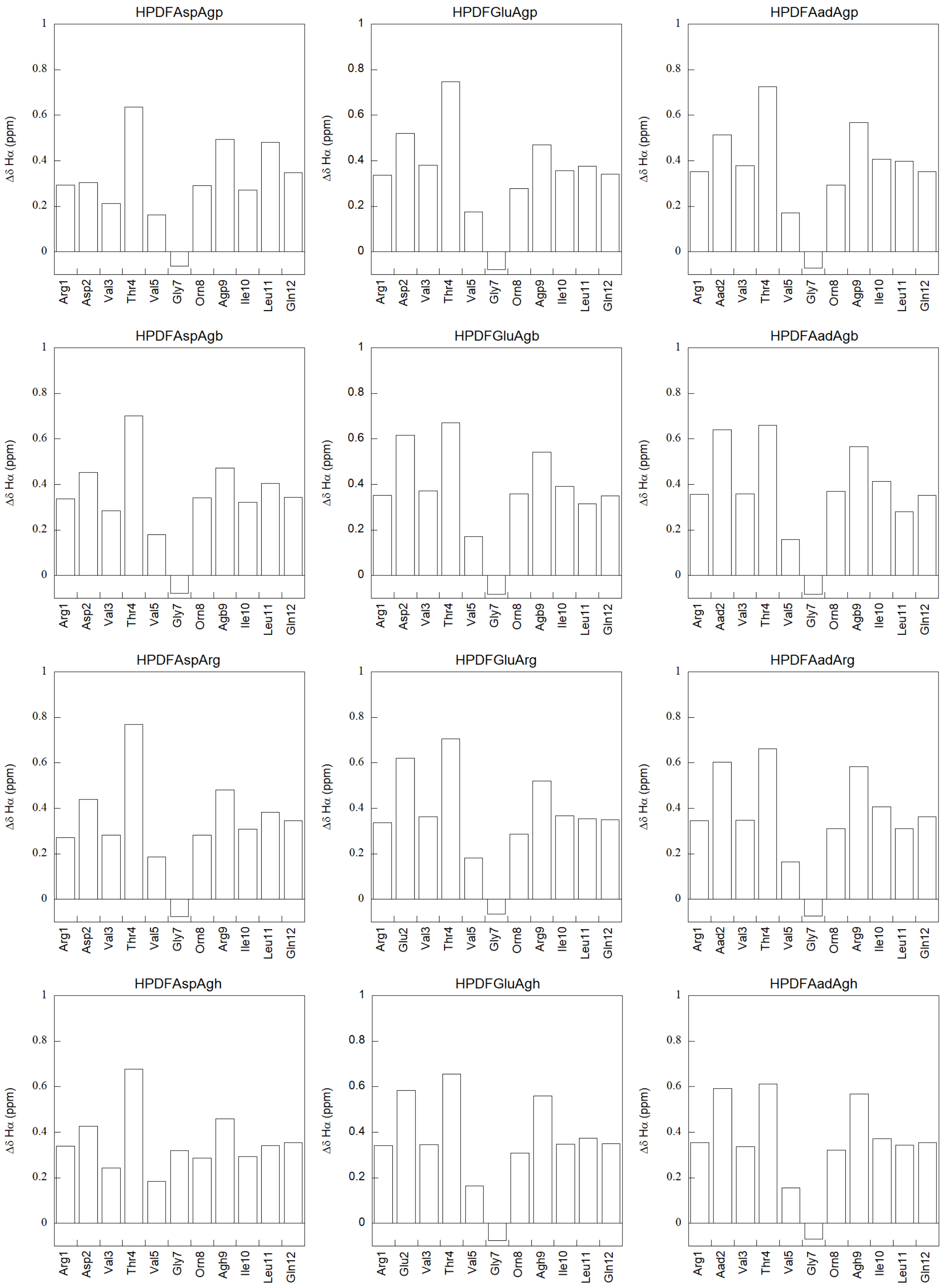

Figure S2. The Hα chemical shift deviation for the residues in the fully folded reference HPDFZbbAgx peptides.

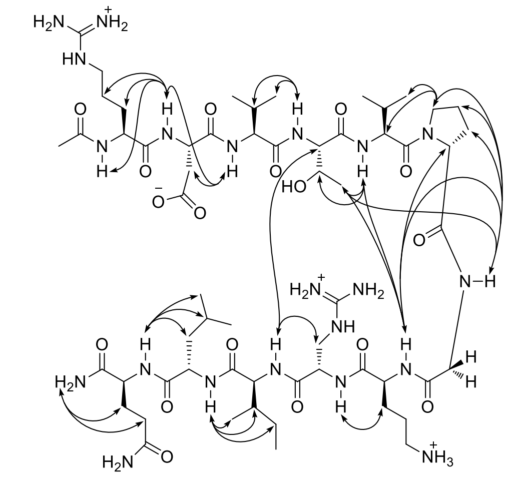

Figure S3. The inter-residue NOEs involving side chain protons for the peptide HPDAspAgp.

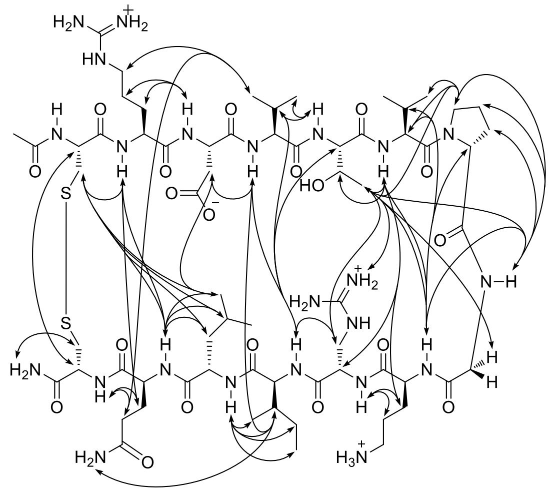

Figure S4. The inter-residue NOEs involving side chain protons for the peptide HPDFAspAgp.

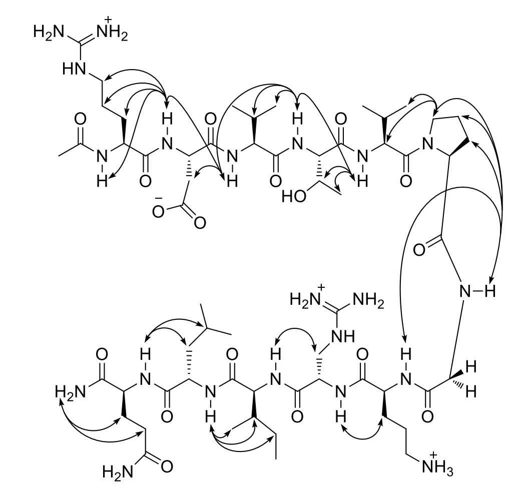

Figure S5. The inter-residue NOEs involving side chain protons for the peptide HPDUAspAgp.

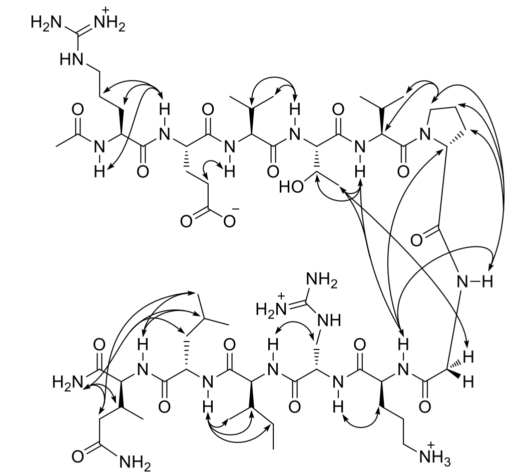

Figure S6. The inter-residue NOEs involving side chain protons for the peptide HPDGluAgp.

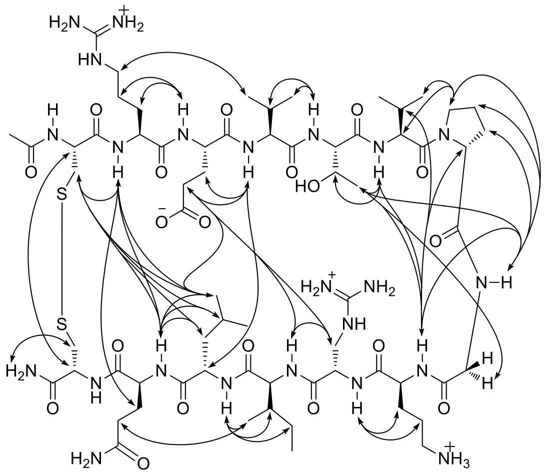

Figure S7. The inter-residue NOEs involving side chain protons for the peptide HPDFGluAgp.

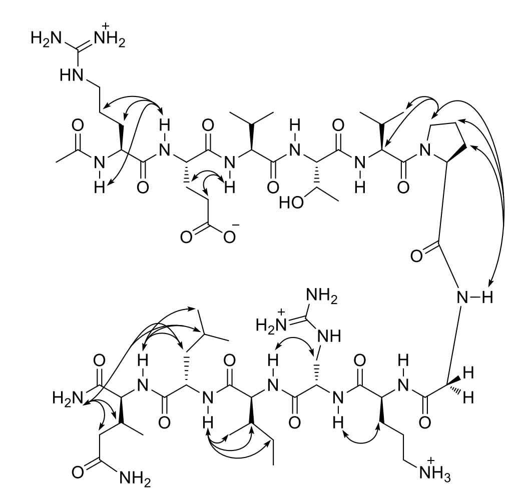

Figure S8. The inter-residue NOEs involving side chain protons for the peptide HPDUGluAgp.

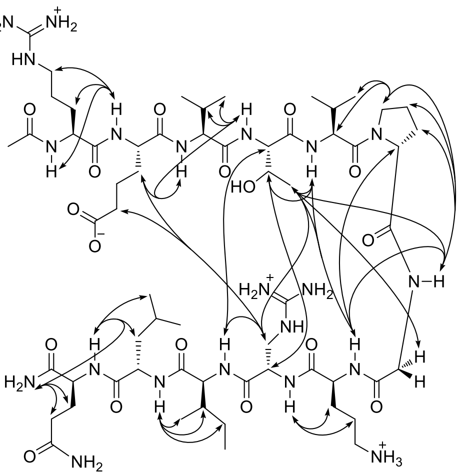

Figure S9. The inter-residue NOEs involving side chain protons for the peptide HPDAadAgp.

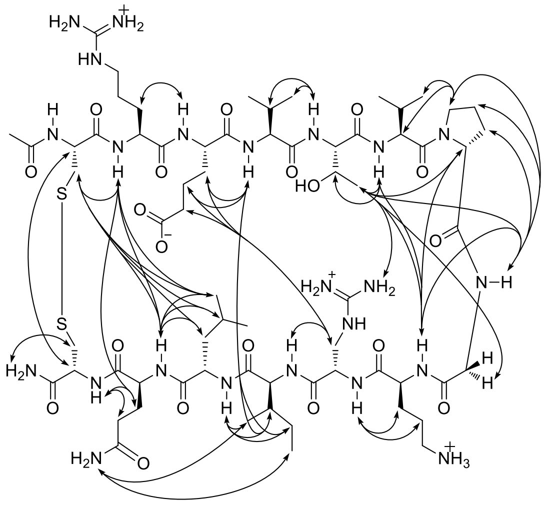

Figure S10. The inter-residue NOEs involving side chain protons for the peptide HPDFAadAgp.

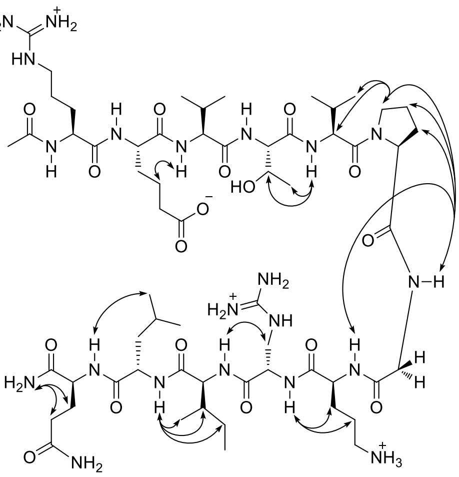

Figure S11. The inter-residue NOEs involving side chain protons for the peptide HPDUAadAgp.

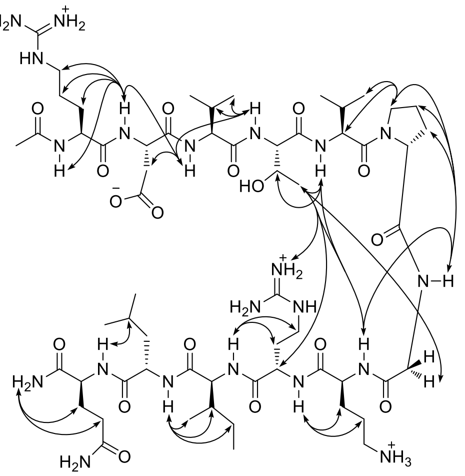

Figure S12. The inter-residue NOEs involving side chain protons for the peptide HPDAspAgb.

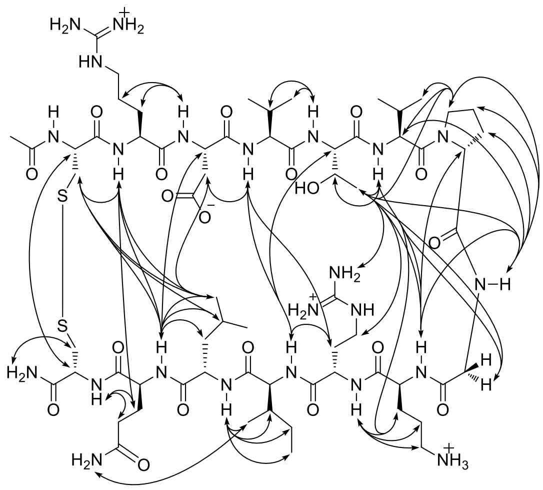

Figure S13. The inter-residue NOEs involving side chain protons for the peptide HPDFAspAgb.

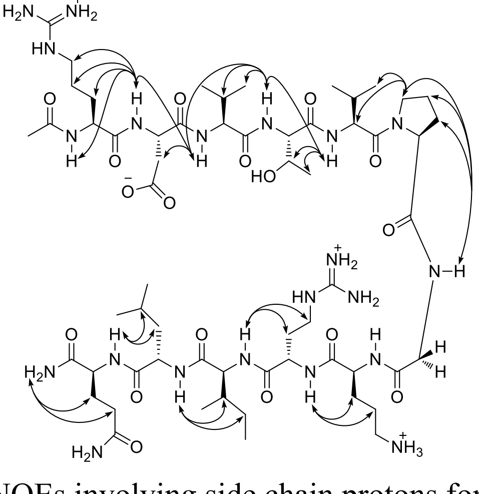

Figure S14. The inter-residue NOEs involving side chain protons for the peptide HPDUAspAgb.

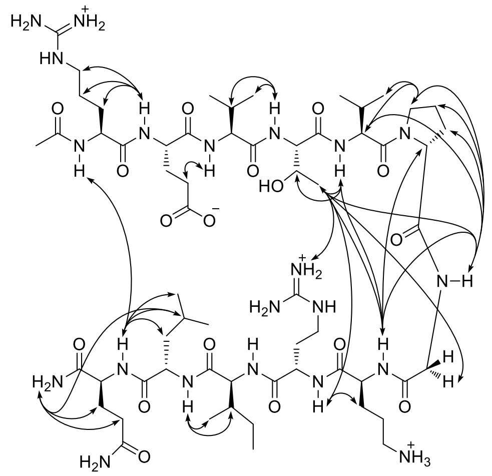

Figure S15. The inter-residue NOEs involving side chain protons for the peptide HPDGluAgb.

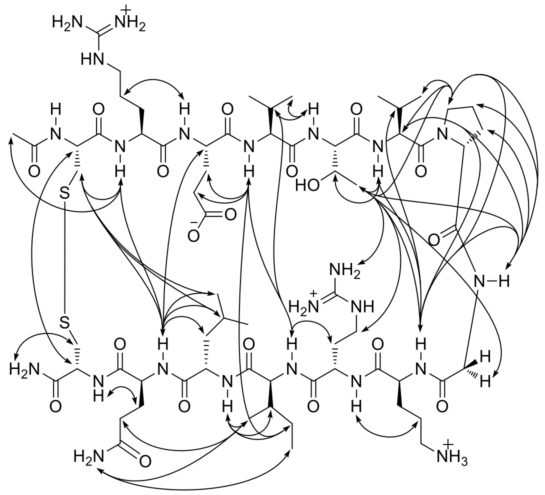

Figure S16. The inter-residue NOEs involving side chain protons for the peptide HPDFGluAgb.

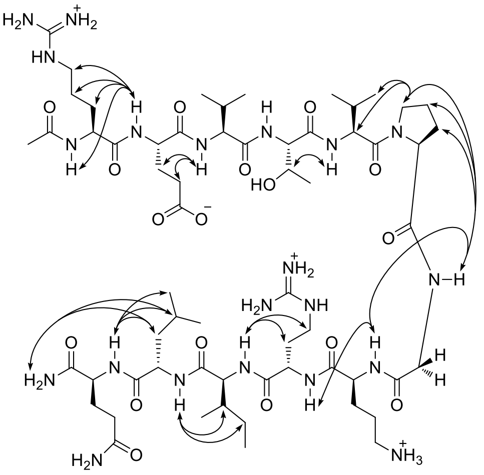

Figure S17. The inter-residue NOEs involving side chain protons for the peptide HPDUGluAgb.

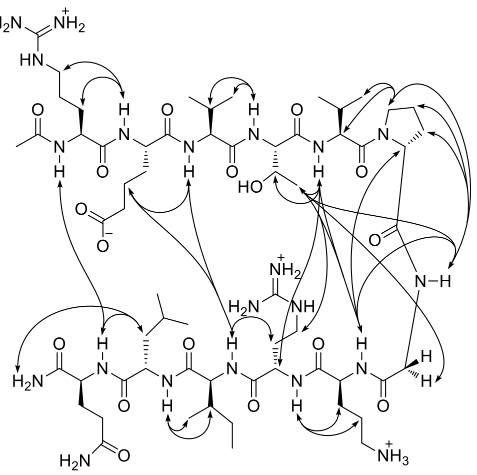

Figure S18. The inter-residue NOEs involving side chain protons for the peptide HPDAadAgb.

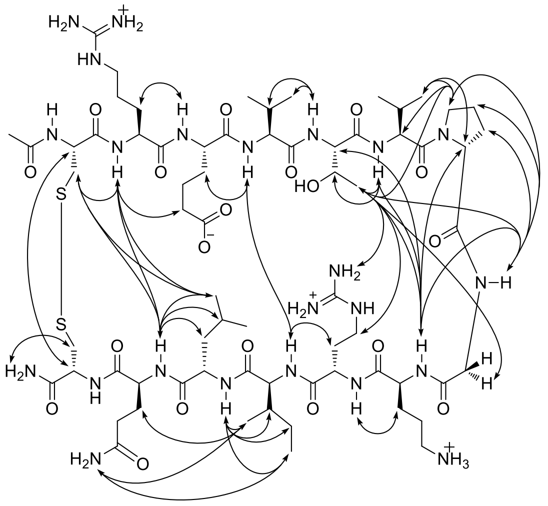

Figure S19. The inter-residue NOEs involving side chain protons for the peptide HPDFAadAgb.

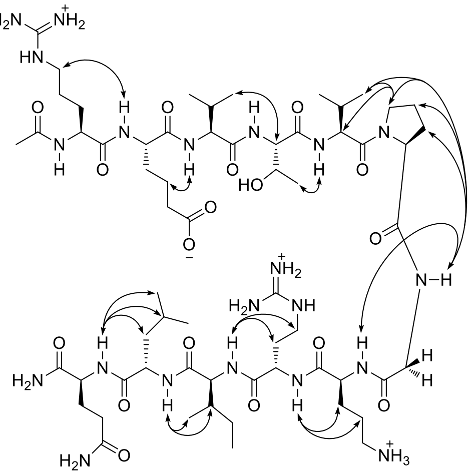

Figure S20. The inter-residue NOEs involving side chain protons for the peptide HPDUAadAgb.

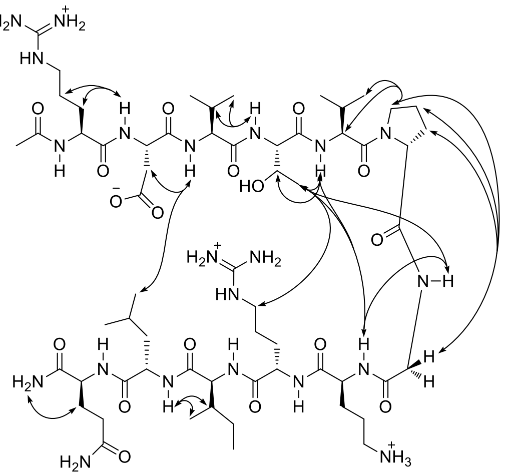

Figure S21. The inter-residue NOEs involving side chain protons for the peptide HPDAspArg.

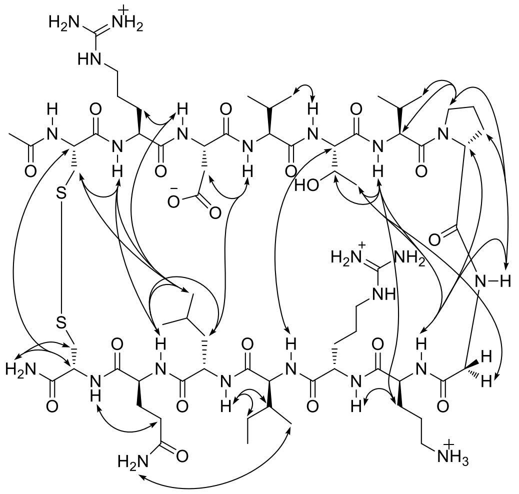

Figure S22. The inter-residue NOEs involving side chain protons for the peptide HPDFAspArg.

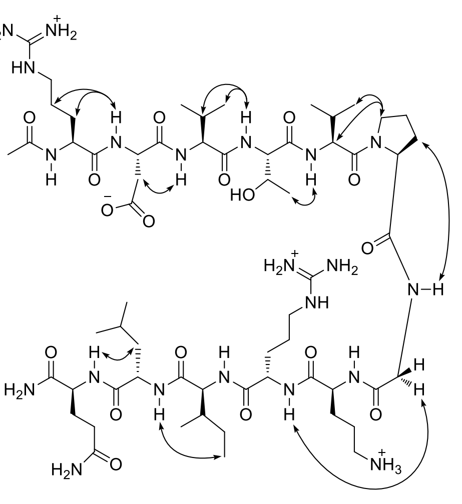

Figure S23. The inter-residue NOEs involving side chain protons for the peptide HPDUAspArg.

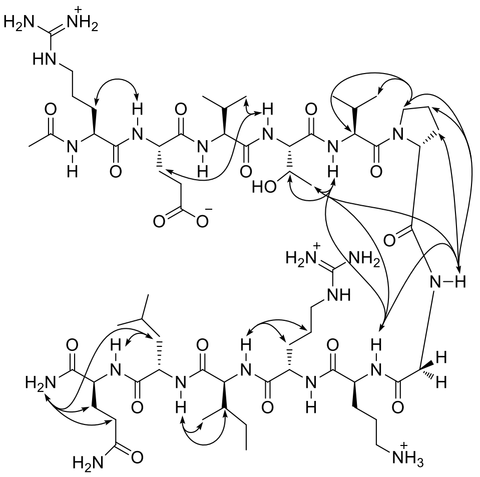

Figure S24. The inter-residue NOEs involving side chain protons for the peptide HPDGluArg.

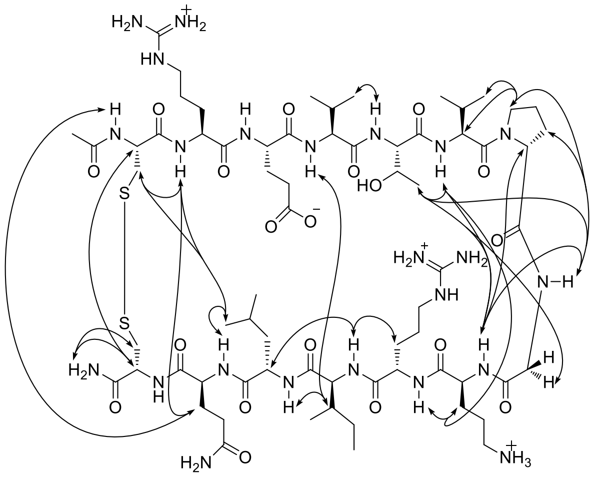

Figure S25. The inter-residue NOEs involving side chain protons for the peptide HPDFGluArg.

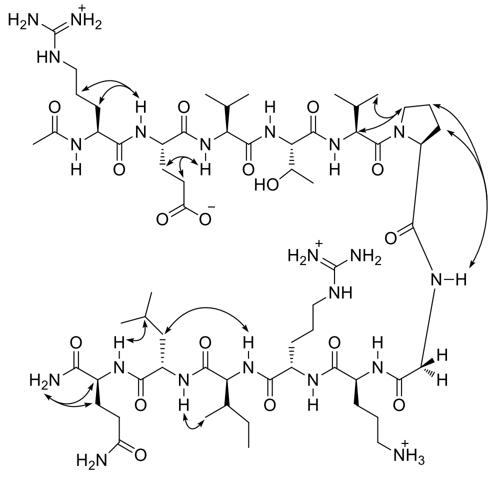

Figure S26. The inter-residue NOEs involving side chain protons for the peptide HPDUGluArg

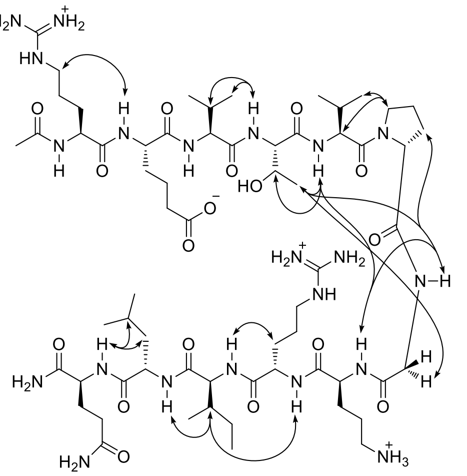

Figure S27. The inter-residue NOEs involving side chain protons for the peptide HPDAadArg.

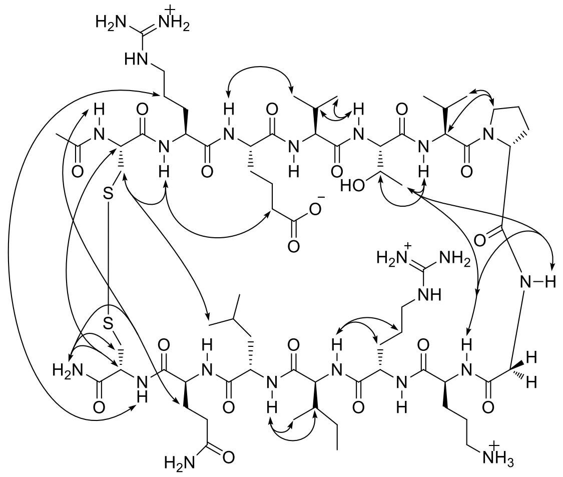

Figure S28. The inter-residue NOEs involving side chain protons for the peptide HPDFAadArg.

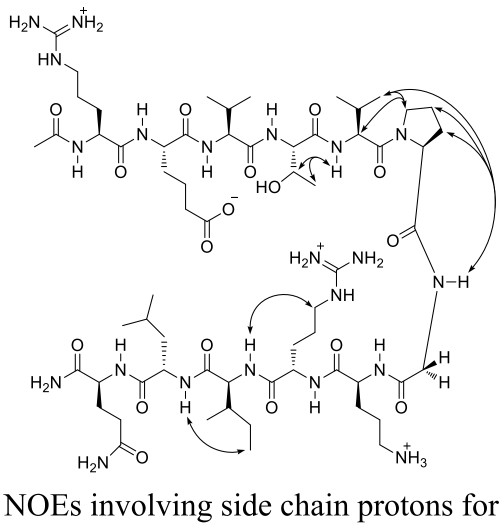

Figure S29. The inter-residue NOEs involving side chain protons for the peptide HPDUAadArg.

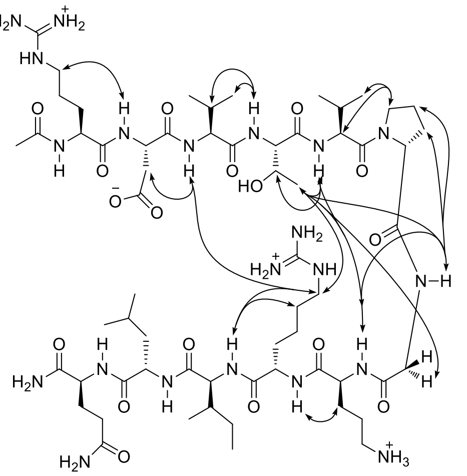

Figure S30. The inter-residue NOEs involving side chain protons for the peptide HPDAspAgh.

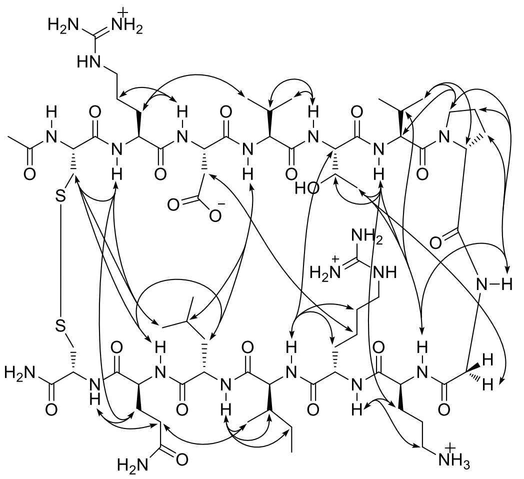

Figure S31. The inter-residue NOEs involving side chain protons for the peptide HPDFAspAgh.

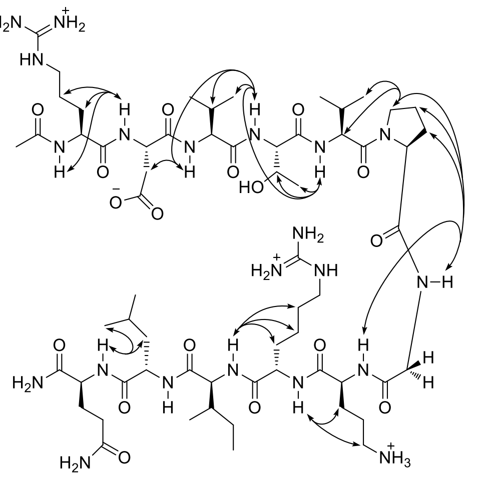

Figure S32. The inter-residue NOEs involving side chain protons for the peptide HPDUAspAgh.

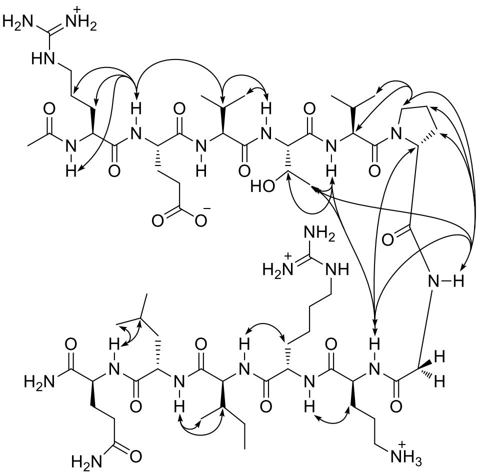

Figure S33. The inter-residue NOEs involving side chain protons for the peptide HPDGluAgh.

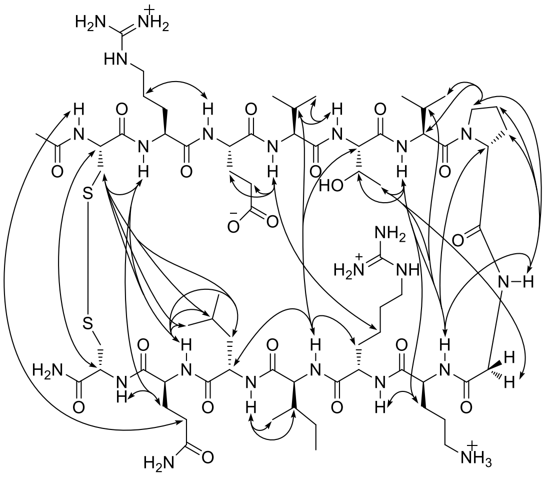

Figure S34. The inter-residue NOEs involving side chain protons for the peptide HPDFGluAgh.

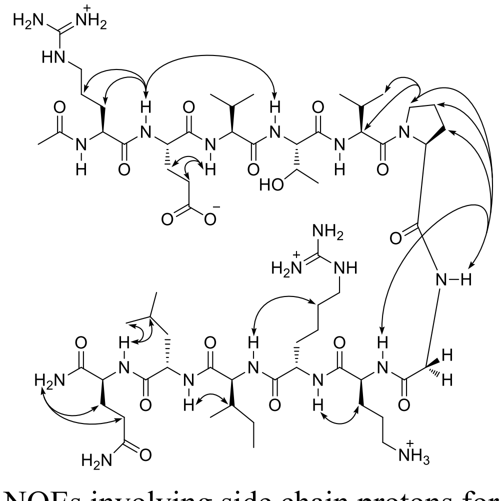

Figure S35. The inter-residue NOEs involving side chain protons for the peptide HPDUGluAgh.

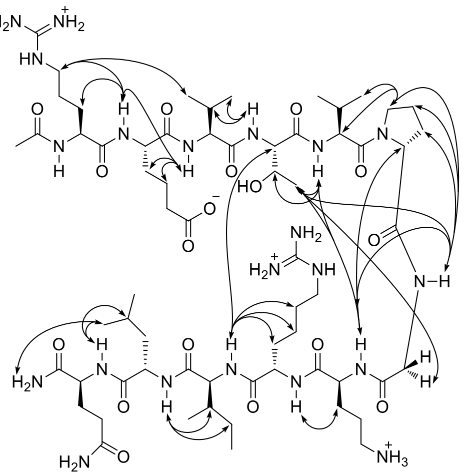

Figure S36. The inter-residue NOEs involving side chain protons for the peptide HPDAadAgh.

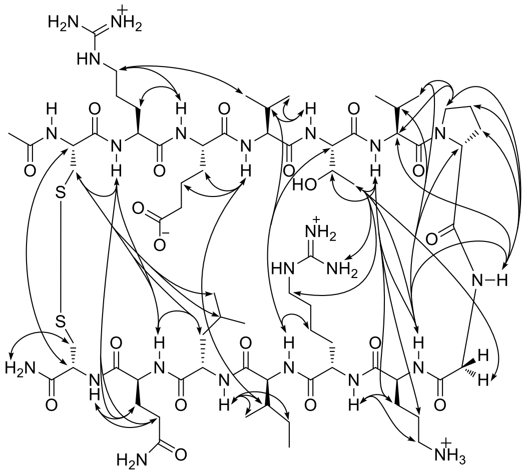

Figure S37. The inter-residue NOEs involving side chain protons for the peptide HPDFAadAgh.

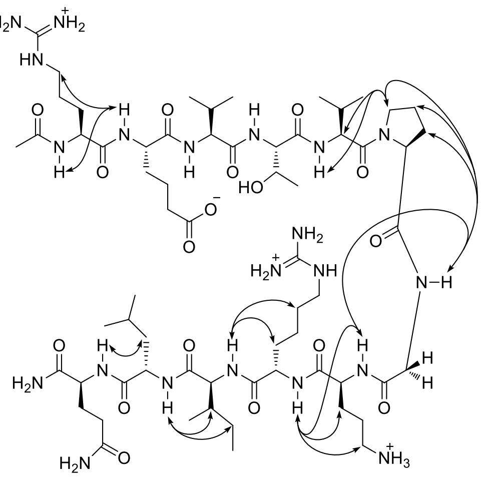

Figure S38. The inter-residue NOEs involving side chain protons for the peptide HPDUAadAgh.

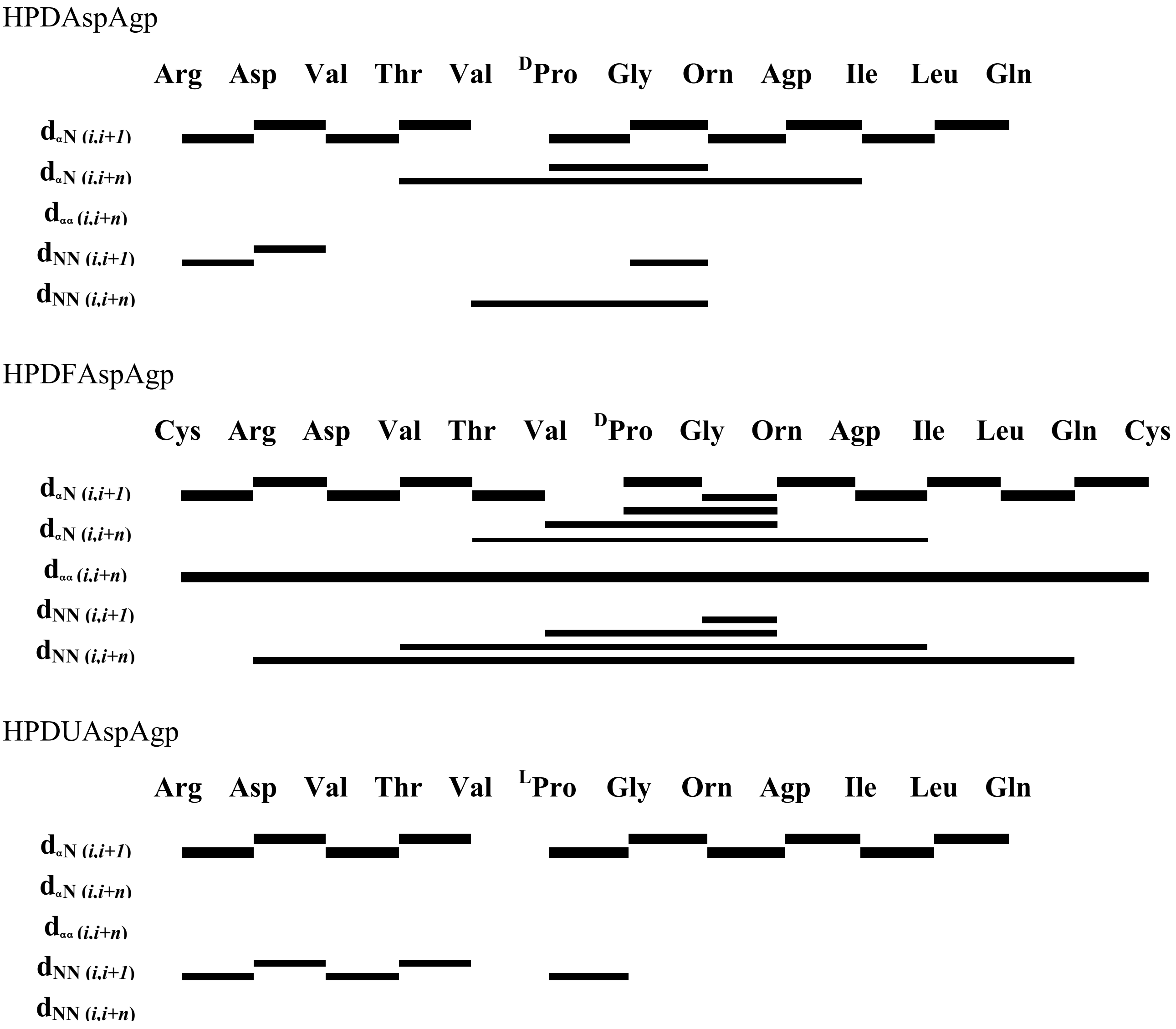

Figure S39. Wüthrich diagram of peptides HPDAspAgp, HPDFAspAgp, HPDUAspAgp. Only the backbone NOE connectivities are shown.

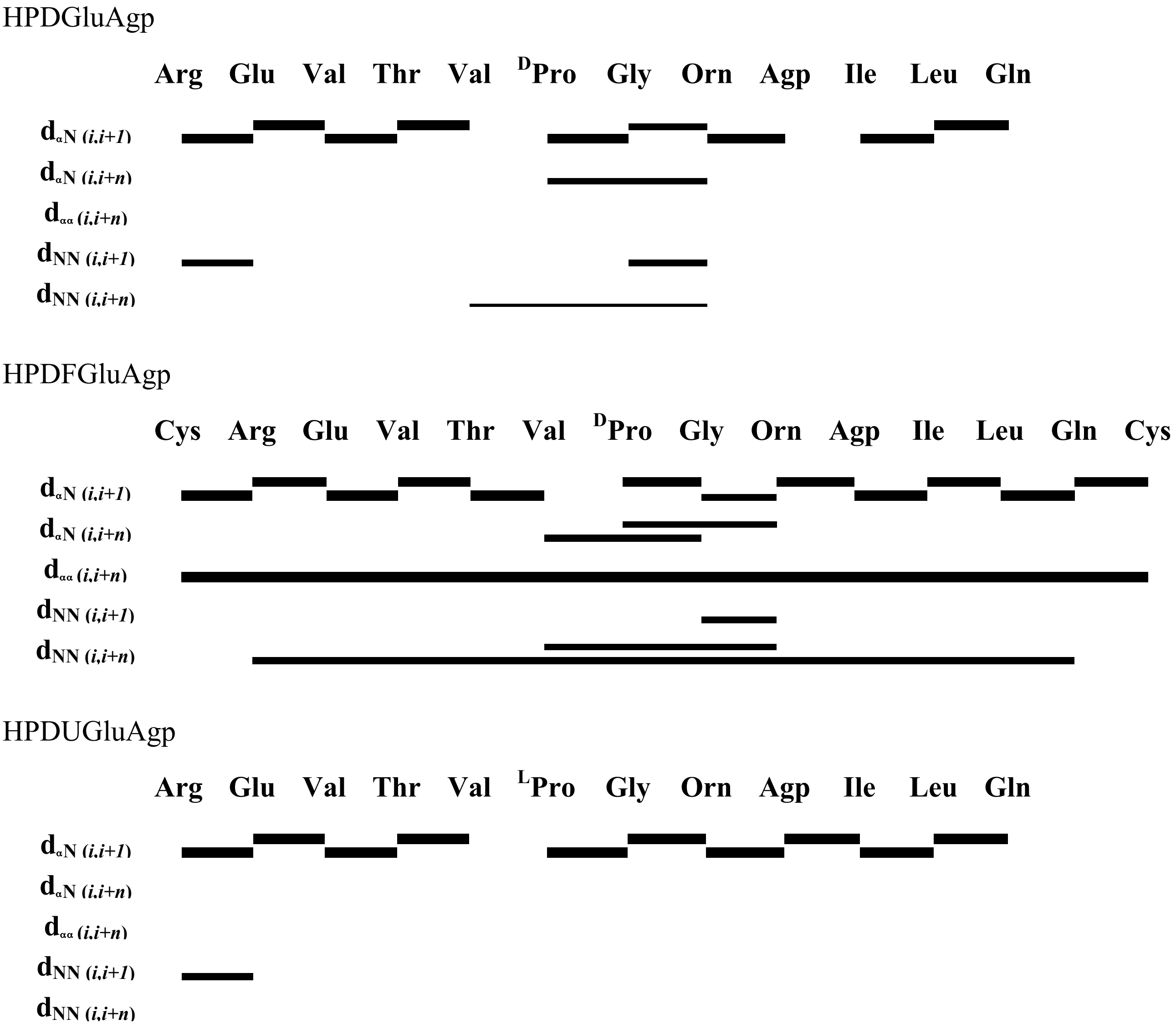

Figure S40. Wüthrich diagram of peptides HPDGluAgp, HPDFGluAgp, HPDUGluAgp. Only the backbone NOE connectivities are shown.

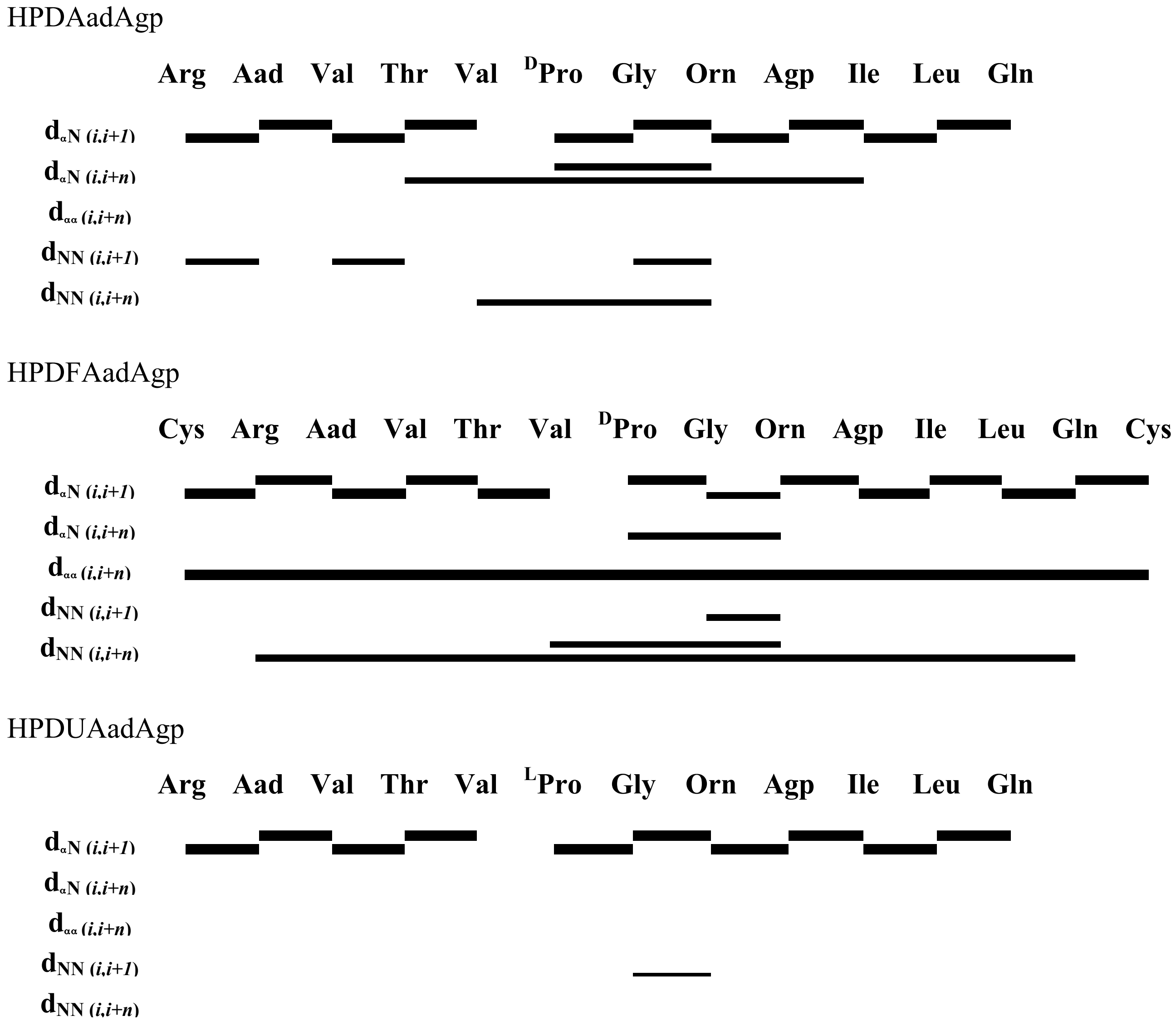

Figure S41. Wüthrich diagram of peptides HPDAadAgp, HPDFAadAgp, HPDUAadAgp. Only the backbone NOE connectivities are shown.

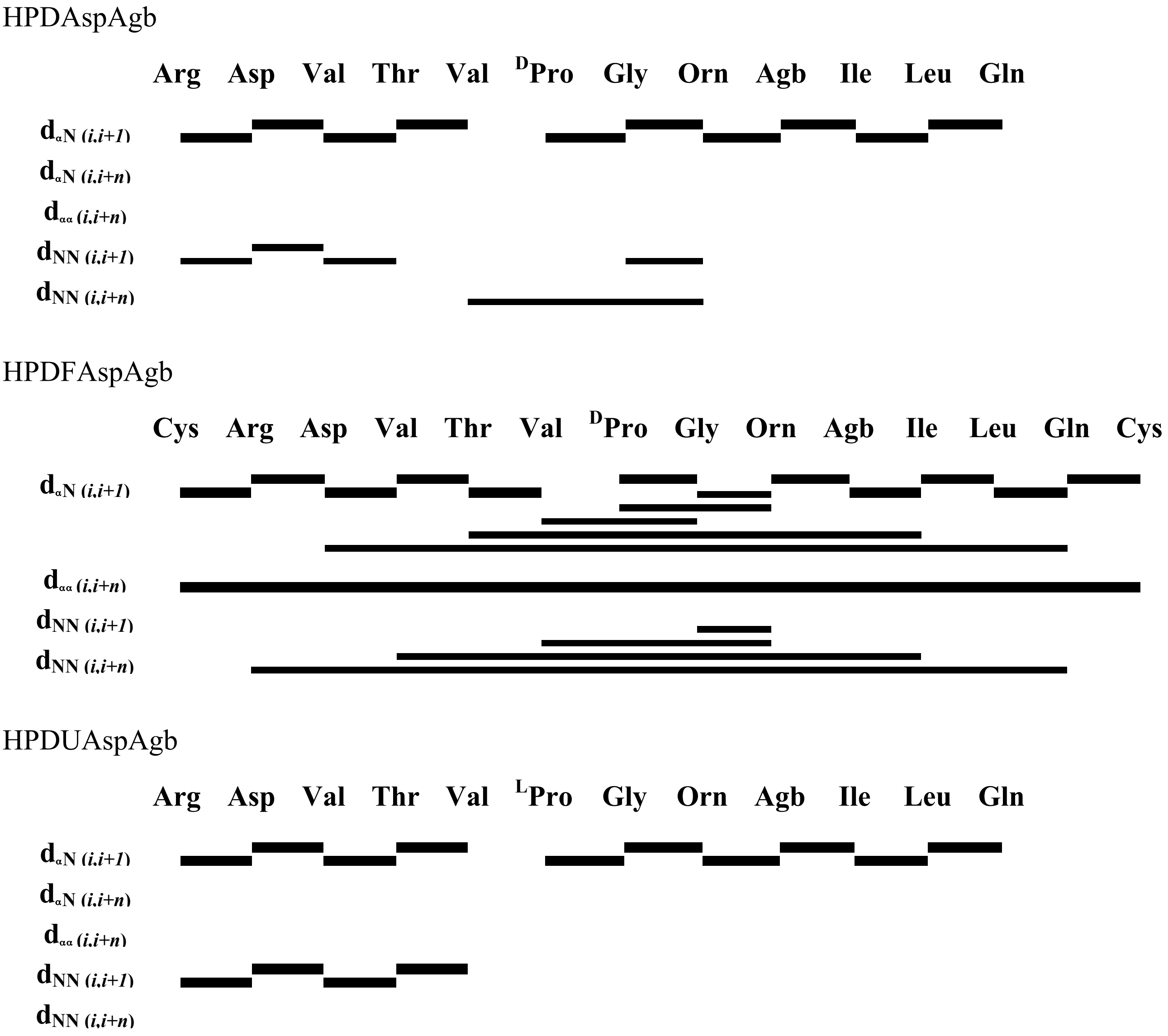

Figure S42. Wüthrich diagram of peptides HPDAspAgb, HPDFAspAgb, HPDUAspAgb. Only the backbone NOE connectivities are shown.

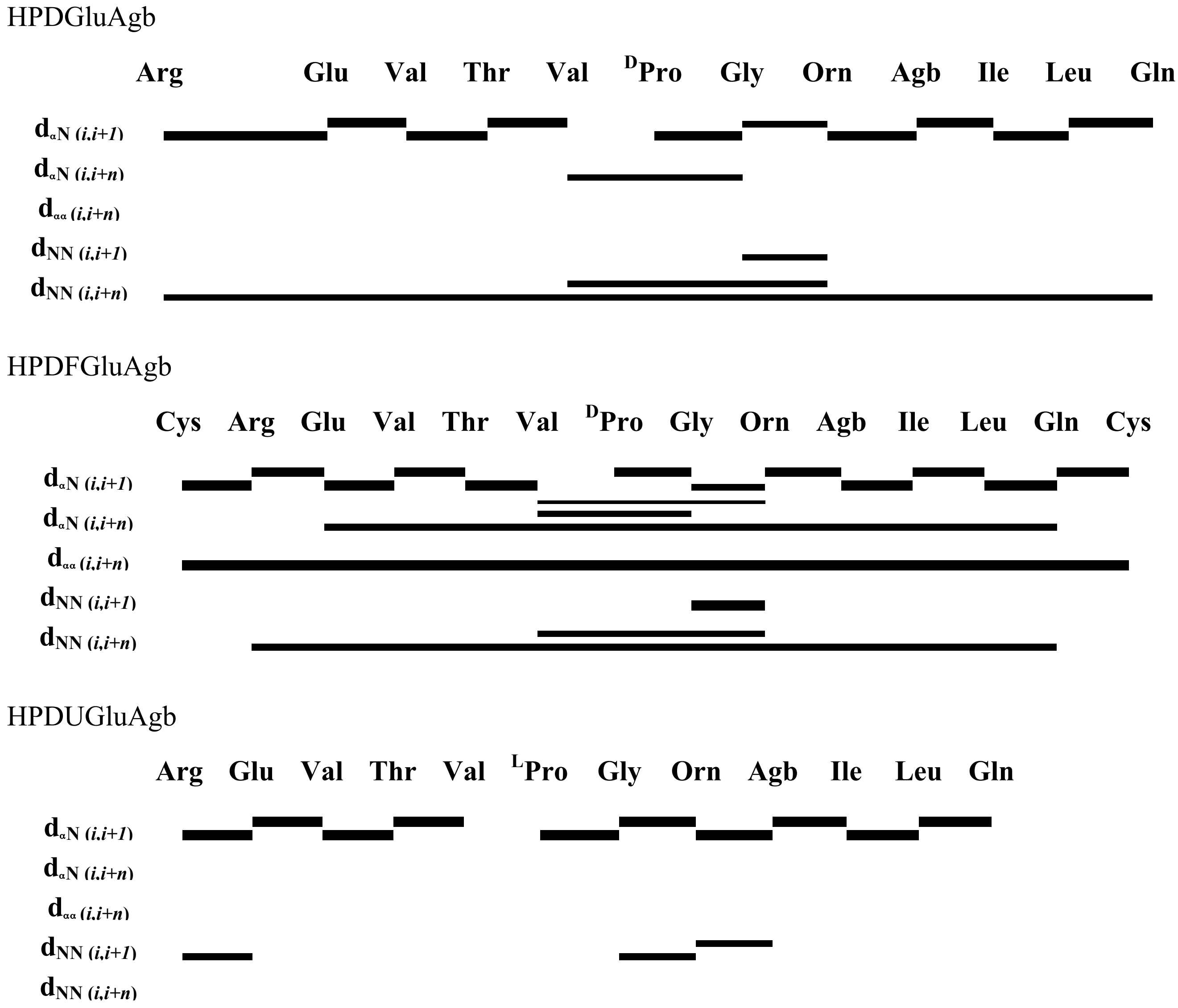

Figure S43. Wüthrich diagram of peptides HPDGluAgb, HPDFGluAgb, HPDUGluAgb. Only the backbone NOE connectivities are shown.

HPDAadAgb

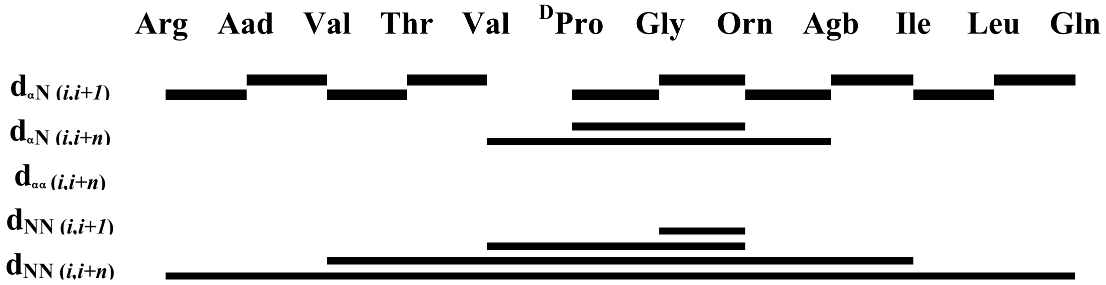

HPDFAadAgb

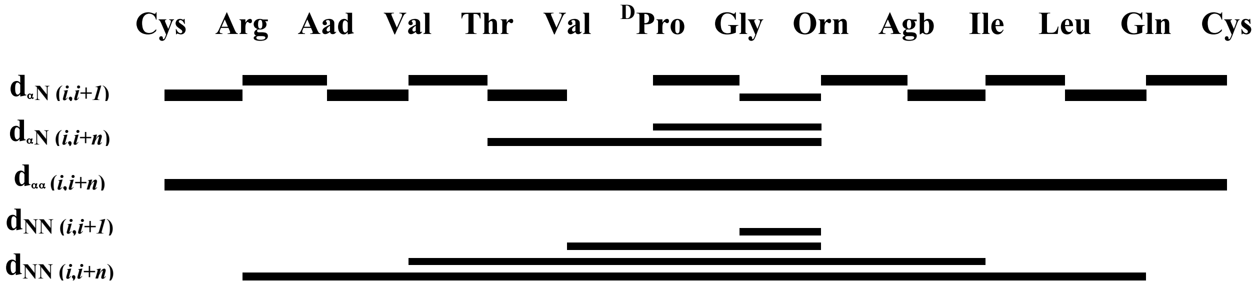

HPDUAadAgb

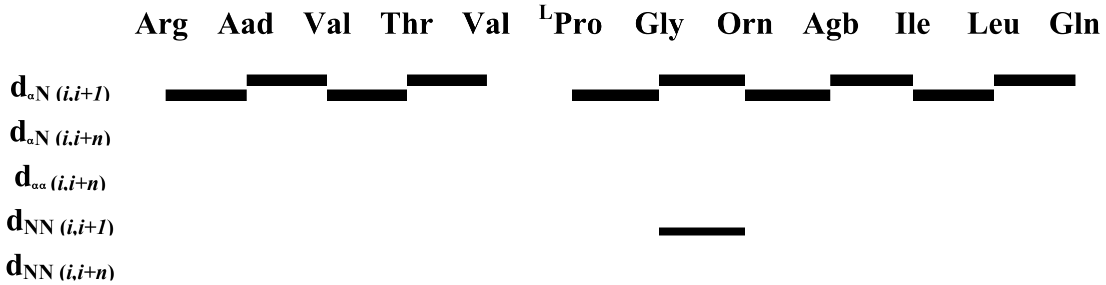

Figure S44. Wüthrich diagram of peptides HPDAadAgb, HPDFAadAgb, HPDUAadAgb. Only the backbone NOE connectivities are shown.

HPDAspArg

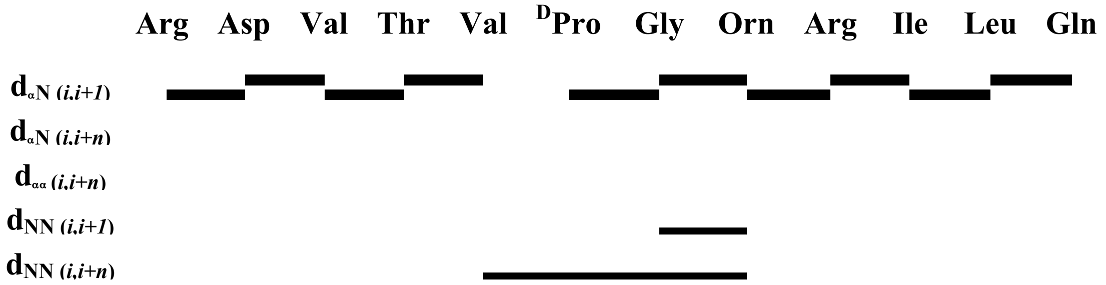

HPDFAspArg

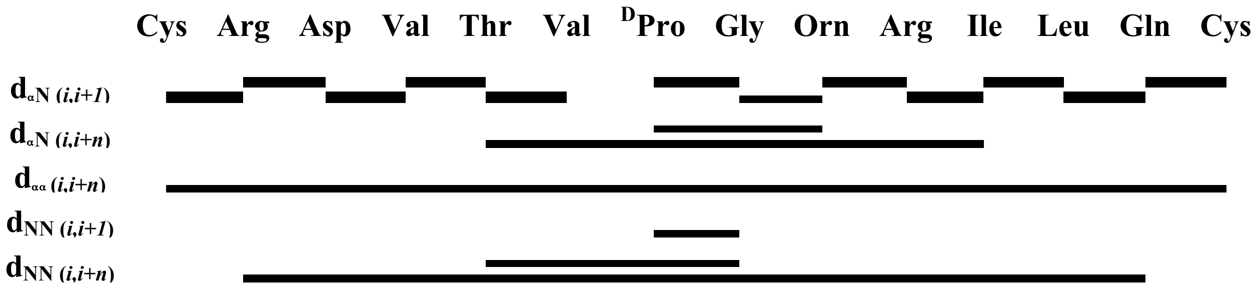

HPDUAspArg

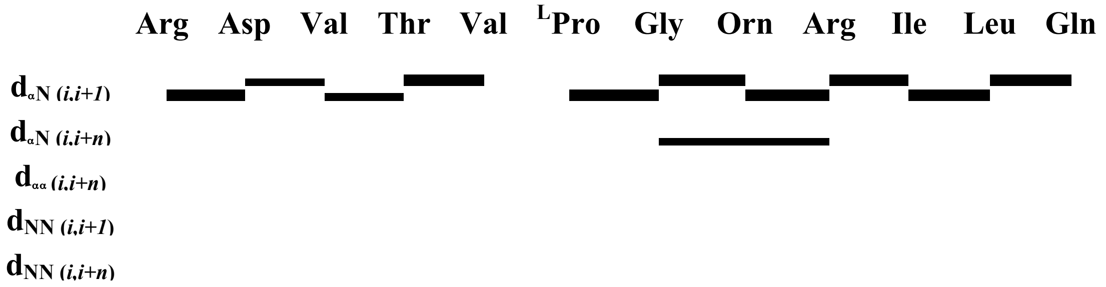

Figure S45. Wüthrich diagram of peptides HPDAspArg, HPDFAspArg, HPDUAspArg. Only the backbone NOE connectivities are shown.

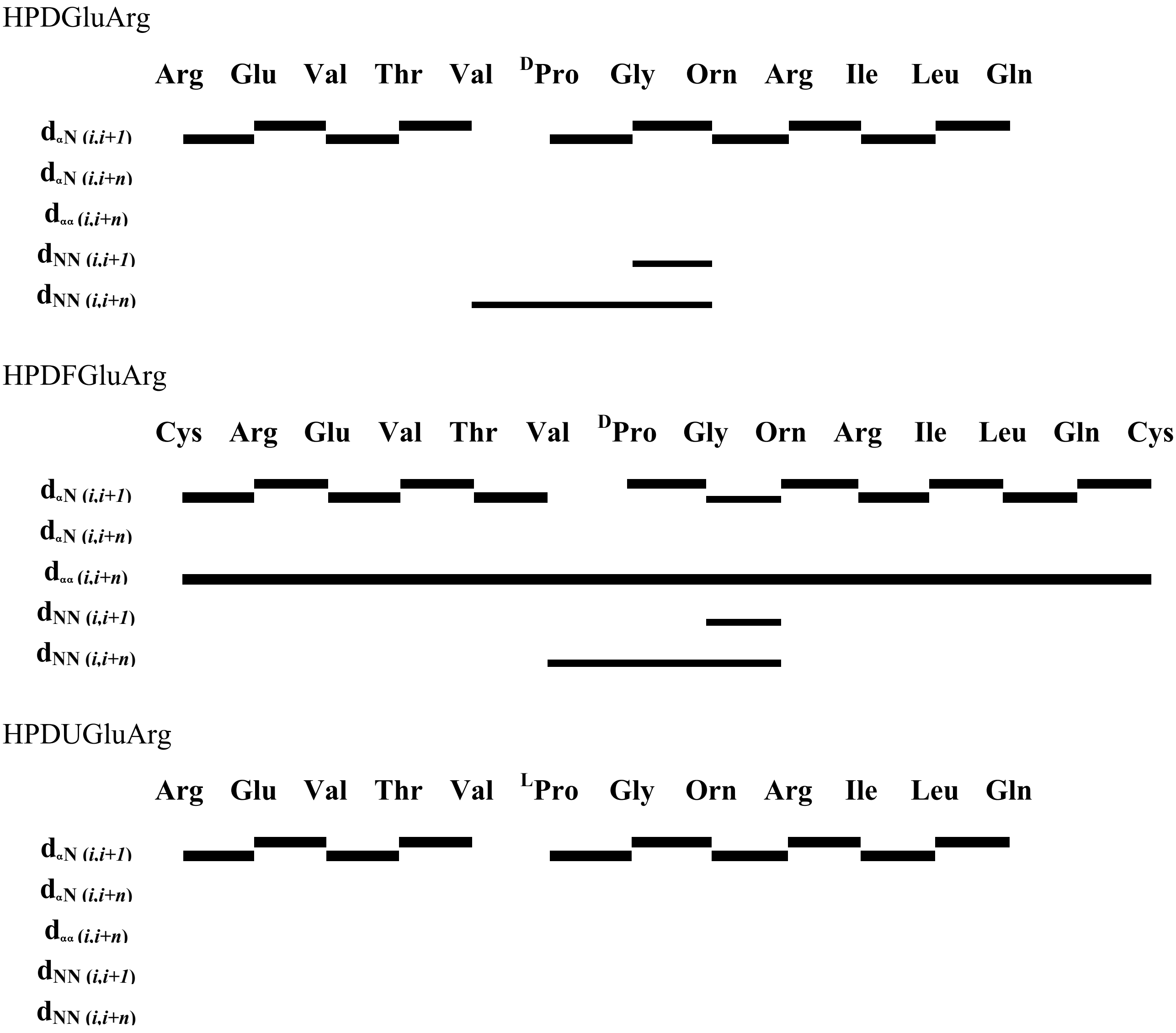

Figure S46. Wüthrich diagram of peptides HPDGluArg, HPDFGluArg, HPDUGluArg. Only the backbone NOE connectivities are shown.

HPDAadArg

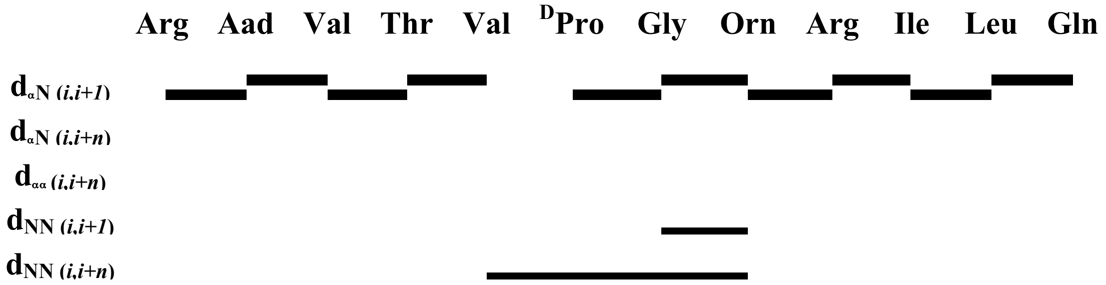

HPDFAadArg

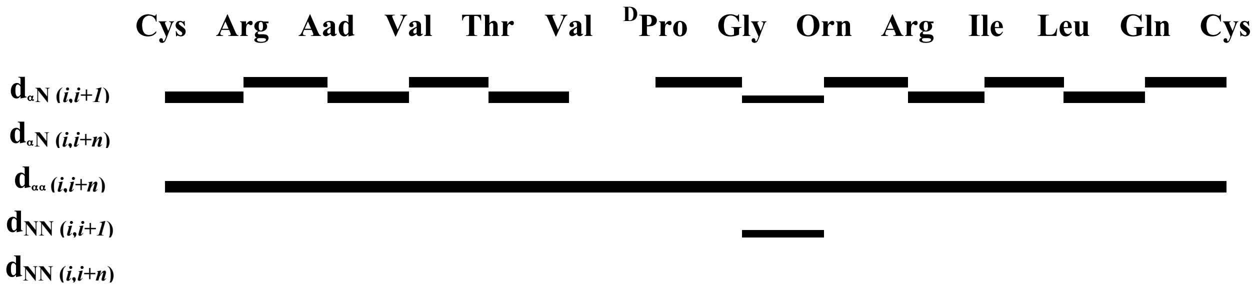

HPDUAadArg

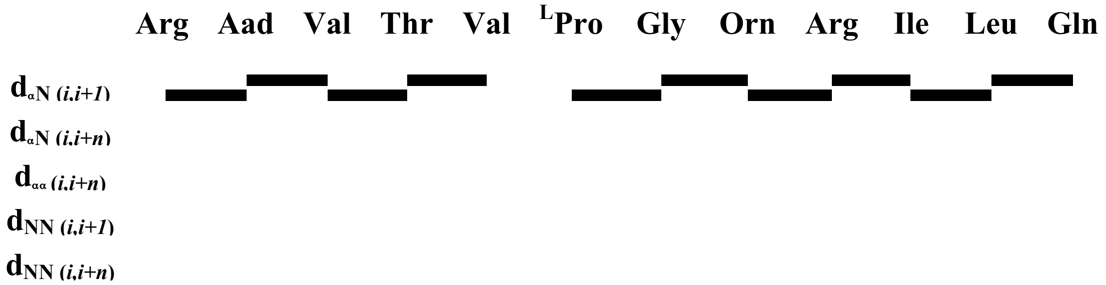

Figure S47. Wüthrich diagram of peptides HPDAadArg, HPDFAadArg, HPDUAadArg. Only the backbone NOE connectivities are shown.

HPDAspAgh

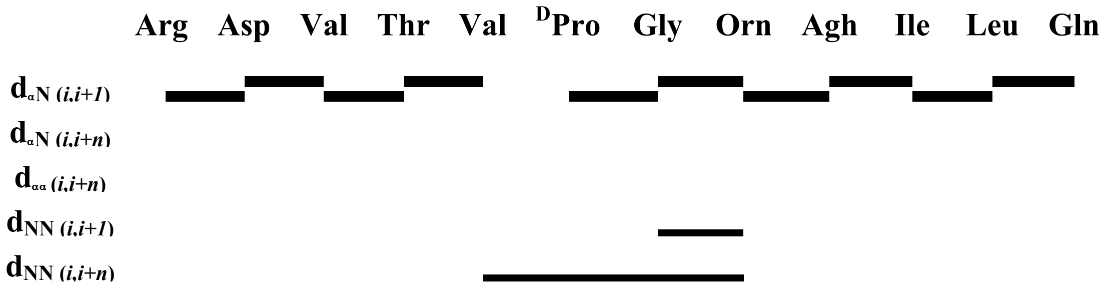

HPDFAspAgh

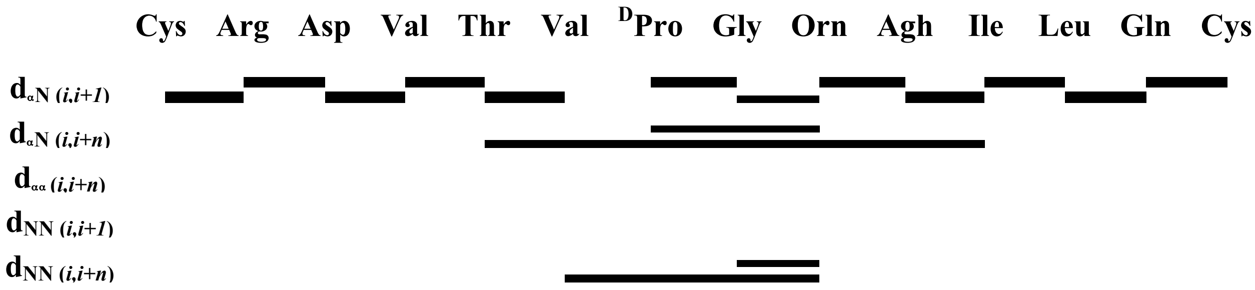

HPDUAspAgh

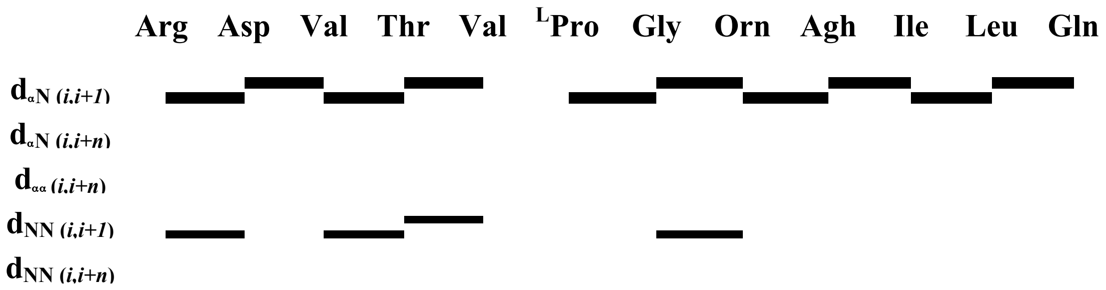

Figure S48. Wüthrich diagram of peptides HPDAspAgh, HPDFAspAgh, HPDUAspAgh. Only the backbone NOE connectivities are shown.

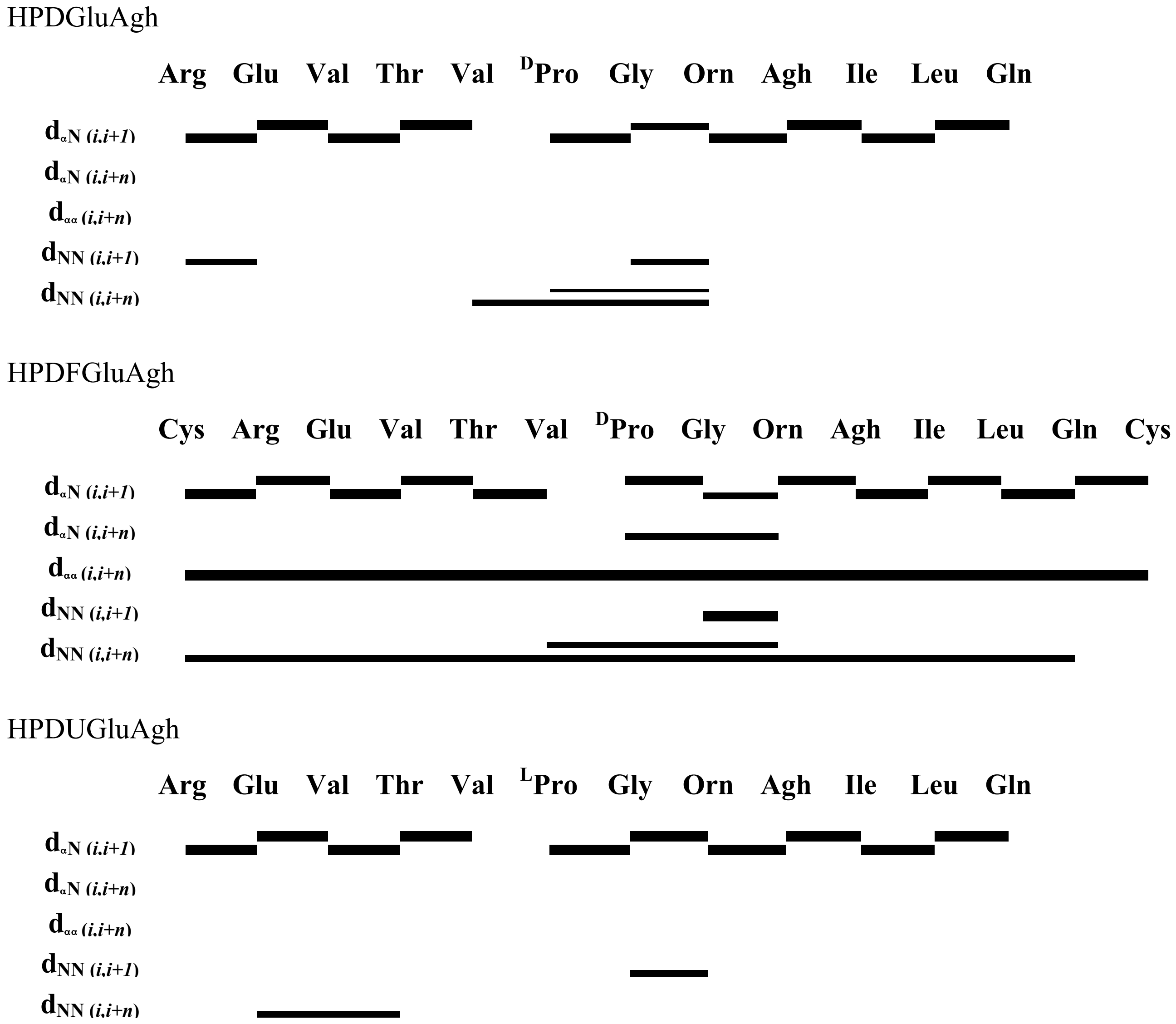

Figure S49. Wüthrich diagram of peptides HPDGluAgh, HPDFGluAgh, HPDUGluAgh. Only the backbone NOE connectivities are shown.

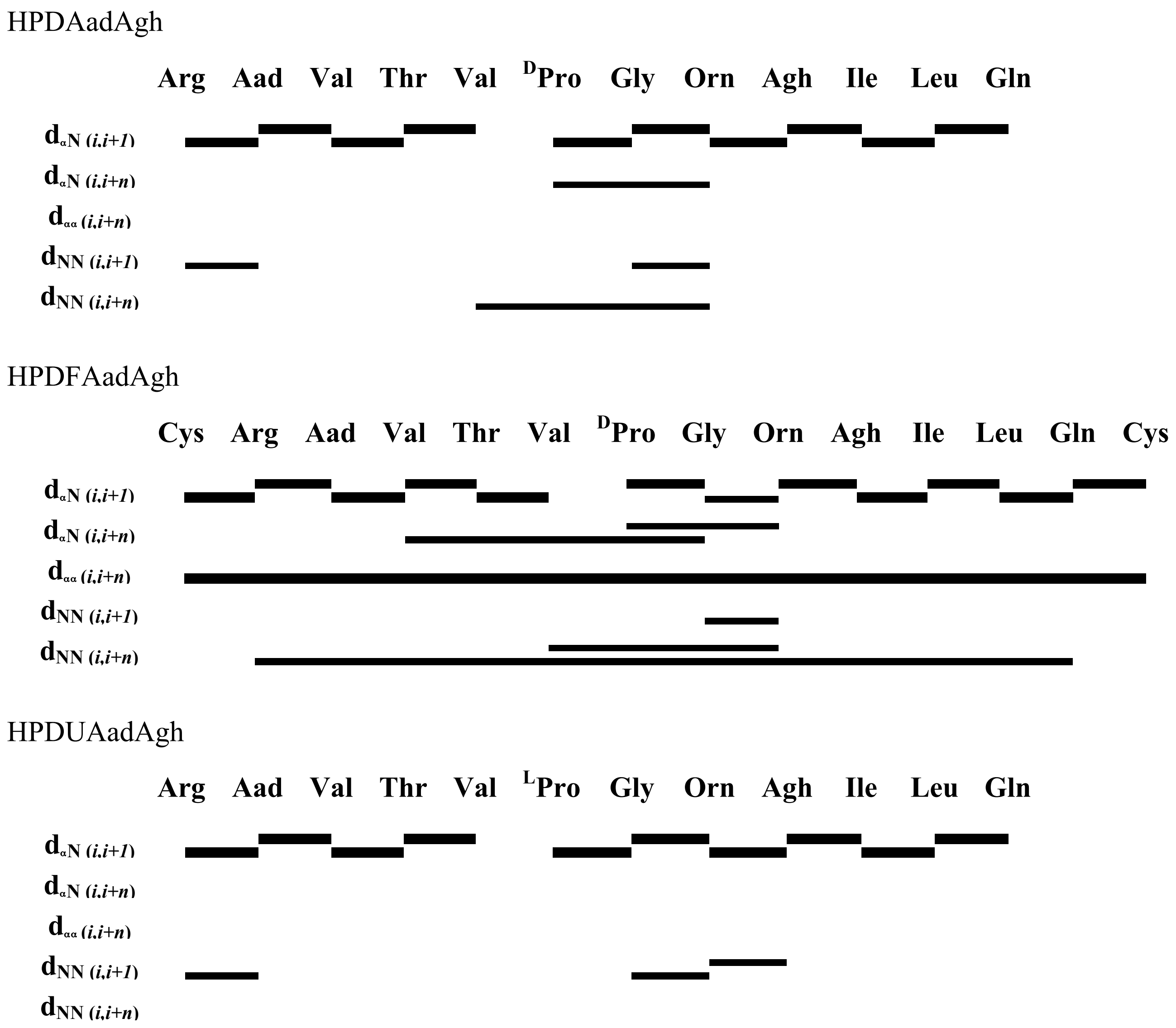

Figure S50. Wüthrich diagram of peptides HPDAadAgh, HPDFAadAgh, HPDUAadAgh. Only the backbone NOE connectivities are shown.

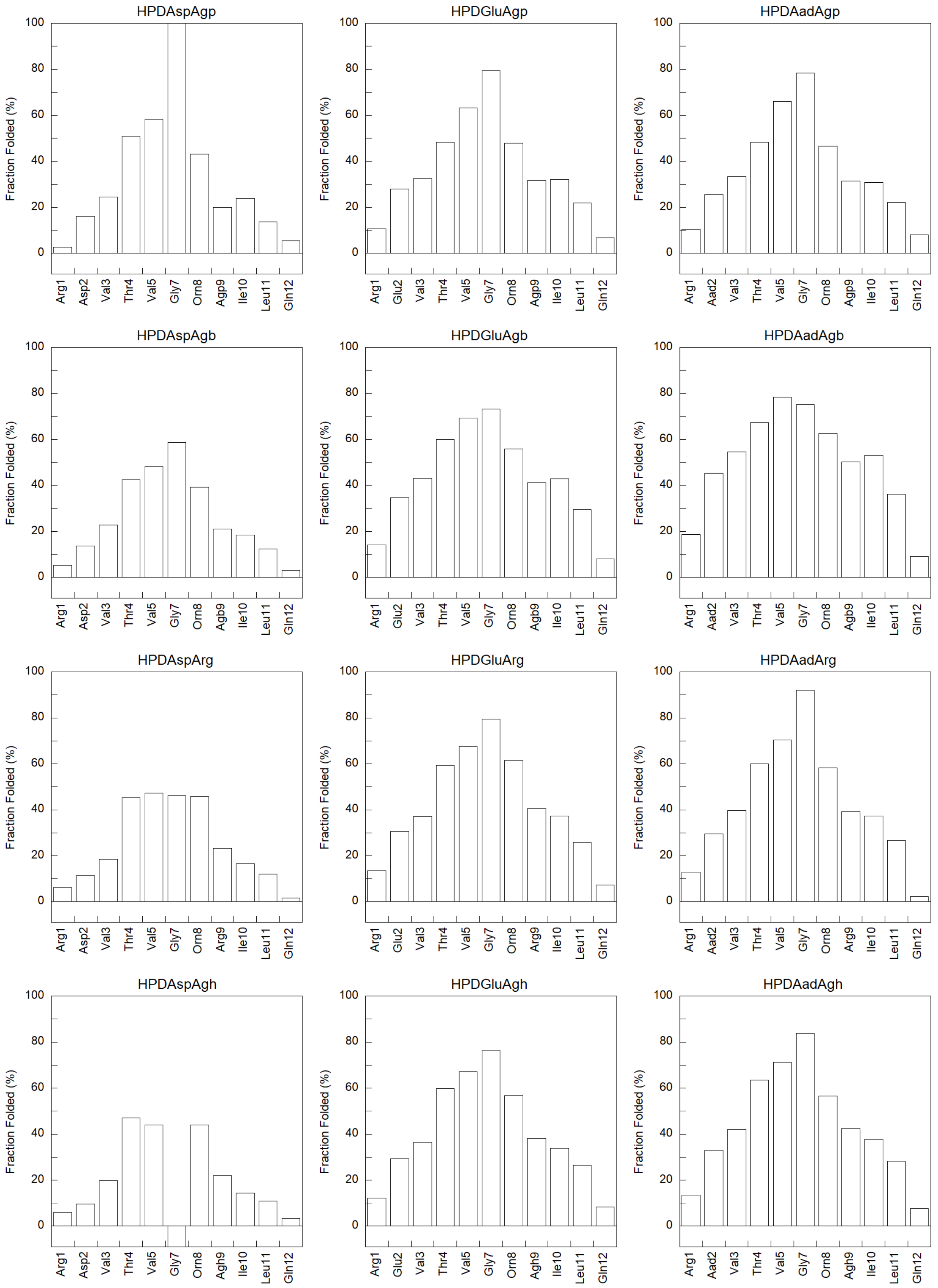

Figure S51. The fraction folded of all residues for the HPDZbbXaa peptides.

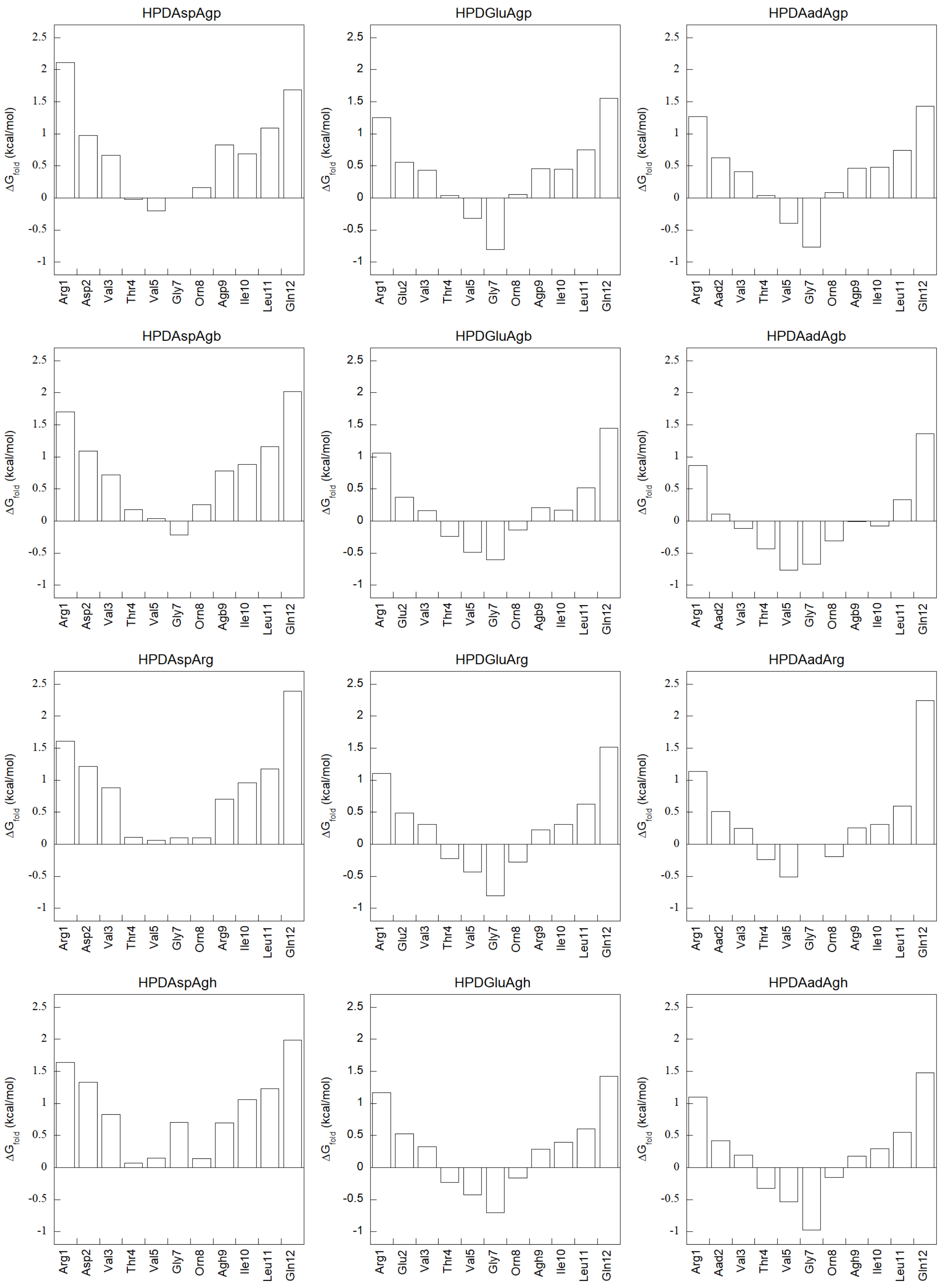

Figure S52. The ΔG_{fold} of all residues for the HPDZbbXaa peptides.

Material and Methods

General Section

All reagents and solvents were used without purification. Diisopropylethylamine (DIEA), piperidine, trifluoroacetic acid (TFA), acetic anhydride (Ac_2O) were purchased from Acros. Dimethylformamide (DMF), methanol, and acetonitrile were purchased from Merck. N-9-fluorenylmethoxycarbonyl (Fmoc)-amino acids except those indicated otherwise were from Novabiochem. Fmoc-L-Agp(Pbf,Boc)-OH and Fmoc-L-Agb(Pbf,Boc)-OH were from Iris Biotec. N_α-Fmoc-aminoadipic acid-δ-t-butyl ester was from BaChem. 1-Hydroxybenzotriazole (HOBt), 2-(1H-benzotriazole-1-yl)-1, 1, 3, 3 -tetramethyluronium hexafluorophosphate (HBTU), NovaSyn® TGR resin were from NovaBiochem. Hexanes were from Duksan. Analytical reverse phase (RP)-HPLC was performed on an Agilent 1200 series chromatography system using a Vydac C_{18} column (4.6 mm diameter, 250 mm length). Preparative RP-HPLC was performed on Waters Breeze chromatography system using a Seppak® plus short tC_{18} cartridges, Vydac C_4 or C_{18} column (22 mm diameter, 250 mm length) Mass spectrometry of the peptides was performed on a matrix-assisted laser desorption ionization time-of-fight (MALDI-TOF) (Bruker BIFLEX) using α-cyano-4-hydroxycinnamic acid as the matrix. 2-Dimensional nuclear magnetic resonance spectroscopy experiments were performed on the Bruker AV III 800MHz spectrometer.

Peptide Synthesis

The peptides were synthesized by solid phase peptide synthesis using Fmoc-based chemistry [1, 2]. NovaSyn® TGR resin (0.050 mmol) was swollen in N, N-dimethylformamide (DMF, 3 mL) for 30 minutes. A mixture of 3 equivalents of the appropriately protected Fmoc-amino acid, HOBt, and HBTU was dissolved in DMF (1 mL). Diisopropylethylamine (DIEA, 8 equivalents) was then added to the solution and mixed thoroughly. The solution was then applied to the resin. The vial that contained the solution was rinsed with DMF (2x1 mL) and added to the reaction. The first coupling was carried out for 8 hours. The 8th to 14th residues were coupled for 1.5 hours. Other residues were coupled for 45 minutes. The residue with β-branching and the residue after it were coupled for double the time. After each coupling, the resin was washed with DMF (5 mL, 5x1 min). The Fmoc-group was then removed by 20% piperidine/DMF (5 mL, 3x8 min). After the final residue was coupled, a solution of acetic anhydride (20 equivalents), DIEA (20 equivalents), and DMF (3 mL) was added to resin for acetyl capping. The reaction was shaken for 2 hours.

Peptides were deprotected and cleaved off the resin by treating the resin with 5 mL 95:5 trifluoroacetic acid (TFA)/triisopropylsilane and shaken for 2 hours. For Cys-containing peptides, 5 mL 90:5:5 trifluoroacetic acid (TFA)/triisopropylsilane/ethanedithiol was used instead. The solution was then filtered through glass wool and the resin was washed with TFA (3x1.5 mL). The combined filtrate was evaporated by a gentle stream of air (nitrogen gas was used for the Cys-containing peptides). The resulting material was washed with hexanes (3x3 mL), dissolved in water, and lyophilized. The peptide (1 mg/ mL, aqueous solution) was analyzed using analytical RP-HPLC on a 25 cm C_{18} column (dia 4.6 mm) with flow rate 1 mL/min, temperature 25°C, linear 1 %/ min gradient from 100% A to 0% A (solvent A: 99.9% water, 0.1% TFA; solvent B: 90%

acetonitrile, 10% water, 0.1% TFA). The disulfide bond of the Cys-containing HPDFZbbAgx peptides were formed via charcoal mediated air oxidation [3]. Peptides were purified to higher than 95% purity by Sep-Pak® Plus Short tC18 cartridges using an appropriate percentage of B solvent and by reverse phase HPLC using a preparative C_{18} column with flow rate 10 mL·min^{-1}, temperature 25°C, linear 0.5 %·min^{-1} gradient. Appropriate linear gradients of solvent A and solvent B were used for each peptide to place the retention time for the desired peptide between 20 and 30 minutes. These gradients are listed individually for each peptide (vide infra); for example, PLG18_28 was used to purify HPDAspAgp using a C_{18} column, representing the linear gradient from 18 % B to 28 % B (flow rate 10 mL·min^{-1}, temperature 25°C, linear 0.5 %·min^{-1} gradient). The identity of the peptide was confirmed by MALDI-TOF.

HPDAspAgp

(Ac-Arg-Asp-Val-Thr-Val-^{D}Pro-Gly-Orn-Agp-Ile-Leu-Gln-NH_2)

The peptide was synthesized using 532.6 mg (0.133 mmol) of NovaSyn®TGR resin. The synthesis gave 721.1 mg of resin (68.1% yield). The cleavage yielded 141.4 mg of crude peptide (98.5% yield). Retention time on the analytical reverse phase HPLC was 28.2 minutes. The peptide was purified by a Sep-Pak® Plus Short tC18 cartridge (30% B solvent) and preparative RP-HPLC using a C18 (PLG18_28) column to 95.5% purity (1.7 mg). The identity of the peptide was confirmed by MALDI-TOF mass spectrometry. Calculated for $C_{59}H_{105}N_{21}O_{17}$ [MH^+]: 1380.807; observed: 1381.120. The concentration of peptide for NMR analysis was 2.5 mM.

HPDGluAgp

(Ac-Arg-Glu-Val-Thr-Val-^{D}Pro-Gly-Orn-Agp-Ile-Leu-Gln-NH_2)

The peptide was synthesized using 214.1 mg (0.054 mmol) of NovaSyn®TGR resin. The synthesis gave 302.3 mg of resin (74.9% yield). The cleavage yielded 55.8 mg of crude peptide (80.9% yield). Retention time on the analytical reverse phase HPLC was 27.7 minutes. The peptide was purified by a Sep-Pak® Plus Short tC18 cartridge (30% B solvent) and preparative RP-HPLC using a C18 (PLG17_27) column to 95.0% purity (2.8 mg). The identity of the peptide was confirmed by MALDI-TOF mass spectrometry. Calculated for $C_{60}H_{107}N_{21}O_{17}$ [MH^+]: 1394.823; observed: 1394.948. The concentration of peptide for NMR analysis was 4.0 mM.

HPDAadAgp

(Ac-Arg-Aad-Val-Thr-Val-^{D}Pro-Gly-Orn-Agp-Ile-Leu-Gln-NH_2)

The peptide was synthesized using 222.4 mg (0.056 mmol) of NovaSyn®TGR resin. The synthesis gave 288.9 mg of resin (53.7% yield). The cleavage yielded 74.6 mg of crude peptide (>99% yield). Retention time on the analytical reverse phase HPLC was 28.0 minutes. The peptide was purified by a Sep-Pak® Plus Short tC18 cartridge and preparative RP-HPLC using a C18 (PLG18_28) column to 95.2% purity (4.4 mg). The identity of the peptide was confirmed by MALDI-TOF mass spectrometry. Calculated for $C_{61}H_{109}N_{21}O_{17}$ [MH^+]: 1408.838; observed:

1409.090. The concentration of peptide for NMR analysis was 6.2 mM.

HPDAspAgb

(Ac-Arg-Asp-Val-Thr-Val-^{D}Pro-Gly-Orn-Agb-Ile-Leu-Gln-NH_2)

The peptide was synthesized using 210.9 mg (0.051 mmol) of NovaSyn®TGR resin. The synthesis gave 306.0 mg of resin (92.0% yield). The cleavage yielded 80.8 mg of crude peptide (94.1% yield). Retention time on the analytical reverse phase HPLC was 28.4 minutes. The peptide was purified by preparative RP-HPLC using a C4 (PLG8_18) column and a C18 (PLG17_27) column to 95.3% purity (3.1 mg). The identity of the peptide was confirmed by MALDI-TOF mass spectrometry. Calculated for $C_{60}H_{107}N_{21}O_{17}$ [MH^+]: 1394.823; observed: 1394.972. The concentration of peptide for NMR analysis was 4.4 mM.

HPDGluAgb

(Ac-Arg-Glu-Val-Thr-Val-^{D}Pro-Gly-Orn-Agb-Ile-Leu-Gln-NH_2)

The peptide was synthesized using 219.5 mg (0.055 mmol) of NovaSyn®TGR resin. The synthesis gave 326.0 mg of resin (87.1% yield). The cleavage yielded 62.5 mg of crude peptide (73.3% yield). Retention time on the analytical reverse phase HPLC was 27.2 minutes. The peptide was purified by preparative RP-HPLC using a C4 (PLG7_17) column and a C18 (PLG17_27) column to 95.2% purity (8.1 mg). The identity of the peptide was confirmed by MALDI-TOF mass spectrometry. Calculated for $C_{61}H_{109}N_{21}O_{17}$ [MH^+]: 1408.838; observed: 1409.145. The concentration of peptide for NMR analysis was 7.7 mM.

HPDAadAgb

(Ac-Arg-Aad-Val-Thr-Val-^{D}Pro-Gly-Orn-Agb-Ile-Leu-Gln-NH_2)

The peptide was synthesized using 243.5 mg (0.061 mmol) of NovaSyn®TGR resin. The synthesis gave 323.1 mg of resin (58.6% yield). The cleavage yielded 58.2 mg of crude peptide (99.7% yield). Retention time on the analytical reverse phase HPLC was 26.9 minutes. The peptide was purified by a Sep-Pak® Plus Short tC18 cartridge and preparative RP-HPLC using a C18 (PLG17_27) column to 95.8% purity (10.6 mg). The identity of the peptide was confirmed by MALDI-TOF mass spectrometry. Calculated for $C_{62}H_{111}N_{21}O_{17}$ [MH^+]: 1422.854; observed: 1423.090. The concentration of peptide for NMR analysis was 8.8 mM.

HPDAspArg

(Ac-Arg-Asp-Val-Thr-Val-^{D}Pro-Gly-Orn-Arg-Ile-Leu-Gln-NH_2)

The peptide was synthesized using 212.0 mg (0.053 mmol) of NovaSyn®TGR resin. The synthesis gave 327.9 mg of resin (>99% yield). The cleavage yielded 49.3 mg of crude peptide (52.2% yield). Retention time on the analytical reverse phase HPLC was 28.5 minutes. The peptide was purified by a Sep-Pak® Plus Short tC18 cartridge (30% B solvent) and preparative RP-HPLC using a C18 (PLG17_27) column to 98.2% purity (12.3 mg). The identity of the peptide was confirmed by MALDI-TOF mass spectrometry. Calculated for $C_{61}H_{109}N_{21}O_{17}$ [MH^+]: 1408.838;

observed: 1408.717. The concentration of peptide for NMR analysis was 8.73 mM.

HPDGluArg

(Ac-Arg-Glu-Val-Thr-Val-^{D}Pro-Gly-Orn-Arg-Ile-Leu-Gln-NH$_2$)

The peptide was synthesized using 201.3 mg (0.050 mmol) of NovaSyn®TGR resin. The synthesis gave 306.2 mg of resin (97.3% yield). The cleavage yielded 87.1 mg of crude peptide (>99% yield). Retention time on the analytical reverse phase HPLC was 27.7 minutes. The peptide was purified by preparative RP-HPLC using a C4 (PLG8_18) column and a C18 (PLG16_26) column to 95.3% purity (9.5 mg). The identity of the peptide was confirmed by MALDI-TOF mass spectrometry. Calculated for $C_{62}H_{111}N_{21}O_{17}$ [MH$^+$]: 1422.854; observed: 1422.939. The concentration of peptide for NMR analysis was 8.3 mM.

HPDAadArg

(Ac-Arg-Aad-Val-Thr-Val-^{D}Pro-Gly-Orn-Arg-Ile-Leu-Gln-NH$_2$)

The peptide was synthesized using 206.7 mg (0.052 mmol) of NovaSyn®TGR resin. The synthesis gave 327.7 mg of resin (>99% yield). The cleavage yielded 77.1 mg of crude peptide (85.4% yield). Retention time on the analytical reverse phase HPLC was 27.8 minutes. The peptide was purified by a Sep-Pak® Plus Short tC18 cartridge (30% B solvent) and preparative RP-HPLC using a C18 (PLG16_26) column to 98.2% purity (19.0 mg). The identity of the peptide was confirmed by MALDI-TOF mass spectrometry. Calculated for $C_{63}H_{113}N_{21}O_{17}$ [MH$^+$]: 1436.870; observed: 1436.974. The concentration of peptide for NMR analysis was 8.82 mM.

HPDAspAgh

(Ac-Arg-Asp-Val-Thr-Val-^{D}Pro-Gly-Orn-Agh-Ile-Leu-Gln-NH$_2$)

The peptide was synthesized using 204.2 mg (0.051 mmol) of NovaSyn®TGR resin. The synthesis gave 288.5 mg of resin (79.0% yield). The cleavage yielded 74.3 mg of crude peptide (>99% yield). Retention time on the analytical reverse phase HPLC was 28.6 minutes. The peptide was purified by preparative RP-HPCL using a C4 (PLG8_18) column and a C18 (PLG17_27) column to 95.3% purity (7.4 mg). The identity of the peptide was confirmed by MALDI-TOF mass spectrometry. Calculated for $C_{62}H_{111}N_{21}O_{17}$ [MH$^+$]: 1422.854; observed: 1422.936. The concentration of peptide for NMR analysis was 8.4 mM.

HPDGluAgh

(Ac-Arg-Glu-Val-Thr-Val-^{D}Pro-Gly-Orn-Agh-Ile-Leu-Gln-NH$_2$)

The peptide was synthesized using 202.1 mg (0.051 mmol) of NovaSyn®TGR resin. The synthesis gave 280.7 mg of resin (73.6% yield). The cleavage yielded 54.0 mg of crude peptide (81% yield). Retention time on the analytical reverse phase HPLC was 27.9 minutes. The peptide was purified by preparative RP-HPCL using a C4 (PLG7_17) and a C18 (PLG16_26) column to 95.4% purity (9.2 mg). The identity of the peptide was confirmed by MALDI-TOF mass spectrometry. Calculated for $C_{63}H_{113}N_{21}O_{17}$ [MH$^+$]: 1436.870; observed: 1436.968. The

concentration of peptide for NMR analysis was 8.5 mM.

HPDAadAgh

(Ac-Arg-Aad-Val-Thr-Val-^{D}Pro-Gly-Orn-Agh-Ile-Leu-Gln-NH_2)

The peptide was synthesized using 204.5 mg (0.051 mmol) of NovaSyn®TGR resin. The synthesis gave 285.1 mg of resin (74.4% yield). The cleavage yielded 76.5 mg of crude peptide (48.8% yield). Retention time on the analytical reverse phase HPLC was 27.9 minutes. The peptide was purified by preparative RP-HPLC using a C4 (PLG8_18) column to 95.2% purity (11.0 mg). The identity of the peptide was confirmed by MALDI-TOF mass spectrometry. Calculated for $C_{64}H_{115}N_{21}O_{17}$ [MH^+]: 1450.885; observed: 1450.836. The concentration of peptide for NMR analysis was 7.58 mM.

HPDUAspAgp

(Ac-Arg-Asp-Val-Thr-Val-^{L}Pro-Gly-Orn-Agp-Ile-Leu-Gln-NH_2)

The peptide was synthesized using 224.4 mg (0.056 mmol) of NovaSyn®TGR resin. The synthesis gave 330.0 mg of resin (85.5% yield). The cleavage yielded 62.2 mg of crude peptide (74.4% yield). Retention time on the analytical reverse phase HPLC was 25.6 minutes. The peptide was purified by a Sep-Pak® Plus Short tC18 cartridge (30% B solvent) and preparative RP-HPLC using a C18 (PLG16_26) column to 96.5% purity (3.0 mg). The identity of the peptide was confirmed by MALDI-TOF mass spectrometry. Calculated for $C_{59}H_{105}N_{21}O_{17}$ [MH^+]: 1380.807; observed: 1381.036. The concentration of peptide for NMR analysis was 4.3 mM.

HPDUGluAgp

(Ac-Arg-Glu-Val-Thr-Val-^{L}Pro-Gly-Orn-Agp-Ile-Leu-Gln-NH_2)

The peptide was synthesized using 247.0 mg (0.062 mmol) of NovaSyn®TGR resin. The synthesis gave 338.4 mg of resin (66.8% yield). The cleavage yielded 78.8 mg of crude peptide (>99% yield). Retention time on the analytical reverse phase HPLC was 25.6 minutes. The peptide was purified by a Sep-Pak® Plus Short tC18 cartridge (30% B solvent) and preparative RP-HPLC using a C18 (PLG16_26) column to 96.1% purity (1.9 mg). The identity of the peptide was confirmed by MALDI-TOF mass spectrometry. Calculated for $C_{60}H_{107}N_{21}O_{17}$ [MH^+]: 1394.823; observed: 1394.948. The concentration of peptide for NMR analysis was 2.7 mM.

HPDUAadAgp

(Ac-Arg-Aad-Val-Thr-Val-^{L}Pro-Gly-Orn-Agp-Ile-Leu-Gln-NH_2)

The peptide was synthesized using 224.0 mg (0.056 mmol) of NovaSyn®TGR resin. The synthesis gave 295.9 mg of resin (57.6% yield). The cleavage yielded 71.5 mg of crude peptide (>99% yield). Retention time on the analytical reverse phase HPLC was 26.1 minutes. The peptide was purified by a Sep-Pak® Plus Short tC18 cartridge (30% B solvent) and preparative RP-HPLC using a C18 (PLG16_26) column to 95.6% purity (3.2 mg). The identity of the peptide was

confirmed by MALDI-TOF mass spectrometry. Calculated for $C_{61}H_{109}N_{21}O_{17}$ [MH$^+$]: 1408.838; observed: 1409.059. The concentration of peptide for NMR analysis was 4.5 mM.

HPDUAspAgb

(Ac-Arg-Asp-Val-Thr-Val-^{L}Pro-Gly-Orn-Agb-Ile-Leu-Gln-NH$_2$)

The corresponding Dab(ivDde)-containing peptide (Ac-Arg-Asp-Val-Thr-Val-^{L}Pro-Gly-Orn-Dab(ivDde)-Ile-Leu-Gln-NH$_2$) was synthesized using 200.7 mg (0.050 mmole) of NovaSyn®TGR resin. Retention time on the analytical reverse phase HPLC was 37.1 minutes. The identity of peptide was confirmed by MALDI-TOF mass spectrometry. Calculated for $C_{77}H_{123}N_{19}O_{19}$ [MH$^+$]: 1558.932; observed: 1559.436. The (4,4-dimethyl-2,6-dioxocyclohexylidene)-3-methylbutyl (ivDde) group on the protected HPDUAspDab(ivDde) bound resin was removed selectively using 2% hydrazine in DMF (2 mL, 10 × 8 min) under microwave conditions (power 240 W, 10 × 20 sec). Then, 6% hydrazine in NMP (2 mL, 10 × 8 min) under microwave conditions (power 240 W, 10 × 20 sec) was used to cleave the ivDde group completely. The resin was reacted with a solution of N,N'-di-Boc-N"-trifluoromethanesulfonylguanidine (282.5 mg, 0.72 mmole), N,N-diisopropylethyl- amine (240 μL), and DCM (1.5 mL) under microwave conditions (power 240 W, 2000 × 20 sec). Retention time of the guanidinylated product on the analytical reverse phase HPLC was 25.9 minutes. The synthesis gave 279.0 mg of resin (75.7% yield). The cleavage yielded 54.3 mg of crude peptide (69.2% yield). The peptide was purified by a Sep-Pak® Plus Short tC18 cartridge (25% B solvent) and preparative RP-HPLC using a C18 (PLG15_25) column to 96.1% purity (2.1 mg). The identity of the peptide was confirmed by MALDI-TOF mass spectrometry. Calculated for $C_{60}H_{107}N_{21}O_{17}$ [MH$^+$]: 1394.823; observed: 1394.858. The concentration of peptide for NMR analysis was 3.0 mM.

HPDUGluAgb

(Ac-Arg-Glu-Val-Thr-Val-^{L}Pro-Gly-Orn-Agb-Ile-Leu-Gln-NH$_2$)

The peptide was synthesized using 245.8 mg (0.061 mmol) of NovaSyn®TGR resin. The synthesis gave 340.6mg of resin (69.2% yield). The cleavage yielded 58.7 mg of crude peptide (78.7% yield). Retention time on the analytical reverse phase HPLC was 25.5 minutes. The peptide was purified by preparative RP-HPLC using a C4 (PLG5_15) column to 96.1% purity (11.1 mg). The identity of the peptide was confirmed by MALDI-TOF mass spectrometry. Calculated for $C_{61}H_{109}N_{21}O_{17}$ [MH$^+$]: 1408.838; observed: 1409.037. The concentration of peptide for NMR analysis was 7.88 mM.

HPDUAadAgb

(Ac-Arg-Aad-Val-Thr-Val-^{L}Pro-Gly-Orn-Agb-Ile-Leu-Gln-NH$_2$)

The peptide was synthesized using 243.7 mg (0.061 mmol) of NovaSyn®TGR resin. The synthesis gave 332.9 mg of resin (65.3% yield). The cleavage yielded 69.4 mg of crude peptide (>99% yield). Retention time on the analytical reverse phase HPLC was 25.9 minutes. The peptide

was purified by preparative RP-HPLC using a C4 (PLG5_15) column and a C18 (PLG16_26 column) to 97.1% purity (9.6 mg). The identity of the peptide was confirmed by MALDI-TOF mass spectrometry. Calculated for $C_{62}H_{111}N_{21}O_{17}$ [MH^+]: 1422.854; observed: 1422.759. The concentration of peptide for NMR analysis was 8.4 mM.

HPDUAspArg

(Ac-Arg-Asp-Val-Thr-Val-^{L}Pro-Gly-Orn-Arg-Ile-Leu-Gln-NH_2)

The peptide was synthesized using 216.7 mg (0.054 mmol) of NovaSyn®TGR resin. The synthesis gave 322.9 mg of resin (92.1% yield). The cleavage yielded 67.8 mg of crude peptide (78.2% yield). Retention time on the analytical reverse phase HPLC was 26.0 minutes. The peptide was purified by preparative RP-HPLC using a C4 (PLG5_15) column and a C18 (PLG15_25) column to 97.1% purity (14.3 mg). The identity of the peptide was confirmed by MALDI-TOF mass spectrometry. Calculated for $C_{61}H_{109}N_{21}O_{17}$ [MH^+]: 1408.838; observed: 1408.767. The concentration of peptide for NMR analysis was 8.5 mM.

HPDUGluArg

(Ac-Arg-Glu-Val-Thr-Val-^{L}Pro-Gly-Orn-Arg-Ile-Leu-Gln-NH_2)

The peptide was synthesized using 200.9 mg (0.050 mmol) of NovaSyn®TGR resin. The synthesis gave 370.1 mg of resin (>99% yield). The cleavage yielded 64.9 mg of crude peptide (47.4% yield). Retention time on the analytical reverse phase HPLC was 25.9 minutes. The peptide was purified by preparative RP-HPLC using a C4 (PLG5_15) column to 96.0% purity (17.1 mg). The identity of the peptide was confirmed by MALDI-TOF mass spectrometry. Calculated for $C_{62}H_{111}N_{21}O_{17}$ [MH^+]: 1422.854; observed: 1422.883. The concentration of peptide for NMR analysis was 6.01 mM.

HPDUAadArg

(Ac-Arg-Aad-Val-Thr-Val-^{L}Pro-Gly-Orn-Arg-Ile-Leu-Gln-NH_2)

The peptide was synthesized using 206.7 mg (0.052 mmol) of NovaSyn®TGR resin. The synthesis gave 308.8 mg of resin (91.6% yield). The cleavage yielded 60.6 mg of crude peptide (79.4% yield). Retention time on the analytical reverse phase HPLC was 26.5 minutes. The peptide was purified by a Sep-Pak® Plus Short tC18 cartridge (30% B solvent) and preparative RP-HPLC using a C18 (PLG15_25) column to 97.3% purity (11.4 mg). The identity of the peptide was confirmed by MALDI-TOF mass spectrometry. Calculated for $C_{63}H_{113}N_{21}O_{17}$ [MH^+]: 1436.870; observed: 1436.958. The concentration of peptide for NMR analysis was 7.93 mM.

HPDUAspAgh

(Ac-Arg-Asp-Val-Thr-Val-^{L}Pro-Gly-Orn-Agh-Ile-Leu-Gln-NH_2)

The peptide was synthesized using 200.6 mg (0.050 mmol) of NovaSyn®TGR resin. The synthesis gave 290.7 mg of resin (85.9% yield). The cleavage yielded 75.0 mg of crude peptide (88.0% yield). Retention time on the analytical reverse phase HPLC was 26.3 minutes. The peptide

was purified by a Sep-Pak® Plus Short tC18 cartridge (30% B solvent) and preparative RP-HPLC using a C18 (PLG15_25) column to 95.2% purity (5.8 mg). The identity of the peptide was confirmed by MALDI-TOF mass spectrometry. Calculated for $C_{62}H_{111}N_{21}O_{17}$ [MH^+]: 1422.854; observed: 1422.973. The concentration of peptide for NMR analysis was 8.5 mM.

HPDUGluAgh

(Ac-Arg-Glu-Val-Thr-Val-^{L}Pro-Gly-Orn-Agh-Ile-Leu-Gln-NH_2)

The peptide was synthesized using 204.1 mg (0.051 mmol) of NovaSyn®TGR resin. The synthesis gave 295.5 mg of resin (85.1% yield). The cleavage yielded 46.2 mg of crude peptide (46.2% yield). Retention time on the analytical reverse phase HPLC was 26.3 minutes. The peptide was purified by preparative RP-HPLC using a C4 (PLG4_14) column to 95.4% purity (9.8 mg). The identity of the peptide was confirmed by MALDI-TOF mass spectrometry. Calculated for $C_{63}H_{113}N_{21}O_{17}$ [MH^+]: 1436.870; observed: 1436.963. The concentration of peptide for NMR analysis was 8.5 mM.

HPDUAadAgh

(Ac-Arg-Aad-Val-Thr-Val-^{L}Pro-Gly-Orn-Agh-Ile-Leu-Gln-NH_2)

The peptide was synthesized using 200.7 mg (0.050 mmol) of NovaSyn®TGR resin. The synthesis gave 286.3 mg of resin (80.5% yield). The cleavage yielded 56.2 mg of crude peptide (83.8% yield). Retention time on the analytical reverse phase HPLC was 27.9 minutes. The peptide was purified by preparative RP-HPLC using a C4 (PLG5_15) column to 95.5% purity (14.0 mg). The identity of the peptide was confirmed by MALDI-TOF mass spectrometry. Calculated for $C_{64}H_{115}N_{21}O_{17}$ [MH^+]: 1450.885; observed: 1450.854. The concentration of peptide for NMR analysis was 8.04 mM.

HPDFAspAgp

(Ac-Cys-Arg-Asp-Val-Thr-Val-^{D}Pro-Gly-Orn-Agp-Ile-Leu-Gln-Cys-NH_2[O])

The peptide was synthesized using 251.2 mg (0.063 mmol) of NovaSyn®TGR resin. The synthesis gave 347.2 mg of resin (52.9% yield). The cleavage yielded 59.1 mg of crude peptide (91.0% yield). Retention time on the analytical RP-HPLC was 30.1 minutes. Calculated for $C_{65}H_{115}N_{23}O_{19}C_{64}S_2$ [MH^+]: 1586.825; observed: 1586.993. The crude peptide was dissolved in 1 mM pH 8 phosphate, citrate and borate buffer (60 mL). Granulated charcoal (577.3 mg) was added to the peptide solution [3]. After stirring over air for 2.5 hours, the cyclized peptide was purified by a Sep-Pak® Plus Short tC18 cartridge (30% B solvent) and preparative RP-HPLC using a C18 (PLG17_27) column to 95.7% purity (2.5 mg). The retention time of the cyclized peptide on the analytical RP-HPLC was 27.2 minutes. The identity of the peptide was confirmed by MALDI-TOF mass spectrometry. Calculated for $C_{65}H_{113}N_{23}O_{19}C_{64}S_2$ [MH^+]: 1584.810; observed: 1584.875. The concentration of peptide for NMR analysis was 3.2 mM.

HPDFGluAgp

(Ac-Cys-Arg-Glu-Val-Thr-Val-^{D}Pro-Gly-Orn-Agp-Ile-Leu-Gln-Cys-NH_2[O])

The peptide was synthesized using 257.2 mg (0.064 mmol) of NovaSyn®TGR resin. The synthesis gave 357.4 mg of resin (53.6% yield). The cleavage yielded 60.5 mg of crude peptide (87.6% yield). Retention time on the analytical RP-HPLC was 29.8 minutes. Calculated for $C_{66}H_{117}N_{23}O_{19}S_2$ [MH^+]: 1600.841; observed: 1601.022. The crude peptide was dissolved in 1 mM pH 8 phosphate, citrate and borate buffer (80 mL). Granulated charcoal (572.4 mg) was added to the peptide solution [3]. After stirring over air for 2.5 hours, the cyclized peptide was purified by preparative RP-HPLC using a C4 (PLG11_21) column and a C18 (PLG17_27) column to 95.4% purity (1.1 mg). The retention time of the cyclized peptide on the analytical RP-HPLC was 27.3 minutes. The identity of the peptide was confirmed by MALDI-TOF mass spectrometry. Calculated for $C_{66}H_{115}N_{23}O_{19}S_2$ [MH^+]: 1598.825; observed: 1598.743. The concentration of peptide for NMR analysis was 1.4 mM.

HPDFAadAgp

(Ac-Cys-Arg-Aad-Val-Thr-Val-^{D}Pro-Gly-Orn-Agp-Ile-Leu-Gln-Cys-NH_2[O])

The peptide was synthesized using 255.5 mg (0.064 mmol) of NovaSyn®TGR resin. The synthesis gave 361.0 mg of resin (56.6% yield). The cleavage yielded 63.7 mg of crude peptide (96.9% yield). Retention time on the analytical RP-HPLC was 30.0 minutes. Calculated for $C_{67}H_{119}N_{23}O_{19}S_2$ [MH^+]: 1614.857; observed: 1615.118. The crude peptide was dissolved in 1 mM pH 8 phosphate, citrate and borate buffer (80 mL). Granulated charcoal (626.8 mg) was added to the peptide solution [3]. After stirring over air for 3 hours, the cyclized peptide was purified by a Sep-Pak® Plus Short tC18 cartiridge (30% B solvent) and preparative RP-HPLC using a C18 (PLG18_28) column to 95.2% purity (2.6 mg). The retention time of the cyclized peptide on the analytical RP-HPLC was 27.6 minutes. The identity of the peptide was confirmed by MALDI-TOF mass spectrometry. Calculated for $C_{67}H_{117}N_{23}O_{19}S_2$ [MH^+]: 1612.841; observed: 1613.200. The concentration of peptide for NMR analysis was 3.2 mM.

HPDFAspAgb

(Ac-Cys-Arg-Asp-Val-Thr-Val-^{D}Pro-Gly-Orn-Agb-Ile-Leu-Gln-Cys-NH_2[O])

The peptide was synthesized using 252.7 mg (0.063 mmol) of NovaSyn®TGR resin. The synthesis gave 374.0 mg of resin (66.1% yield). The cleavage yielded 110.3 mg of crude peptide (>99% yield). Retention time on the analytical reverse phase HPLC was 30.2 minutes. Calculated for $C_{66}H_{117}N_{23}O_{19}S_2$ [MH^+]: 1600.841; observed: 1601.116. The crude peptide was dissolved in 1 mM pH 8 phosphate, citrate and borate buffer (120 mL). Granulated charcoal (1063.4 mg) was added to the peptide solution [3]. After stirring over air for 3 hours, the cyclized peptide was purified by preparative RP-HPLC using a C4 (PLG9_19) column to 95.4% purity (3.1 mg). The retention time of the cyclized peptide on the analytical RP-HPLC was 26.5 minutes. The identity of the peptide was confirmed by MALDI-TOF mass spectrometry. Calculated for $C_{66}H_{115}N_{23}O_{19}S_2$ [MH^+]: 1598.825; observed: 1598.740. The concentration of peptide for NMR analysis was 3.9

mM.

HPDFGluAgb

(Ac-Cys-Arg-Glu-Val-Thr-Val-^{D}Pro-Gly-Orn-Agb-Ile-Leu-Gln-Cys-NH_2[O])

The peptide was synthesized using 265.3 mg (0.063 mmol) of NovaSyn®TGR resin. The synthesis gave 386.6 mg of resin (62.6% yield). The cleavage yielded 78.0 mg of crude peptide (93.4% yield). Retention time on the analytical reverse phase HPLC was 29.3 minutes. Calculated for $C_{67}H_{119}N_{23}O_{19}S_2$ [MH^+]: 1614.857; observed: 1615.084. The crude peptide was dissolved in 1 mM pH 8 phosphate, citrate and borate buffer (90 mL). Granulated charcoal (752.0 mg) was added to the peptide solution [3]. After stirring over air for 2 hours, the cyclized peptide was purified by preparative RP-HPLC using a C4 (PLG9_19) column to 95.0% purity (2.7 mg). The retention time of the cyclized peptide on the analytical RP-HPLC was 26.4 minutes. The identity of the peptide was confirmed by MALDI-TOF mass spectrometry. Calculated for $C_{67}H_{117}N_{23}O_{19}S_2$ [MH^+]: 1612.841; observed: 1612.873. The concentration of peptide for NMR analysis was 3.3 mM.

HPDFAadAgb

(Ac-Cys-Arg-Aad-Val-Thr-Val-^{D}Pro-Gly-Orn-Agb-Ile-Leu-Gln-Cys-NH_2[O])

The peptide was synthesized using 251.0 mg (0.063 mmol) of NovaSyn®TGR resin. The synthesis gave 370.9 mg of resin (65.1% yield). The cleavage yielded 86.5 mg of crude peptide (>99% yield). Retention time on the analytical reverse phase HPLC was 29.4 minutes. Calculated for $C_{68}H_{121}N_{23}O_{19}S_2$ [MH^+]: 1628.872; observed: 1629.138. The crude peptide was dissolved in 1 mM pH 8 phosphate, citrate and borate buffer (100 mL). Granulated charcoal (841.4 mg) was added to the peptide solution [3]. After stirring over air for 2.5 hours, the cyclized peptide was purified by preparative RP-HPLC using a C4 (PLG9_19) column and a C18 (PLG16_26) column to 96.4% purity (1.9 mg). The retention time of the cyclized peptide on the analytical RP-HPLC was 26.7 minutes. The identity of the peptide was confirmed by MALDI-TOF mass spectrometry. Calculated for $C_{68}H_{119}N_{23}O_{19}S_2$ [MH^+]: 1626.857; observed: 1627.116. The concentration of peptide for NMR analysis was 2.3 mM.

HPDFAspArg

(Ac-Cys-Arg-Asp-Val-Thr-Val-^{D}Pro-Gly-Orn-Arg-Ile-Leu-Gln-Cys-NH_2[O])

The peptide was synthesized using 201.8 mg (0.050 mmol) of NovaSyn®TGR resin. The synthesis gave 340.7 mg of resin (97.6% yield). The cleavage yielded 86.4 mg of crude peptide (85.6% yield). Retention time on the analytical reverse phase HPLC was 30.6 minutes. Calculated for $C_{67}H_{119}N_{23}O_{19}S_2$ [MH^+]: 1614.857; observed: 1615.095. The crude peptide was dissolved in 1 mM pH 8 phosphate, citrate and borate buffer (100 mL). Granulated charcoal (812.3 mg) was added to the peptide solution [3]. After stirring over air for 4 hours, the cyclized peptide was purified by preparative RP-HPLC using a C4 (PLG8_18) column to 95.7% purity (7.2 mg). The retention time of the cyclized peptide on the analytical RP-HPLC was 26.8 minutes. The identity of the peptide was confirmed by MALDI-TOF mass spectrometry. Calculated for $C_{67}H_{117}N_{23}O_{19}S_2$ [MH^+]:

1612.841; observed: 1612.754. The concentration of peptide for NMR analysis was 8.9 mM.

HPDFGluArg

(Ac-Cys-Arg-Glu-Val-Thr-Val-^{D}Pro-Gly-Orn-Arg-Ile-Leu-Gln-Cys-NH$_2$[O])

The peptide was synthesized using 200.5 mg (0.050 mmol) of NovaSyn®TGR resin. The synthesis gave 320.8 mg of resin (83.2% yield). The cleavage yielded 87.1 mg of crude peptide (>99%). Retention time on the analytical RP-HPLC was 29.9 minutes. Calculated for $C_{68}H_{121}N_{23}O_{19}S_2$ [MH$^+$]: 1628.872; observed: 1629.085. The crude peptide was dissolved in 1 mM pH 8 phosphate, citrate and borate buffer (100 mL). Granulated charcoal (1204.3 mg) was added to the peptide solution [3]. After stirring over air for 4 hours, the cyclized peptide was purified by preparative RP-HPLC using a C4 (PLG8_18) column and a C18 (PLG16_26) column to 95.5% purity (4.7 mg). The retention time of the cyclized peptide on the analytical RP-HPLC was 26.7 minutes. The identity of the peptide was confirmed by MALDI-TOF mass spectrometry. Calculated for $C_{68}H_{119}N_{23}O_{19}S_2$ [MH$^+$]: 1626.897; observed: 1626.805. The concentration of peptide for NMR analysis was 5.8 mM.

HPDFAadArg

(Ac-Cys-Arg-Aad-Val-Thr-Val-^{D}Pro-Gly-Orn-Arg-Ile-Leu-Gln-Cys-NH$_2$[O])

The peptide was synthesized using 201.8 mg (0.050 mmol) of NovaSyn®TGR resin. The synthesis gave 336.1 mg of resin (93.5% yield). The cleavage yielded 83.7 mg of crude peptide (97.5% yield). Retention time on the analytical RP-HPLC was 30.3 minutes. Calculated for $C_{69}H_{123}N_{23}O_{19}S_2$ [MH$^+$]: 1642.888; observed: 1643.052. The crude peptide was dissolved in 1 mM pH 8 phosphate, citrate and borate buffer (100 mL). Granulated charcoal (848.5 mg) was added to the peptide solution [3]. After stirring over air for 4 hours, the cyclized peptide was purified by preparative RP-HPLC using a C4 (PLG8_18) column to 95.7% purity (7.1 mg). The retention time of the cyclized peptide on the analytical RP-HPLC was 26.8 minutes. The identity of the peptide was confirmed by MALDI-TOF mass spectrometry. Calculated for $C_{69}H_{121}N_{23}O_{19}S_2$ [MH$^+$]: 1640.872; observed: 1640.890. The concentration of peptide for NMR analysis was 8.7 mM.

HPDFAspAgh

(Ac-Cys-Arg-Asp-Val-Thr-Val-^{D}Pro-Gly-Orn-Agh-Ile-Leu-Gln-Cys-NH$_2$[O])

The peptide was synthesized using 201.9 mg (0.050 mmol) of NovaSyn®TGR resin. The synthesis gave 309.3 mg of resin (76.5% yield). The cleavage yielded 79.6 mg of crude peptide (94.5% yield). Retention time on the analytical RP-HPLC was 30.9 minutes. Calculated for $C_{68}H_{117}N_{23}O_{19}S_2$ [MH$^+$]: 1628.872; observed: 1629.023. The crude peptide was dissolved in 1 mM pH 8 phosphate, citrate and borate buffer (90 mL). Granulated charcoal (669.0 mg) was added to the peptide solution [3]. After stirring over air for 4 hours, the cyclized peptide was purified by preparative RP-HPLC using a C4 (PLG8_18) column to 95.9% purity (4.1 mg). The retention time of the cyclized peptide on the analytical RP-HPLC was 27.4 minutes. The identity of the peptide was confirmed by MALDI-TOF mass spectrometry. Calculated for $C_{68}H_{119}N_{23}O_{19}S_2$ [MH$^+$]:

1626.857; observed: 1627.035. The concentration of peptide for NMR analysis was 5.6 mM.

HPDFGluAgh

(Ac-Cys-Arg-Glu-Val-Thr-Val-^{D}Pro-Gly-Orn-Agh-Ile-Leu-Gln-Cys-NH_2[O])

The peptide was synthesized using 204.6 mg (0.051 mmol) of NovaSyn®TGR resin. The synthesis gave 313.0 mg of resin (75.8% yield). The cleavage yielded 95.0 mg of crude peptide (>99%). Retention time on the analytical RP-HPLC was 30.3 minutes. Calculated for $C_{69}H_{119}N_{23}O_{19}S_2$ [MH^+]: 1642.888; observed: 1643.075. The crude peptide was dissolved in 1 mM pH 8 phosphate, citrate and borate buffer (100 mL). Granulated charcoal (856.0 mg) was added to the peptide solution [3]. After stirring over air for 2 hours, the cyclized peptide was purified by preparative RP-HPLC using a C4 (PLG8_18) column to 95.3% purity (4.7 mg). The retention time of the cyclized peptide on the analytical RP-HPLC was 27.2 minutes. The identity of the peptide was confirmed by MALDI-TOF mass spectrometry. Calculated for $C_{69}H_{121}N_{23}O_{19}S_2$ [MH^+]: 1640.872; observed: 1641.028. The concentration of peptide for NMR analysis was 4.1 mM.

HPDFAadAgh

(Ac-Cys-Arg-Aad-Val-Thr-Val-^{D}Pro-Gly-Orn-Agh-Ile-Leu-Gln-Cys-NH_2[O])

The peptide was synthesized using 207.2 mg (0.052 mmol) of NovaSyn®TGR resin. The synthesis gave 319.1 mg of resin (76.9% yield). The cleavage yielded 88.6 mg of crude peptide (>99% yield). Retention time on the analytical reverse phase HPLC was 30.0 minutes. Calculated for $C_{70}H_{121}N_{23}O_{19}S_2$ [MH^+]: 1656.904; observed: 1657.130. The crude peptide was dissolved in 1 mM pH 8 phosphate, citrate and borate buffer (100 mL). Granulated charcoal (886.2 mg) was added to the peptide solution [3]. After stirring over air for 4 hours, the cyclized peptide was purified by preparative RP-HPLC using a C4 (PLG9_19) column to 95.8% purity (4.6 mg). The retention time of the cyclized peptide on the analytical RP-HPLC was 27.3 minutes. The identity of the peptide was confirmed by MALDI-TOF mass spectrometry. Calculated for $C_{70}H_{123}N_{23}O_{19}S_2$ [MH^+]: 1654.888; observed: 1654.935. The concentration of peptide for NMR analysis was 5.6 mM.

NMR Spectroscopy

NMR samples were prepared by dissolving lyophilized purified peptides (purity greater than 95%) in H_2O/D_2O (9:1 ratio by volume), 50 mM sodium deuterioacetate buffer at pH 5.5 (uncorrected). Concentrations of all peptides were 1.4-8.9 mM. 2,2-Dimethyl-2-silapentane-5-sulfonate (DSS) was added to the samples as an internal reference. NMR experiments were performed on a Bruker AV III 800 MHz spectrometer at 298K. Phase-sensitive TOCSY [4], DQF-COSY [5], and ROESY [6] experiments were performed by collecting 2048 points in *f*2 and 256-512 points in *f*1. Solvent suppression was achieved by the WATERGATE solvent suppression sequence [7, 8]. TOCSY and ROESY experiments employed a spin locking field of 10 kHz. Mixing times of 60 msec and 200 msec were used for the TOCSY and ROESY experiments, respectively. The specific sequence assignment was performed based on TOCSY, DQF-COSY, and ROESY data.

$^{3}J_{NH\alpha}$ Coupling Constants

The $^{3}J_{NH\alpha}$ spin-spin coupling constant for the residues in the peptides was derived from the DQF-COSY spectra. The absorptive (ν_a) and dispersive (ν_d) values were obtained along the *f*2 axis. The $^{3}J_{NH\alpha}$ coupling constant was derived from the square root of the single real root of equation 1 [9].

$$J^6 - \nu_d^2 J^4 + \left(-\frac{9}{4}\nu_a^4 + \frac{3}{2}\nu_a^2\nu_d^2 + \frac{3}{4}\nu_d^4\right)J^2 + \frac{81}{64}\nu_a^6 - \frac{9}{16}\nu_a^4\nu_d^2 - \frac{21}{32}\nu_a^2\nu_d^4 - \frac{1}{16}\nu_d^6 + \frac{\nu_d^8}{64\nu_a^2} = 0 \quad \text{(equation 1)}$$

Distance Derivation from NOE Intergration

The NOE cross peaks of all peptides were assigned from the corresponding ROESY spectra. The interproton distances were derived from the NOE signal intensity following equation 2. The integration for the NOE cross peaks was based on a Gaussian peak model. The distance between the two β-hydrogen atoms on the proline side chain in the peptide was set as the standard (1.77 Å) to derived the interproton distance (R).

$$R_{NOE} = 1.77\times10^{-10}\times(\frac{I_{standard}}{I_{NOE}})^{\frac{1}{6}} \quad \text{(equation 2)}$$

The NOEs were grouped into three catagories based on distance: strong NOEs for distances shorter than 2.5 Å, medium NOEs for distances between 2.5 Å and 3.5 Å, and weak NOEs for distances longer than 3.5 Å. The Wüthrich diagrams were used to depict the backbone NOE connectivities involving the α protons and amide protons for the peptides (Figures S39-S50). The strength of the NOEs in these diagrams was depicted by different line widths; the thicker the line, the stronger the NOE.

Chemical Shift Deviation

The Hα chemical shift deviation for each residue in the experimental peptide (ΔδHα(exp)) was the difference between the Hα chemical shift in the experimental peptide ($\delta H\alpha_{(exp)}$) and the corresponding Hα chemical shift in the fully unfolded reference peptide ($\delta H\alpha_{(U)}$) (equation 3). The Hα chemical shift deviation for each residue in the fully folded reference peptide (ΔδHα(F)) was the difference between the Hα chemical shift in the fully folded reference peptide ($\delta H\alpha_{(F)}$) and the corresponding Hα chemical shift in the fully unfolded reference peptide (equation 4). The HPDFZbbAgx peptide was assumed to be the fully folded reference peptide and the HPDUZbbAgx peptide to be the fully unfolded reference peptide.

$$\Delta\delta H\alpha(exp) = \delta H\alpha_{(exp)} - \delta H\alpha_{(U)} \quad \text{(equation 3)}$$

$$\Delta\delta H\alpha(F) = \delta H\alpha_{(F)} - \delta H\alpha_{(U)} \quad \text{(equation 4)}$$

Fraction Folded Population and Folding Free Energy

The fraction folded population and folding free energy (ΔG_{fold}) were derived from Hα chemical shift values, assuming a two-state system model for a β-hairpin in aqueous solution [10]. The fraction folded population and ΔG_{fold} for each residue were derived by using the equations 5 and 6, respectively. For each peptide, the fraction folded population and ΔG_{fold} were obtained by averaging the corresponding values for the residues at positions 2, 3, 9 and 10.

$$\text{Fraction folded (\%)} = \frac{\delta H\alpha(\text{exp}) - \delta H\alpha(U)}{\delta H\alpha(F) - \delta H\alpha(U)} \times 100\% \qquad \text{(equation 5)}$$

$$\Delta G_{fold} = -RTln\left(\frac{\delta H\alpha(\text{exp}) - \delta H\alpha(U)}{\delta H\alpha(F) - \delta H\alpha(\text{exp})}\right) \qquad \text{(equation 6)}$$

Double Mutant Cycle Analysis

The interaction free energy (ΔG_{int}) between charged residues Zbb and Agx in the HPDZbbAgx peptides was derived from double mutant cycle analysis using equation 7 [11]. The charged residues at positions 2 and 9 were individually substituted with Ala to give the reference peptides HPDAlaAgx and HPDZbbAla, respectively. The HPDAlaAla peptide was used as the reference peptide with Ala incorporated at both positions 2 and 9.

$$\Delta G_{int} = \left(\Delta G_{HPDZbbAgx} - \Delta G_{HPDAlaAla}\right) - \left(\Delta G_{HPDAlaAgx} - \Delta G_{HPDAlaAla}\right)$$
$$-\left(\Delta G_{HPDZbbAla} - \Delta G_{HPDAlaAla}\right)$$
$$= \Delta G_{HPDZbbAgx} - \Delta G_{HPDAlaAgx} - \Delta G_{HPDZbbAla} + \Delta G_{HPDAlaAla} \qquad \text{(equation 7)}$$

References

1. Atherton, E.; Fox, H.; Harkiss, D.; Logan, C. J.; Sheppard, R. C.; Williams, B. J. A mild procedure for solid phase peptide synthesis: use of fluorenylmethoxycarbonylamino-acids. *J. Chem. Soc., Chem. Commun.* **1978**, 537-539.
2. Fields, G. B.; Noble, R. L. Solid phase peptide synthesis utilizing 9-fluorenylmethoxycarbonyl amino acids. *Int. J. Pept. Protein Res.* **1990,** *35*, 161-214.
3. Volkmer-Engert, R.; Landgraf, C.; Schneider-Mergener, J. Charcoal surface-assisted catalysis of intramolecular disulfide bond formation in peptides. *J. Pept. Res.* **1998,** *51*, 365.
4. Bax, A., and Davis, D. G. MLEV-17-based two-dimensional homonuclear magnetization transfer spectroscopy. *J. Magn. Reson.* **1985,** *65*, 355-360.
5. Aue, W. P.; Bartholdi, E.; Ernst, R. R. Two−dimensional spectroscopy. Application to nuclear magnetic resonance. *J. Chem. Phys.* **1976,** *64*, 2229-2246.
6. Aksel A. Bothner-By, R. L. S., Jumee Lee, Christopher D. Warren, R. W. Jeanloz Structure determination of a tetrasaccharide: transient nuclear Overhauser effects in the rotating frame. *J. Am. Chem. Soc.* **1984,** *106*, 811-813.
7. Piotto, M.; Saudek, V.; Sklenar, V. Gradient-tailored excitation for single-quantum NMR spectroscopy of aqueous solutions. *J. Biomol. NMR* **1992,** *2*, 661-665.
8. Sklenar, V.; Piotto, M.; Leppik, R.; Saudek, V. Gradient-tailored water suppression for 1H-15N HSQC experiments optimized to retain full sensitivity. *J. Magn. Reson. Ser. A* **1993,** *102*, 241-245.
9. Kim, Y.; Prestegard, J. H. Measurement of vicinal couplings from cross peaks in COSY spectra. *J. Magn. Reson.* **1989,** *84*, 9-13.
10. Syud, F. A.; Espinosa, J. F.; Gellman, S. H. NMR-based quantification of β-sheet populations in aqueous solution through use of reference peptides for the folded and unfolded states. *J. Am. Chem. Soc.* **1999,** *121*, 11577-11578.
11. Horovitz, A. Double-mutant cycles: a powerful tool for analyzing protein structure and function. *Fold. Des.* **1996,** *1*, R121-R126.
